# Redox Cycling at Tellurium: Selective and Multiple Activation of Si─H Bonds in Common Organosilanes and SiH_4_

**DOI:** 10.1002/chem.202502141

**Published:** 2025-07-09

**Authors:** Martin Hejda, Lukáš Doležal, Milan Erben, Emanuel Hupf, Aleš Růžička, Jens Beckmann, Libor Dostál

**Affiliations:** ^1^ Department of General and Inorganic Chemistry University of Pardubice Studentská 573, CZ‐532 10 Pardubice Czech Republic; ^2^ Institut für Anorganische Chemie und Kristallographie Universität Bremen Leobener Straße 7 28359 Bremen Germany

**Keywords:** main group redox catalysis, multiple bond activation, silane, silicon, tellurium

## Abstract

Redox catalysis involving the tellurenyl triflate [2‐(*t*BuNCH)C_6_H_4_Te][OTf] was utilized for the activation of the common silanes Et_3_SiH, Ph_4–_ *_n_* SiH*_n_* (*n* = 1–3) as well as the difficult‐to‐handle gaseous SiH_4_. Furthermore, complex siloxanes containing multiple Si─H bonds were converted into siloxylated phenols, which are of high interest for the chemical industry. Insights into the catalytic cycle utilizing *para*‐quinones as both oxidants and substrates were provided by identification of key intermediates and by using in situ time‐resolved FTIR spectroscopy, NMR spectroscopy, single‐crystal X‐ray diffraction analysis, and DFT calculations. Based on this study, the catalytic cycle is initiated by a Si─H bond activation resulting in the formation of a ditelluride species and the corresponding silyl triflate. In turn, the ditelluride species is first oxidized by the presented quinone, forming an unprecedented tellurium‐based oxonium intermediate, resulting in the formation of the siloxylated phenols and liberation of the tellurium catalyst upon subsequent reaction with the silyl triflate. Our proposed catalytic cycle can be generalized for any silane used in this study.

## Graphical abstract

The tellurenyl triflate [2‐(*t*BuNCH)C_6_H_4_Te][OTf] effectively activates the Si─H bonds of Et_3_SiH, Ph_4–_ *_n_* SiH*_n_* (*n* = 1–3), SiH_4_, and complex H‐siloxanes and catalytically converts them in the presence of quinones into siloxylated phenols of high interest for the chemical industry.

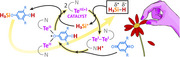

## 1 Introduction

Redox catalysis facilitated by main group elements is motivated by the urge to replace conventional transition metal catalysts that are often associated with severe economic and ecologic inadequacies.^[^ ^1^ ^]^ For a main group element to be catalytically active, different oxidation states of similar stability are required for oxidative addition and reductive elimination reactions to take place.^[^ ^2^ ^]^ The most studied elements for applications in main group catalysis involve phosphorus^[^ ^3^ ^]^ and bismuth,^[^ ^4^ ^]^ while other promising candidates, such as tellurium, are slowly emerging.^[^ ^5^ ^]^

**Scheme 1:**
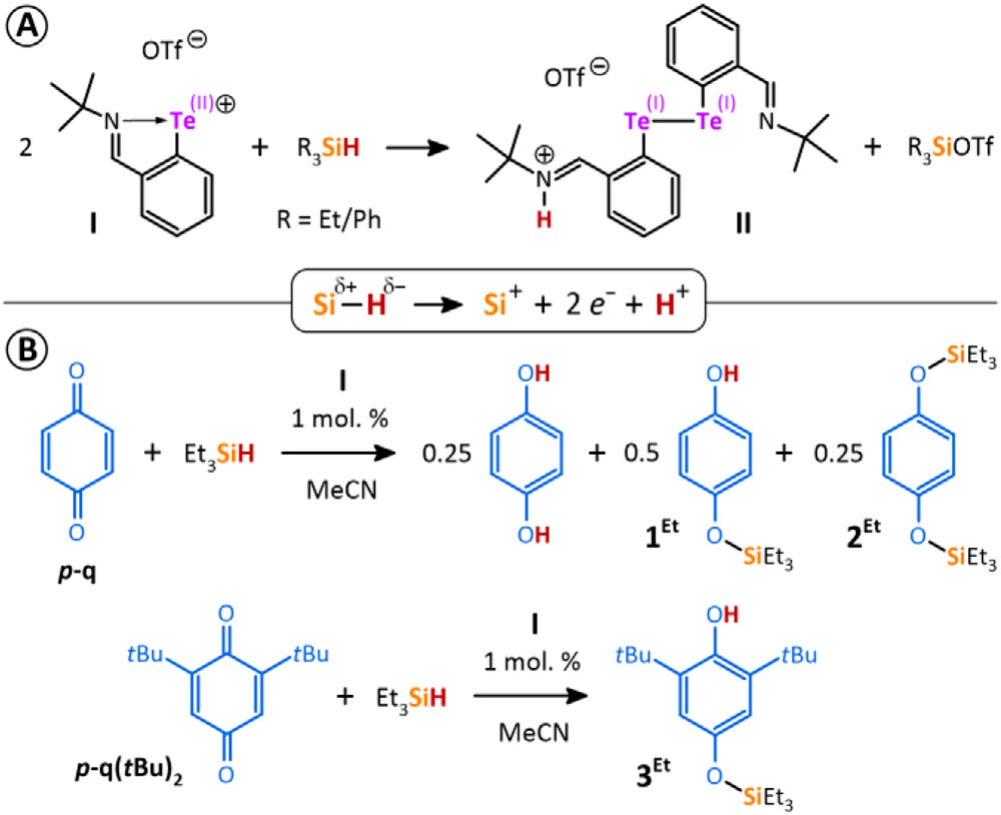
Presentation of published Si─H bond activation mediated by tellurenyl triflate **I** A) and its application in redox catalysis B).^[^ ^6^ ^]^

We have recently discovered the unique ability of the tellurenyl triflate **I** to activate Si─H bonds in primary silanes R_3_SiH in an unprecedented way, involving the conversion of R_3_SiH to a silylium cation R_3_Si^+^ (in the form of a silyl triflate; R = Et or Ph), a proton H^+^, and 2 *e* ^−^ that are reducing two tellurium atoms to yield the ditelluride **II** (Scheme 1A).^[^ ^6^ ^]^ This was further facilitated by using tellurenyl triflate **I** as a main‐group catalyst to silylate different *para*‐benzoquinones in a redox catalytic reaction (Scheme 1B).

Similar or the very same products (vide infra), such as the produced siloxylated phenols, have found widespread applications as high‐temperature stable and nonvolatile antioxidants^[^ ^7^ ^]^ and thermal stabilizers^[^ ^8^ ^]^ for organic polymers, as bridging molecules for polysiloxane‐polycarbonate block co‐condensates^[^ ^9^ ^]^ or as photosensitive layers in electrophotographic photoreceptors.^[^ ^10^ ^]^

We now expand the scope of the Si─H bond activation using catalyst **I** to silanes bearing up to four geminal Si─H bonds (ultimately using SiH_4_). It is to note that the activation of all four Si─H bonds in SiH_4_ under a catalytic regime has, to the best of our knowledge, not been reported before. A catalytic reaction mechanism is proposed based on time‐resolved Fourier transform infrared spectroscopy (FTIR), including 2D‐correlation analysis,^[^ ^11^ ^]^ variation of stoichiometric reactions, and the isolation of key reaction intermediates.

## 2 Results and Discussion

### 2.1 Expanding the Scope of Activated Silanes

**Figure 1:**
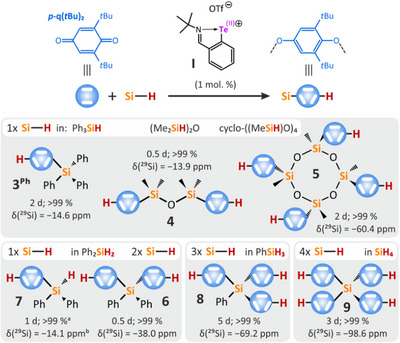
Catalytic activation of Si─H bond(s) of various silanes using tellurenyl triflate **I** in MeCN at room temperature. Number of activated Si─H bonds is given per a single Si center. For all reactions, loading of the catalyst **I** was 1 mol% per Si─H bond. For more details see Table S1. Conversion rates of the reaction are based on 1H NMR integration related to starting ***p*‐q(*t*Bu)_2_**. a) Contains about 9% of **6** due to nonselectivity of the reaction, yet all ***p*‐q(*t*Bu)_2_** was consumed. b) Observed as a doublet with ^1^ *J*(^29^Si,^1^H) = 218 Hz.

The sterically demanding 2,6‐di‐*tert*‐butyl‐1,4‐benzoquinone (***p*‐q(*t*Bu)_2_**) was used in the investigated redox catalysis reactions, due to the regioselectivity observed for the reaction with Et_3_SiH (Scheme 1B). To unravel the catalytic potential of the tellurenyl triflate **I**, ***p*‐q(*t*Bu)_2_** was reacted with different silanes bearing up to four Si─H bonds to give the siloxylated phenols **3**–**9** (Figure 1, see Table S1 for more details). All reactions were performed at r.t. with 1 mol.% catalyst loading per Si─H moiety using acetonitrile (MeCN) as the solvent. Using appropriate molar ratios between the used silane and ***p*‐q(*t*Bu)_2_**, all Si─H bonds were converted into the respective siloxylated phenols almost quantitatively (>99%), however, the reaction times vary from 12 hours (for 1,1,3,3‐tetramethylsiloxane, (Me_2_SiH)_2_O) to 5 days in the case of the phenylsilane PhSiH_3_. Notably, the parent silane SiH_4_ was readily converted in yields of > 99% after 3 days into Si(OC_6_H_4_(*t*Bu_2_)_2_OH)_4_ (**9**), representing a versatile synthetic protocol to access siloxylated phenols. These compounds might serve as useful building blocks for polymers or as additives for plastics (as already shown for **6** and **9,** showing polypropylene stabilization^[^ ^7^, ^8^ ^]^).

**Figure 2:**
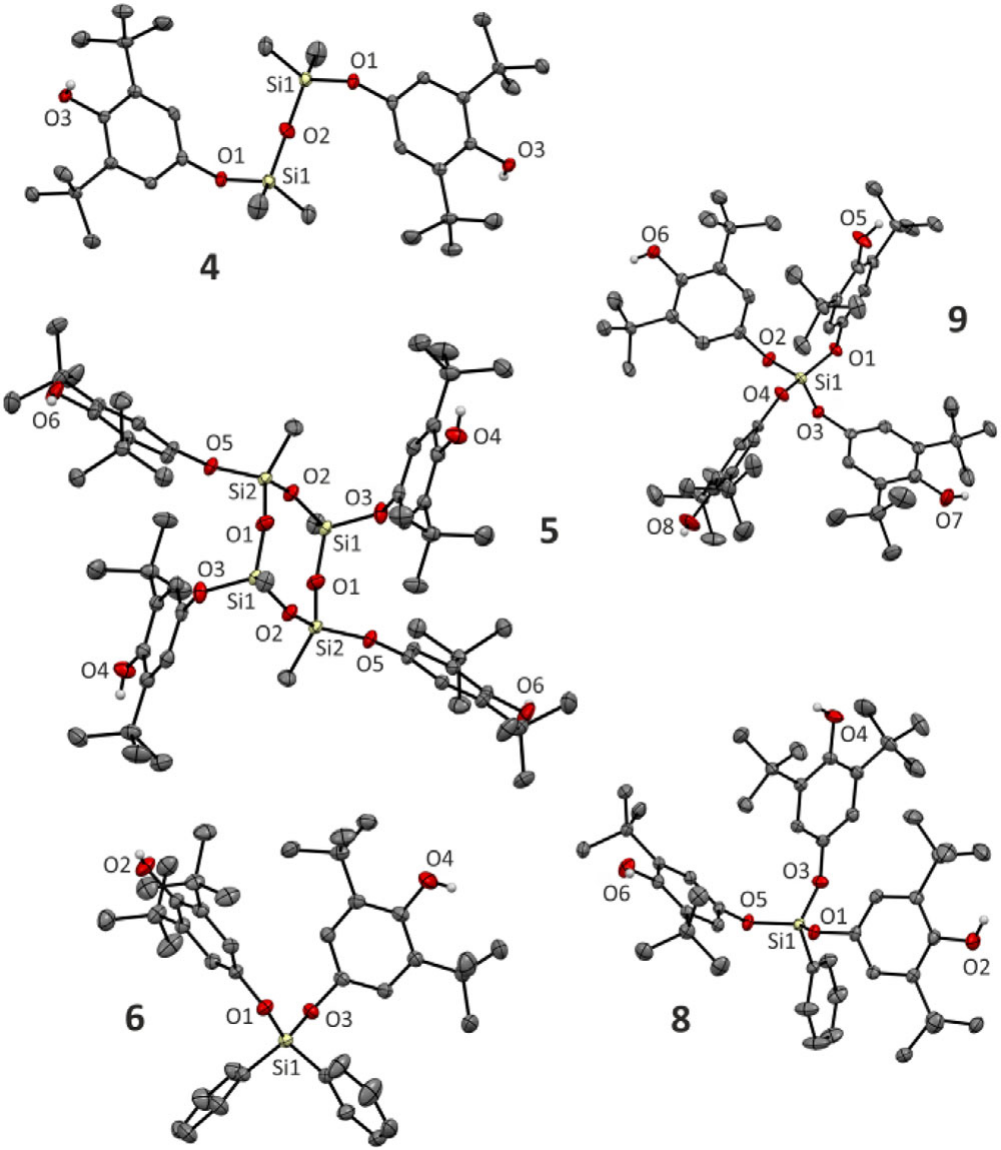
Molecular structures of products obtained by SC‐X‐ray diffraction. Hydrogen atoms are omitted for clarity except for OH protons.

All synthesized siloxylated phenols **3**–**9** were characterized by multinuclear NMR spectroscopy (^1^H, ^13^C, and ^29^Si; see Supporting Information for detailed assignment). A clear trend in δ(^29^Si) is observed within the series of compounds **3^Ph^** (−14.6 ppm), **6** (−37.9 ppm), **8** (−69.2 ppm), and **9** (−98.6 ppm), which are increasingly more shielded as a result of the presence of additional hydroquinone moieties. Except for compound **3^Ph^**, all molecular structures were also authenticated by single‐crystal X‐ray diffraction analysis (Figure 2; see Supporting Information for details).

In an effort to test whether the activation of the two Si─H bonds within Ph_2_SiH_2_ can also be performed in a stepwise manner, a catalytic reaction with ***p*‐q(*t*Bu)_2_** using a 1:1 molar ratio was examined.

Indeed, the monosubstituted **7** was obtained as the major product, yet, the reaction mixture was always contaminated by a small amount of **6**, the product of the double Si─H bond activation (∼9%), somewhat limiting the selectivity. Nevertheless, **7** could be separated from the reaction mixture by a fractional crystallization and was readily characterized by multinuclear NMR spectroscopy (see Supporting Information).

The presence of the intact Si─H bond was reflected in the corresponding ^29^Si NMR spectra by observation of a doublet at δ(^29^Si) = −14.1 ppm (^1^ *J*(^29^Si,^1^H) = 218 Hz). The formation of **7** suggests that the Si─H bonds are presumably cleaved consecutively in the catalytic process involving Ph_2_SiH_2_ (vide infra mechanistic studies). Notably, a similar experiment using PhSiH_3_ provided only a mixture of products, further indicating the limitations for a stepwise single, double, and triple Si─H bond activation.

### 2.2 In Situ FTIR Kinetic Studies

**Figure 3:**
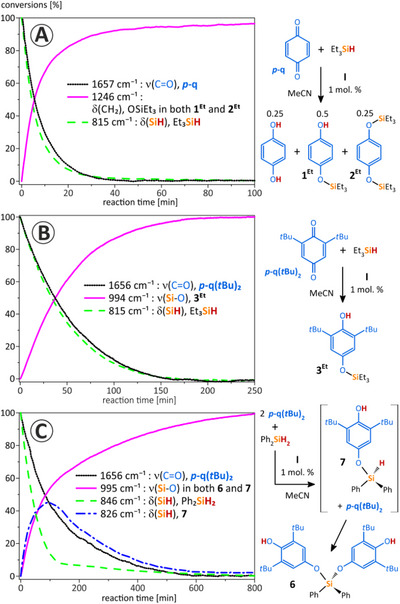
Time‐dependent changes in the intensity of IR bands measured in situ in given reaction mixtures A–C) (argon atmosphere, solvent MeCN, 1 mol.% of tellurenyl triflate **I** as catalyst per each Si─H bond.

**Scheme 2:**
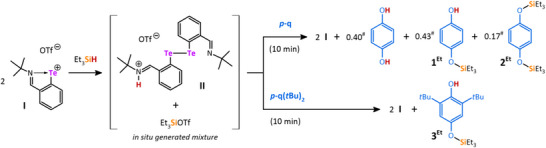
Stoichiometric reactions used for investigation of plausible catalytic cycle. All reactions gave the same results regardless of the solvent used (MeCN‐*d*_3_ or DCM‐*d*_2_). # In this stoichiometric reaction, the ratio of products is 0.40: 0.43: 0.17 and thus greatly differs from that observed at catalytic conditions (cf. Scheme 1: 0.25: 0.5: 0.25), in part due to hydrolysis leading to the formation of also 0.12 equiv. of Et_3_SiOSiEt_3_.^[^ ^16^ ^]^

**Scheme 3:**
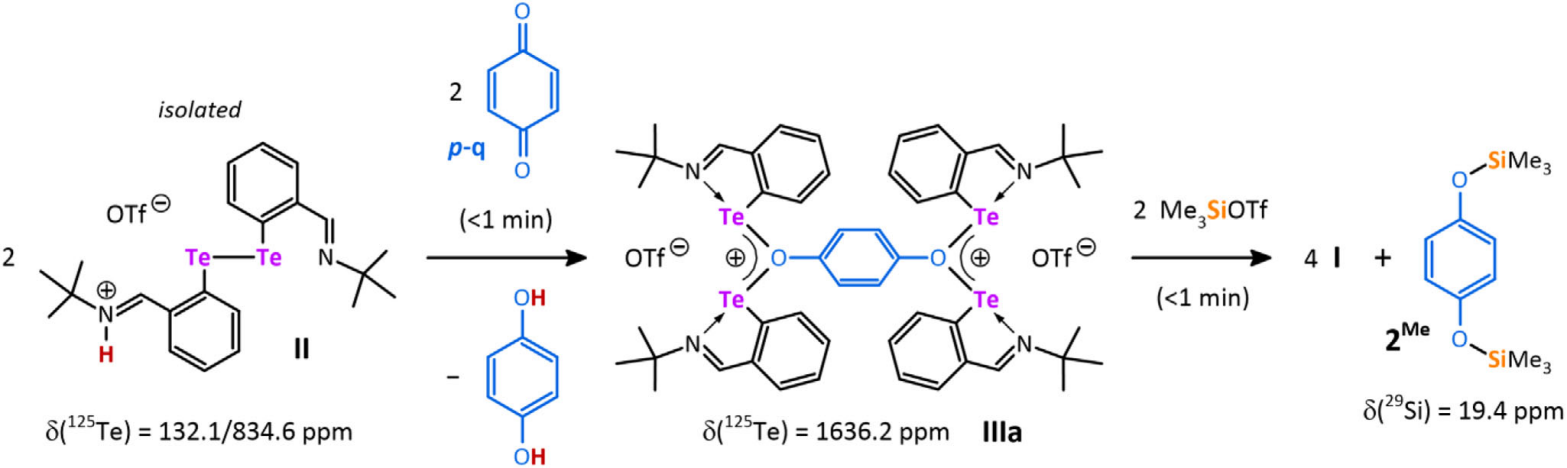
Stoichiometric reaction used for investigation of plausible catalytic cycle. All reactions gave the same results regardless of used solvent (MeCN‐*d*_3_ or DCM‐*d*_2_).

**Scheme 4:**
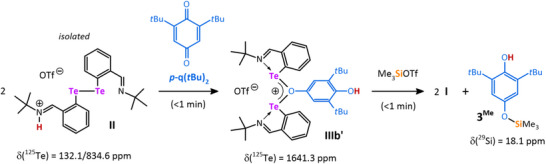
Stoichiometric reaction used for investigation of plausible catalytic cycle. All reactions gave the same results regardless of used solvent (MeCN‐*d*_3_ or DCM‐*d*_2_).

In an attempt to shed light on the catalytic mechanism, selected reactions were examined by time‐resolved FTIR spectroscopy in the region from 2200 to 800 cm^−1^ where chemically significant and assignable vibrations occurred. Analyzing the recorded spectra, characteristic absorptions of the silane and quinone precursors were detected together with bands corresponding to the products containing the O–Si–R fragments (R = Et/Ph).^[^ ^12^ ^]^ Figure 3 shows time‐dependent changes in the band intensities of the species monitored for three studied systems^[^ ^13^ ^]^: ***p*‐q** with Et_3_SiH leading to the hydroquinone, mono‐siloxyphenol **1^Et^,** and bis‐siloxybenzene **2^Et^** (Figure 3A, Scheme 1B); ***p*‐q(tBu)_2_** with Et_3_SiH leading to **3^Et^** (Figure 3B, Scheme 1B); and of ***p*‐q(tBu)_2_** with Ph_2_SiH_2_ in 2:1 molar ratio leading to **6** (Figure 3C). During the course of the reaction of ***p*‐q** with Et_3_SiH, a decrease in silane and ***p*‐q** concentration is observed as indicated by decreasing band intensities (δ_SiH_ at 815 cm^−1^) and ***p*‐q** (ν_C = O_ at 1657 cm^−1^) while the band at 1246 cm^−1^ (δ_CH2_ vibration of the OSiEt_3_ group) in both **1^Et^** and **2^Et^** increases in intensity (Figure 3A). From the development of the respective curves, it is evident that the consumption of the silane at the start of the reaction is somewhat faster than the decrease in the amount of quinone. This points to a quick generation of steady‐state concentration of the ditelluride **II** (Scheme 1A),^[^ ^14^ ^]^ while the back‐oxidation to tellurenyl triflate **I** by the quinone is a slower process. This phenomenon corresponds visually to the observed color changes during stoichiometric reactions of the isolated compounds (Schemes 2–4, see Supporting Information for procedures). Analogous findings in IR experiments were observed for the reaction of quinone ***p*‐q(*t*Bu)_2_** with Et_3_SiH (Figure 3B). In this case, analysis of complex IR spectra is much more straightforward, as **3^Et^** is the sole product formed. However, in this case, the reaction with the sterically hindered ***p*‐q(*t*Bu)_2_** proceeds significantly slower, probably due to the propensity of the silylium ion to attack the nonsterically hindered C═O function of ***p*‐q(*t*Bu)_2_**. Notably, a direct observation of the Si─O stretching mode (994 cm^−1^) of **3^Et^** species in the corresponding IR spectrum was possible.

The reaction of Ph_2_SiH_2_ with ***p*‐q(*t*Bu)_2_** in a molar ratio of 1:2 yielded the disubstituted product **6**.

**Figure 4:**
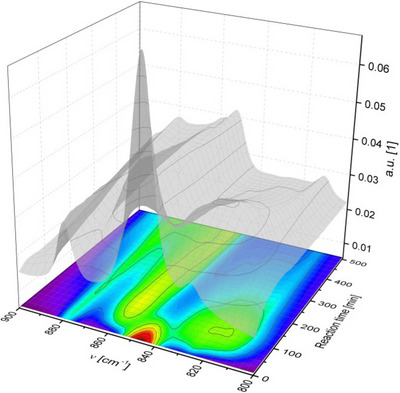
3D surface plot of the IR region 900–800 cm−1 recorded during the catalyzed reaction of ***p*‐q(*t*Bu)_2_** with Ph_2_SiH_2_ corresponding to data from the 2D plot in Figure 3C (argon atmosphere, 2:1 molar ratio, MeCN solvent, 1 mol.% of catalyst **I** per each Si─H bond) leading to formation of **6** via transient formation of **7**.

However, bands that could be assigned to the presence of the single Si─H bond activated intermediate (i.e., **7**) with one remaining intact Si─H bond were observed as well (and proved by measuring IR spectra for independently isolated **7** in MeCN showing δ_SiH_ at 826 cm^−1^) on inspection of time‐resolved FTIR spectra. The 3D plot of region 900–800 cm^−1^, where the characteristic absorptions of δ_SiH_ modes of the starting Ph_2_SiH_2_ (846 cm^−1^) and intermediate **7** (826 cm^−1^) were detected, is shown in Figure 4.

The increase in the concentration of the intermediate **7** is associated with a very rapid consumption of the starting silane, and the maximum is reached 89 minutes from the start of the reaction. After that, the concentration of **7** decreases and the consumption of silane slows down.

In an effort to gain a more detailed insight into the mechanism of the presented catalyzed redox transfer of silane on quinones, the time‐resolved infrared spectra were subjected to a generalized 2D correlation analysis (2DCoS). This method enables simplification of complex spectra consisting of overlapped peaks, enhancement of spectral resolution by spreading peaks over the second dimension, and determination of sequential order for followed dynamic processes.^[^ ^11^ ^]^ Briefly, cross‐correlated dynamic changes in infrared bands at given wavenumbers (ν_1_, ν_2_) produce synchronous (Φ) and asynchronous (Ψ) 2D‐IR spectra. The sequential order of intensity variations at given wavenumbers (ν_1_, ν_2_) can be derived from signs of the cross‐peaks following Noda's rules.^[^ ^11^ ^]^ Data extracted from synchronous and asynchronous 2D‐correlation maps (Figures S58–S63) for all systems studied, is summarized in Table S2. Applying Noda's rules,^[^ ^11^ ^]^ the following conclusions can be drawn: When Et_3_SiH reacts with ***p*‐q** or ***p*‐q(*t*Bu)_2_**, a loss of silane is observed first, and then it is followed by the consumption of quinone. Simultaneously, with the decrease of quinone, the concentration of Et_3_SiO‐substituted products (both **1^Et^** and **2^Et^**) increases. The reaction of Ph_2_SiH_2_ with ***p*‐q(*t*Bu)_2_** (1:2) is initiated by the rapid decrease in Ph_2_SiH_2_ concentration, which is followed by the decrease of quinone in the reaction mixture, and the intermediate product **7** is formed. **7** is consumed during the course of the reaction to form the final **6**.

### 2.3 Mechanistic Study on Isolated Intermediates

As proven by the FTIR study, vide supra, the activation of Si─H functions in silanes precedes the Si─O bond formation during the catalytic cycle with **I**. This reaction sequence was further investigated via control experiments reacting Et_3_SiH with ***p‐*q** or ***p*‐q(*t*Bu)_2_** (Scheme 2).

Without the tellurenyl triflate catalyst **I**, Et_3_SiH showed no reaction with either ***p‐*q** or ***p*‐q(*t*Bu)_2_** (Figure S25). Moreover, using stoichiometric conditions, **I** was inert toward both quinones (Figure S26),^[^ ^15^ ^]^ but reacted cleanly with Et_3_SiH to form the ditelluride **II** and 1 equiv. of Et_3_SiOTf (see Scheme 1A).^[^ ^6^ ^]^ This undermines that the Si─H bond activation in silanes is the initial step of the catalytic cycle. Importantly, an addition of ***p‐*q** to such an in situ generated mixture of ditelluride **II** and Et_3_SiOTf (i.e., products of the Si─H bond activation) produced the final products, namely the mono‐siloxyphenol **1^Et^** and bis‐siloxybenzene **2^Et^**, analogously to the catalytic process (Scheme 2), concomitantly with the formation of 2 equiv. of tellurenyl triflate **I** (Figures S35, S36).

We further analyzed the proposed oxidation of ditelluride **II** to tellurenyl **I** during reaction with ***p‐*q**.^[^ ^17^ ^]^ Reacting the isolated compound **II** with ***p‐*q** in a 1:1 molar ratio and in the absence of Et_3_SiOTf gave hydroquinone along with a unique tetranuclear bis(oxonium) complex **IIIa** comprising an unprecedented ditellurium‐substituted μ_3_‐oxygen moiety, that precipitates from this stoichiometric reaction (Scheme 3; Figure S37). Interestingly, subsequent treatment of the bis(oxonium)species **IIIa** with Me_3_SiOTf (used as an analogue of Et_3_SiOTf) provided solely the bis‐siloxybenzene **2^Me^** with δ(^29^Si) = 19.4 ppm, with complete recovery of tellurenyl triflate **I** (Scheme 3; Figures S43–S45).

Reacting the ditelluride **II** with the bulkier ***p*‐q(*t*Bu)_2_** led to the formation of the dinuclear mono(oxonium) species **IIIb’**, which gave the monosiloxylated phenol **3^Me^** after treatment with Me_3_SiOTf, while the tellurenyl triflate **I** is completely recovered (Scheme 4; Figures S53, S54). The structure of **IIIb’** also explains the regioselectivity obtained in catalytic reactions with ***p*‐q(*t*Bu)_2_**, as the Te‐atoms in **IIIb’** prefer the sterically less demanding side of the hydroquinone, while the H‐atom ends up at the OH group between the *t*Bu groups. Consequently, the silyl fragment also binds at the noncrowded part of the quinone. The depicted structures of both bis(oxonium) and mono(oxonium) complexes **IIIa** and **IIIb’** in Schemes 3 and 4 are fully supported by multinuclear NMR spectroscopy (^1^H, ^13^C, and ^125^Te).

**Figure 5:**
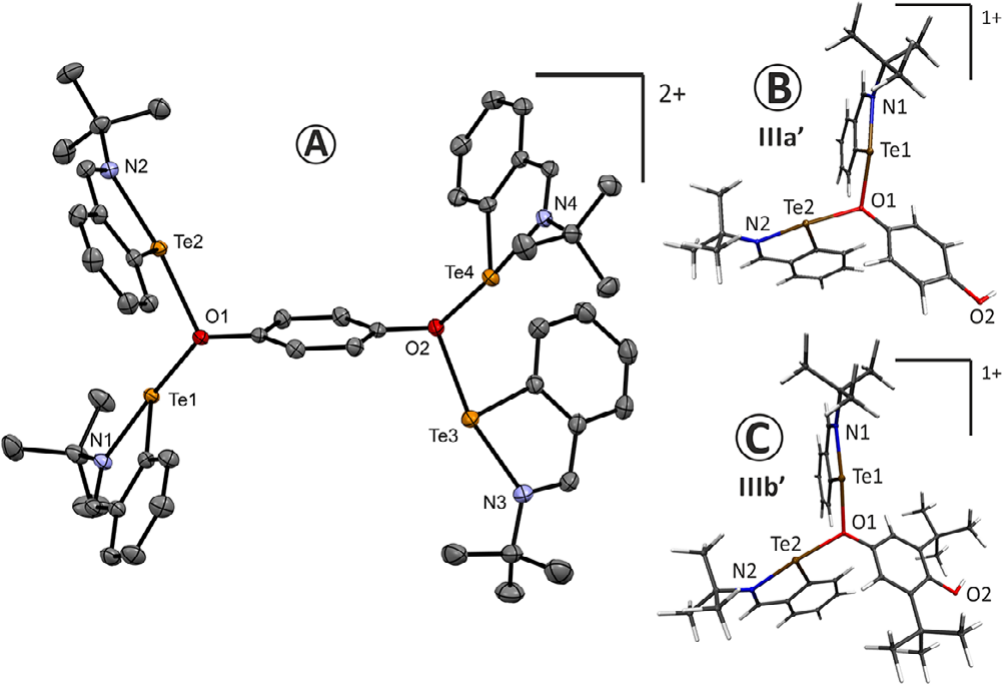
Molecular structure of the dicationic part of **IIIa** as determined by SC‐X‐ray diffraction (A; both triflate anions are omitted for clarity; the thermal ellipsoids are given with 50% probability) and optimized structures of the monocationic parts of **IIIa´** and **IIIb’** (B and C, respectively).

The ^1^H NMR spectrum of **IIIb’** in DCM‐*d*_2_ shows a singlet at δ(^1^H) = 4.83 ppm, which was assigned to the O*H* group, being in close proximity to the *t*Bu groups in the *ortho*‐position on the basis of a ^1^H‐^1^H NOESY experiment. ^125^Te{^1^H} NMR spectra of **IIIa** and **IIIb’** reveal only one signal for both compounds at 1636.2 and 1641.3 ppm, respectively, which is consistent with the symmetric coordination of the tellurium atoms (Figures S42, S52). Both values are slightly deshielded compared to **I** (cf. 1735.2 ppm).^[^ ^18^ ^]^ The molecular structure of **IIIa** is depicted in Figure 5 along with the optimized structure of **IIIa´** (elusive) and **IIIb’** (no suitable single crystals were obtained).

Like in solution, the solid‐state structure of **IIIa** reveals that two tellurium atoms are coordinating μ_3_‐oxygen atoms. The Te─O bond lengths are in the range 2.296(1)–2.358(1) Å, which is noticeably elongated compared to the corresponding bond lengths in structurally related neutral telluroxane ((*o*‐Ph‐N═N‐C_6_H_4_Te)_2_O; (Te–O) = 2.026(3)/2.007(3) Å)^[^ ^19a^ ^]^ with μ_2_‐oxygen moiety within the Te^II^–O–Te^II^ fragment, yet it is much shorter in comparison to the ones in dimeric neutral 2,2′‐biphenylylene‐2‐biphenylylphenoxytellurane with Te^IV^‐(μ‐OAr)‐Te^IV^ moiety (Ar = 2,4,6‐Cl_3_C_6_H_2_; (Te–O) = 2.762(2)‐2.787(2) Å).^[^ ^19b^ ^]^ The intramolecular *N*‐coordination in **IIIa** gives rise to a Te–N bond length of 2.175(1)–2.200(1) Å, *cf*. (Te–N) = 2.113(1) Å^18^ for the starting tellurenyl triflate **I** and (Te–N) = 2.203(2) Å for a parental covalent tellurenyl chloride.^[^ ^20^ ^]^ The special arrangement around the μ_3_‐oxygen atoms is trigonal planar with Te–O–Te bond angles of 107.27 and 109.72°.

**Scheme 5:**
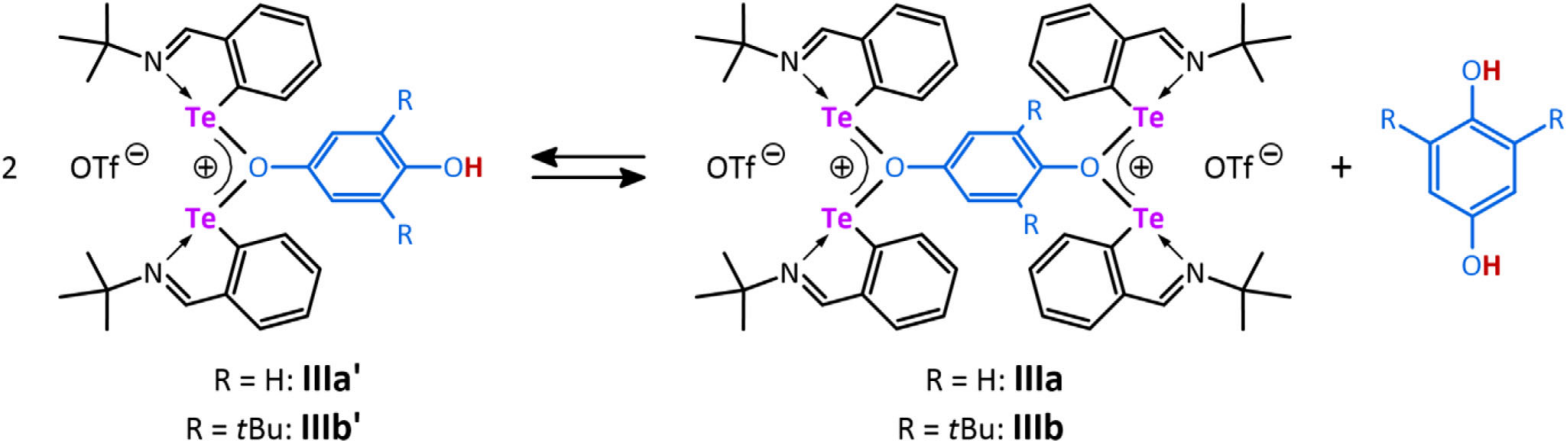
Hypothetical redistribution of dinuclear mono(oxonium) species **(IIIa’**/**IIIb’**) into tetranuclear bis(oxonium) species (**IIIa**/**IIIb**) and respective hydroquinone.

We speculate that a similar dinuclear mono(oxonium) species to **IIIb’**, that is, **IIIa’** (Figure 5B), might also play a role in the silylation of the nonsubstituted quinone (***p*‐q**), as observed in the reaction of tellurenyl triflate **I** with Et_3_SiH and ***p*‐q**, as both, mono‐siloxyphenol **1^Et^** and bis‐siloxybenzene **2^Et^** are formed (Schemes 1 and 3). To support this idea, we computed the Gibbs free energy of the hypothetical redistribution reaction shown in Scheme 5. Indeed, the redistribution is only slightly exergonic (ΔG = −0.7 kJ mol^−1^) upon inclusion of acetonitrile as solvent environment, indicating that a dynamic equilibrium is feasible. The rather low solubility at higher concentrations as well as packing effects in the solid state may also favor **IIIa** (see Scheme 3).

**Figure 6:**
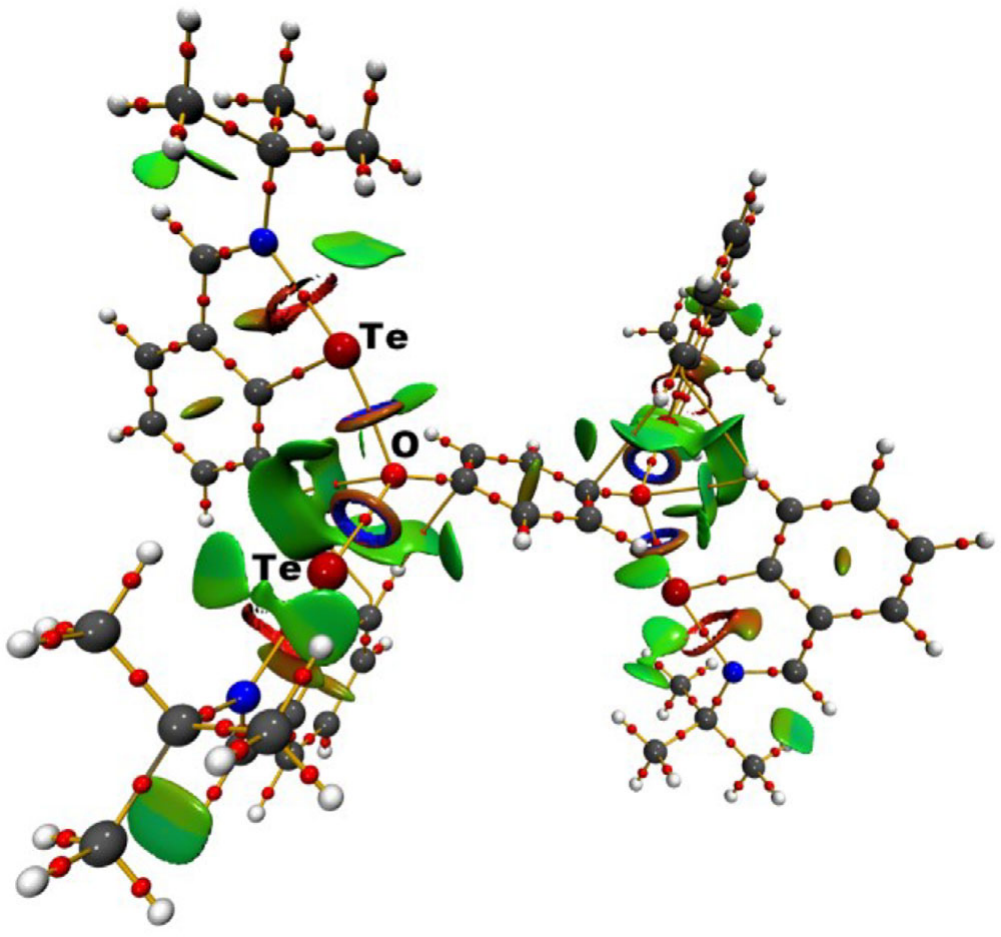
AIM molecular graph of bis(oxonium) species **IIIa** with bond critical points as red spheres and bond paths in orange as well as NCI iso‐surfaces at s(r) = 0.5 color coded with sign(*λ*2)ρ in a. u. Blue surfaces refer to attractive forces and red to repulsive forces. Green indicates weak interactions.

In contrast, the redistribution of the *t*Bu‐analogue is computed to be endergonic (ΔG = 57.1 kJ mol^−1^), shifting the equilibrium on the dinuclear side (**IIIb’**). Both ΔG values are therefore consistent with the experimental results. The compound **IIIa** featuring the ditellurium‐substituted μ_3_‐oxygen moieties was analyzed by an Atoms‐In‐Molecules (AIM) analysis.^[^ ^21^ ^]^ The Te–O interactions show signatures of both, covalent and ionic contributions. Covalent contributions are indicated by negative total energy densities over ρ(r)bcp ratios (H/ρ(r)bcp = −0.18 to −0.20 a.u.), whereas ionic bonding aspects are shown by positive Laplacians ((∇^2^ρ(r)bcp = 3.7 e Å^−5^) and kinetic energy densities over ρ(r)bcp ratios (G/ρ(r)bcp = 0.79 to 0.80 a.u.). The covalent bonding can be visualized by the inspection of the noncovalent interaction (NCI)^[^ ^22^ ^]^ *iso*‐surface, which shows torus‐shaped areas along the Te–O axes (Figure 6). The Te(II)–O interactions in the bis(oxonium) species are yet very similar to the Te(IV)–O interactions in *peri*‐substituted acenaphthenes featuring Ph_2_P(O) and Te(IV) fragments.^[^ ^23^ ^]^

The final attention was devoted to a stepwise activation of the two Si─H bonds in Ph_2_SiH_2_ and related silanes. As mentioned above, the catalytic reaction between Ph_2_SiH_2_ and ***p*‐q(*t*Bu)_2_** (1:1) allowed the isolation of **7** (Scheme S6).

**Scheme 6:**
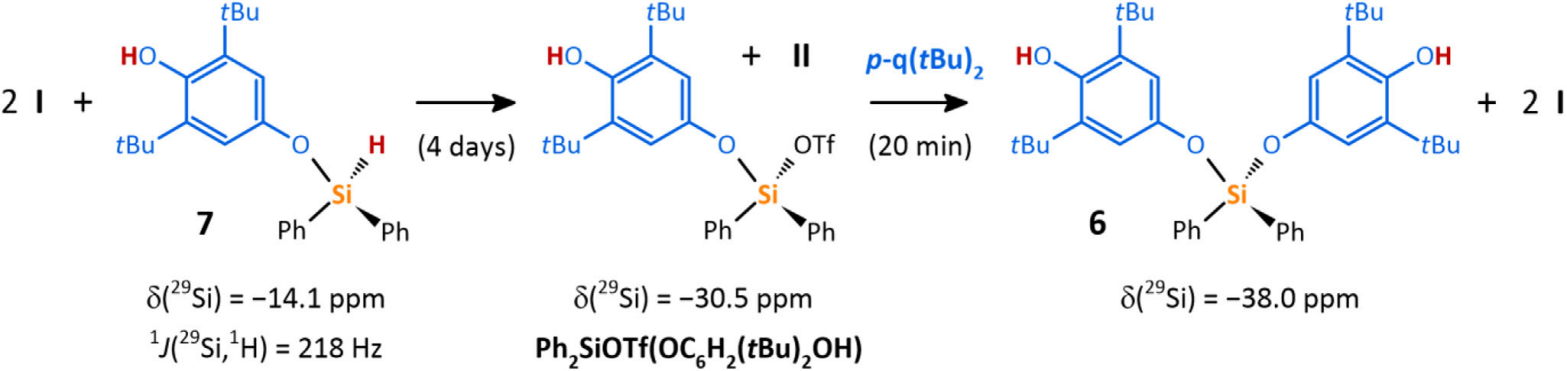
Stoichiometric reactions used for investigation of plausible catalytic cycle. All reactions gave the same results regardless of used solvent (MeCN‐*d*_3_ or DCM‐*d*_2_).

To recognize, if **7** can enter the next catalytic cycle (as suggested by in situ FTIR experiments, vide supra) and thereby prove a stepwise nature of geminal Si─H bonds activation in more complex silanes, **7** was treated with 1 equiv. of ***p*‐q(*t*Bu)_2_** using **I** in a catalytic amount (1 mol %). Importantly, this reaction cleanly afforded the expected product **6** after a day of reaction time (Scheme S7). On the way to **6**, the silylium species **Ph_2_SiOTf(O‐C_6_H_2_(*t*Bu)_2_OH)** should be formed as a key intermediate by the activation of the remaining Si─H bond in **7** by tellurenyl triflate **I**. Most importantly, **Ph_2_SiOTf(O‐C_6_H_2_(*t*Bu)_2_OH)** (δ(^29^Si) = −30.5 ppm) could be indeed obtained from a stoichiometric reaction between 2 equiv. of tellurenyl triflate **I** and **7** (Scheme 6, Figures S55–S57) along with expected ditelluride **II**. Finally, an addition of ***p*‐q(*t*Bu)_2_** to this mixture of **Ph_2_SiOTf(O‐C_6_H_2_(*t*Bu)_2_OH)** and **II**, cleanly provided **6** and 2 equiv. of **I** (Scheme 6), thereby unambiguously proving the proposed reaction sequence.

**Scheme 7:**
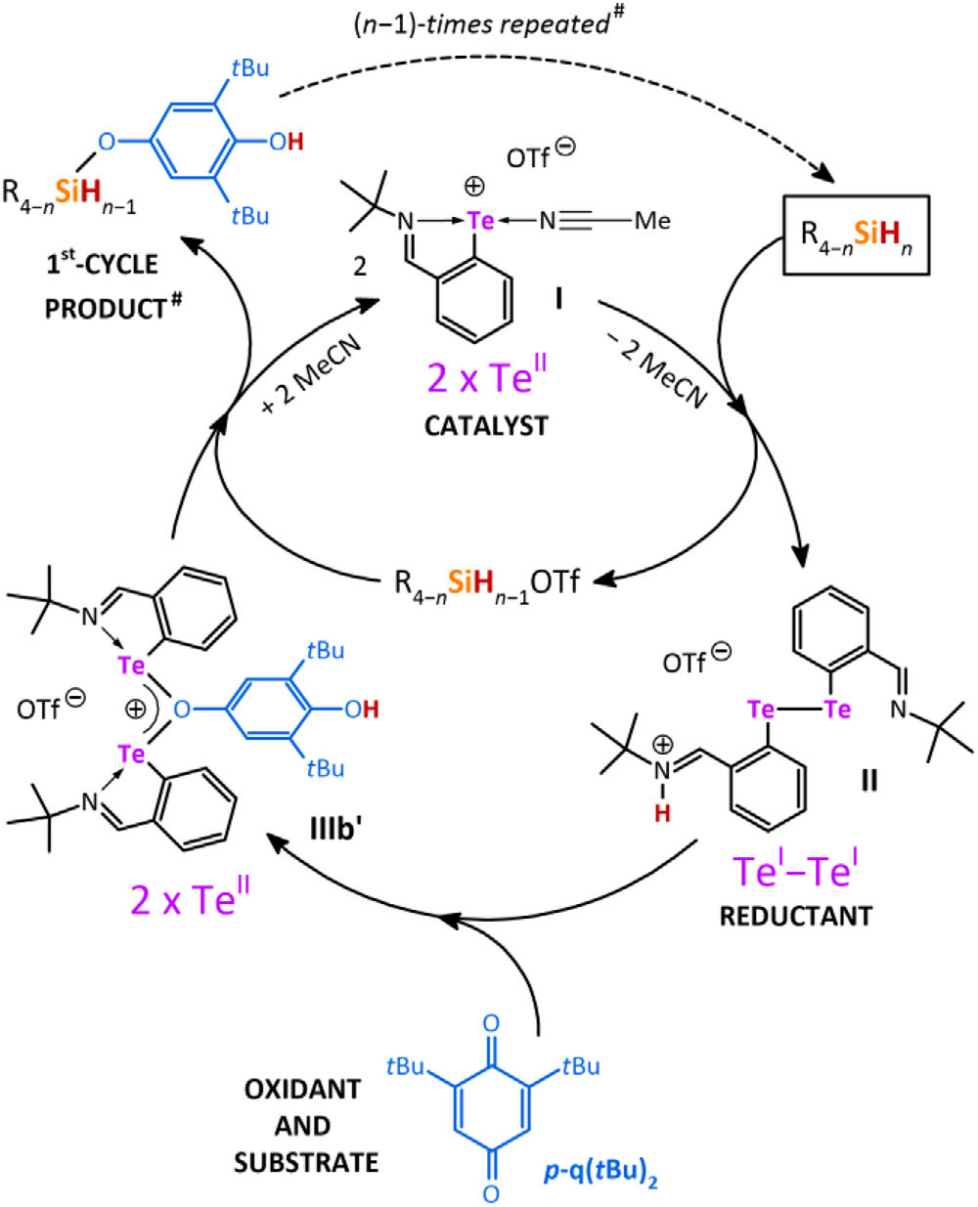
Proposed catalytic cycle utilizing redox Si─H bond activation(s) in silanes of type R_4__−__*n*_SiH_*n*_ using tellurenyl triflate **I** as the catalyst with ***p*‐q(*t*Bu)_2_** as both oxidant and substrate.^[^ ^24^ ^]^ # If the number (*n* − 1) of Si─H bonds contained in the first‐cycle product is > 0 and at least an equiv. of ***p*‐q(*t*Bu)_2_** is presented, this first‐cycle product enters and finishes another full catalytic cycle. Consequently, if *n* equiv. of ***p*‐q(*t*Bu)_2_** are present in the reaction mixture, the whole catalytic cycle is then (*n* − 1)‐times repeated until all H atoms in all Si─H bonds are replaced by ‐OC_6_H_2_(*t*Bu)_2_OH substituents.

Based on these findings, we claim that in silanes containing more than one Si─H bond, the catalytic cycle is stepwise repeated each time with the formation of different silylium species. Taking SiH_4_ as an extreme example, one can consider a successive formation of four transient silyl triflates, that is, **R_4_**_−_ ***_n_* SiH*_n_***_−_ **_1_OTf** (where **R** = **HO‐C_6_H_2_(*t*Bu)_2_O**, *n* = 1−4; Scheme 7). In turn, each of these stepwise generated silyl triflates reacts with 1 equiv. of **IIIb’** (being in situ generated by the reaction of **II** and ***p*‐q(*t*Bu)_2_** as shown in Scheme 4) until the last representative of triflate **(HO‐C_6_H_2_(*t*Bu)_2_O)_3_SiOTf** is converted into the final product **9** (i.e., **(HO‐C_6_H_2_(*t*Bu)_2_O)_4_Si**). To generalize, any silane of the type **R_4−_ *_n_* SiH*_n_*** used in this study, undergoes *n‐*times (*n* = 1–4) subsequent Si─H bond activations (Scheme 7) giving *n* silylium triflates along with *n* equiv. of ditelluride **II**, while the presence of the appropriate amount of ***p*‐q(*t*Bu)_2_** justifies the re‐oxidation of **II**, making the whole process catalytic. Based on all the above‐mentioned facts, we proposed a catalytic cycle (Scheme 7), which can be generalized for any silane of type R_4−_ *_n_* SiH*_n_* (*n* = 1–4) used in this study.

## 3 Conclusion

In this work, we focused on main group redox catalysis involving the activation of multiple Si─H bonds using tellurium at different oxidation states. This redox catalysis includes common silanes such as Et_3_SiH, Ph_4–*n*_SiH*_n_* (*n* = 1–3) and even gaseous SiH_4_, which is, to the best of our knowledge, an unprecedented activation to turn them into valuable economic products, namely siloxylated phenols. The process is also feasible for more complex siloxanes containing multiple Si─H bonds, such as 1,1,3,3‐tetramethyldisiloxane and 2,4,6,8‐tetramethylcyclotetrasiloxane. We have shed light on the mechanism of this Si─H bond activation by isolation of key intermediates and by monitoring of the process using in situ FTIR and NMR spectroscopy. A new class of oxonium ion involving tellurium has been identified and fully characterized by crystallography. Further insight into the nature of the intermediates was derived from DFT calculations. This study shows that our tellurium species hold similar potential in bond activation as phosphorus^3^ and bismuth^4^ which are at present at the forefront of the research.

## Supporting Information

The authors have cited additional references within the Supporting Information (Refs. [25, 26, 27, 28, 29, 30, 31, 32, 33, 34, 35]).

## Conflict of Interest

The authors declare no conflict of interests.

## Supporting information



Supporting Information

## Data Availability

The data that support the findings of this study are available from the corresponding author upon reasonable request.
