# Supplementary material for: Redox Cycling at Tellurium: Selective and Multiple Activation of Si─H Bonds in Common Organosilanes and SiH4
Source: Chemistry. 2025 Jul 9;31(43):e202502141. doi: 10.1002/chem.202502141 (PMC12319385; doi:10.1002/chem.202502141)
Supplement: Supplementary file 1 — Supporting Information [file CHEM-31-e202502141-s001.docx]

**Supporting Information**

**Contents:**

| **Experimental details** | S2 |
| --- | --- |
| **Catalytic syntheses and corresponding NMR data** |  |
| - **Table S1:** Overview of products and used stoichiometry | S4 |
| - Considered redox changes | S5 |
| - Catalytic synthesis of compound **3^Ph^** using **I** as catalyst | S5 |
| - Catalytic synthesis of compound **4** using **I** as catalyst | S8 |
| - Catalytic synthesis of compound **5** using **I** as catalyst | S11 |
| - Catalytic synthesis of compound **6** using **I** as catalyst | S14 |
| - Catalytic synthesis of compound **7** using **I** as catalyst | S17 |
| - Catalytic synthesis of compound **6** from compound **7** using **I** as catalyst | S21 |
| - Catalytic synthesis of compound **8** using **I** as catalyst | S22 |
| - Catalytic synthesis of compound **9** using **I** as catalyst | S25 |
| **Mechanistic study** |  |
| - Control experiments at stochiometric reaction conditions | S30 |
| - A role of proton scrambling in **II** for the catalysis | S31 |
| - NMR spectra from stoichiometric reaction of 1:1 mixture of **II** and Et_3_SiOTf with ***p*-q** | S41 |
| - NMR spectra from reaction of **II** with ***p*-q** leading to **IIIa** and hydroquinone | S43 |
| - NMR spectra from reaction of **IIIa** with two eq. of Me_3_SiOTf | S49 |
| - NMR spectra from stoichiometric reaction of 1:1 mixture of **II** and Et_3_SiOTf with ***p*-q(*t*Bu)_2_** | S52 |
| - NMR spectra from reaction of **II** with ***p*-q(*t*Bu)_2_** leading to **IIIb’** | S54 |
| - NMR spectra from reaction of **IIIb’** with Me_3_SiOTf | S59 |
| - NMR spectra from stoichiometric activation of Si-H bond in compound **7** – formation of silyl triflate **Ph_2_SiOTf(O-C_6_H_2_(tBu)_2_OH)** | S62 |
| - 2D-FTIR correlation maps | S66 |
| **DFT computations** | S70 |
| **Crystallographic data for studied compounds** | S72 |
| **Additional references** | S75 |

***Experimental details***

**General procedures.** All air- and moisture-sensitive manipulations were carried out under argon (99.999 %) using only Schlenk tube techniques enhanced with utilization of rubber septa and cannula for transfer of liquids and for their filtration (via filtration cannulas). All glassware was flame-annealed in vacuum prior to use. All solvents for reactions were dried using Pure Solv–Innovative Technology equipment. The starting compounds: *para*-benzoquinone (***p*-q**) (>98%), 2,6-di-*tert*-butyl-1,4-benzoquinone (***p*-q(*t*Bu)_2_**) (98%), Et_3_SiH (99%), Ph_3_SiH (97%), Ph_2_SiH_2_ (97%), PhSiH_3_ (97%), SiCl_4_ (99.998%), 1,1,3,3-tetramethyldisiloxane (97%), 2,4,6,8-tetramethylcyclotetrasiloxane (>99.999%), LiAlH_4_ (>97%), HOTf (>99%) and Me_3_SiOTf (97%) were obtained from Sigma-Aldrich. Compound **I** was synthesized according to our recently published procedure.[^[18]^](#_ENREF_1)

For mechanistic study when pure compound **II** was needed, the compound was synthetized *in situ* from its deprotonated form **IIa** by reaction with neat HOTf in 1:1 molar ratio in the given solvent (MeCN-*d*_3_ or DCM-*d*_2_), similarly to our published procedure.^[6]^ The reaction is completed within seconds. Such procedure is beneficial because storage of compound **II** is challenging due to its high sensitivity to oxygen, while compound **IIa** is much less sensitive to traces of oxygen and can be stored in large quantity under argon atmosphere for at least two years. Compound **IIa** was synthesized according to our recently published procedure.^[6]^


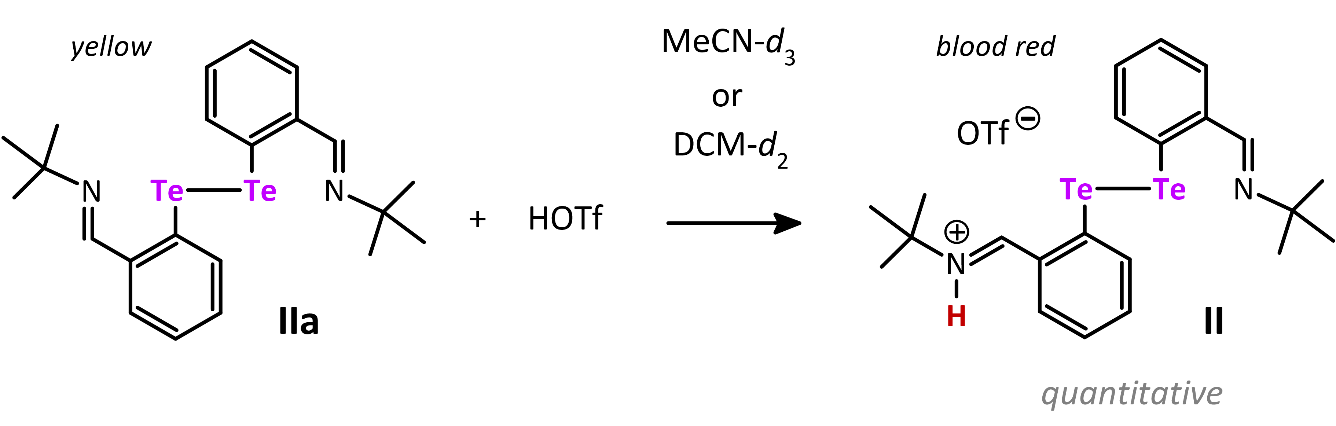


Scheme S1: Procedure for synthesis of compound II from compound IIa.

**Solution NMR spectroscopy.** ^1^H, ^13^C, ^15^N, ^29^Si and ^125^Te NMR spectra were recorded on Bruker Avance 500 MHz spectrometer, using a 5 mm tunable broad-band probe. Appropriate chemical shifts in ^1^H and ^13^C NMR spectra are given relative to the residual signals of the solvent [CD_2_Cl_2_: δ(^1^H) = 5.32 ppm and δ(^13^C) = 54.0 ppm; MeCN-*d*_3_: δ(^1^H) = 1.94 ppm and δ(^13^C) = 118.69 ppm], ^15^N NMR spectra were related to external neat nitromethane [δ(^15^N) = 0.0 ppm], ^29^Si NMR spectra were related to dilute TMS in CDCl_3_ [δ(^29^Si) = 0.0 ppm] and ^125^Te NMR chemical shifts are referenced to external CDCl_3_ solution of Ph_2_Te_2_ [δ(^125^Te) = 422 ppm relative to Me_2_Te]. NMR solvents (CD_2_Cl_2_ (99.96% D) and MeCN-*d*_3_ (99.80% D) were dried by staying over activated molecular sieves (3Å, 20 wt%) for week and then degassed by three cycles freeze*-*pump*-*thaw. NMR samples were prepared under argon and measured in annealed flame-sealed NMR tubes. The full assignment of all signals in all measured NMR spectra was managed with the help of various techniques including ^1^H, ^1^H-^1^H COSY, ^1^H-^1^H NOESY ^13^C{^1^H} APT, ^1^H-^13^C HSQC and ^1^H-^13^C HMBC. ^15^N NMR chemical shifts and ^1^*J*(^15^N,^1^H) were obtained from ^1^H-^15^N HMBC spectra (value of cnst13 = 5 Hz).

**Time-resolved FTIR Raman spectroscopy.** FTIR spectra were run on Nicolet iS50 spectrometer equipped with Fiber Optic Si-ATR dip-probe. Series of obtained IR spectra were processed (1D analysis) using Omnic 9 software and SpectraCorr 1.1 (2DCoS analysis) by Thermo Fisher Scientific Inc.

***Catalytic syntheses and corresponding NMR data***

**Table S1:** Overview of products from redox catalytic activation of Si−H bond(s) in various silanes and ***p*-q(*t*Bu)_2_** as both, oxidant and substrate, using **I** as catalyst in MeCN at room temperature. ^a^ Loading of the catalyst **I** was 1 mol. % *vs* one Si−H bond. ^b^ Conversion of the reaction is based on ^1^H NMR integration related to starting ***p*-q(*t*Bu)_2_**. ^c^ Observed as doublet with ^1^*J*(^29^Si,^1^H) = 218 Hz. ^d^ Contains about 9 % of **6** due to non-selectivity of the reaction yet all ***p*-q(*t*Bu)_2_** was consumed.


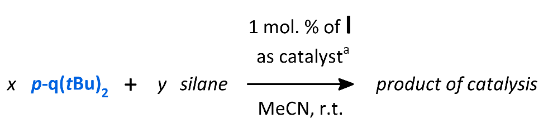


| *x* | *y* | *silane* | *product of catalysis* | | | δ(^29^Si) of *product* [ppm] | | Reaction time [days] | Conversion^b^ [%] |
| --- | --- | --- | --- | --- | --- | --- | --- | --- | --- |
| 1 | 1 | Ph_3_**SiH** | 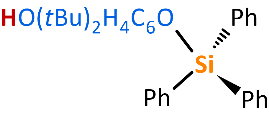 | **3^Ph^** | −14.6 | | 2 | | >99 |
| 2 | 1 | 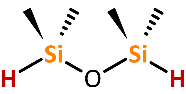 | 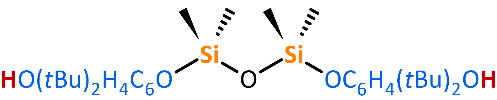 | **4** | −13.9 | | 0.5 | | >99 |
| 4 | 1 | 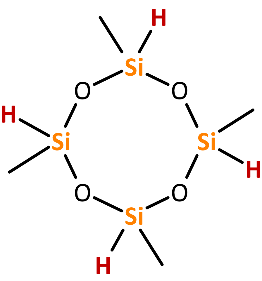 | 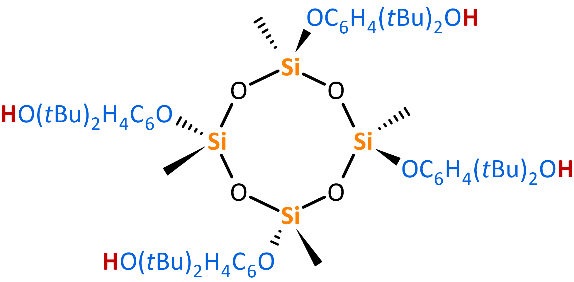 | **5** | −60.4 | | 2 | | >99 |
| 2 | 1 | Ph_2_**SiH_2_** | 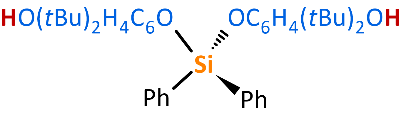  The product is already known in literature[^[7]^](#_ENREF_3) | **6** | −38.0 | | 1 | | >99 |
| 1 | 1 | 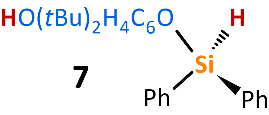 | 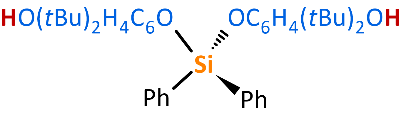 | **6** | −38.0 | | 1 | | >99 |
| 1 | 1 | Ph_2_**SiH_2_** | 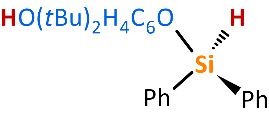 | **7** | −14.1^c^ | | 1 | | >99^d^ |
| 3 | 1 | Ph**SiH_3_** | 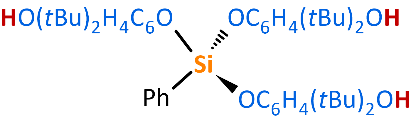 | **8** | −69.2 | | 5 | | >99 |
| 4 | 1 | **SiH_4_** | 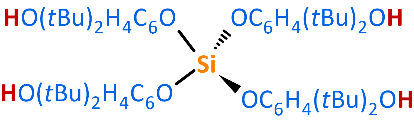  The product is already known in literature[^[8]^](#_ENREF_4) | **9** | −98.6 | | 3 | | >99 |

Considered redox changes

Half-reactions on matter of a Si−H bond activation:

R_4−_*_n_*SiH*_n_* 🡪 (R_4−_*_n_*SiH*_n_*_−1_)^+^ + 2 *e*^–^ + H^+^ (eq. 1)

2 Te^II(+)^ (**I**) + 2 *e*^−^ 🡪 Te^I^−Te^I^ (**II**) (eq. 2)

Half-reactions of re-oxidation of generated ditelluride **II** by quinone:

Te^I^−Te^I^ (**II**) − 2 *e*^−^ 🡪 2 x Te^II^ (**IIIb’**) (eq. 3)

***p*-q(*t*Bu)_2_** + 2 *e*^−^ 🡪 (***p*-q(*t*Bu)_2_**)^2−^ (eq. 4)

Summary reaction for formation of product of catalysis:

**(*p*-q(*t*Bu)_2_)^2−^** + (R_4−_*_n_*SiH*_n_*_−1_)^+^ + H^+^ 🡪 (R_4−_*_n_*SiH*_n_*_−1_)OC_6_H_2_(*t*Bu)_2_OH (eq. 5)

Catalytic synthesis of compound **3^Ph^** using **I** as catalyst


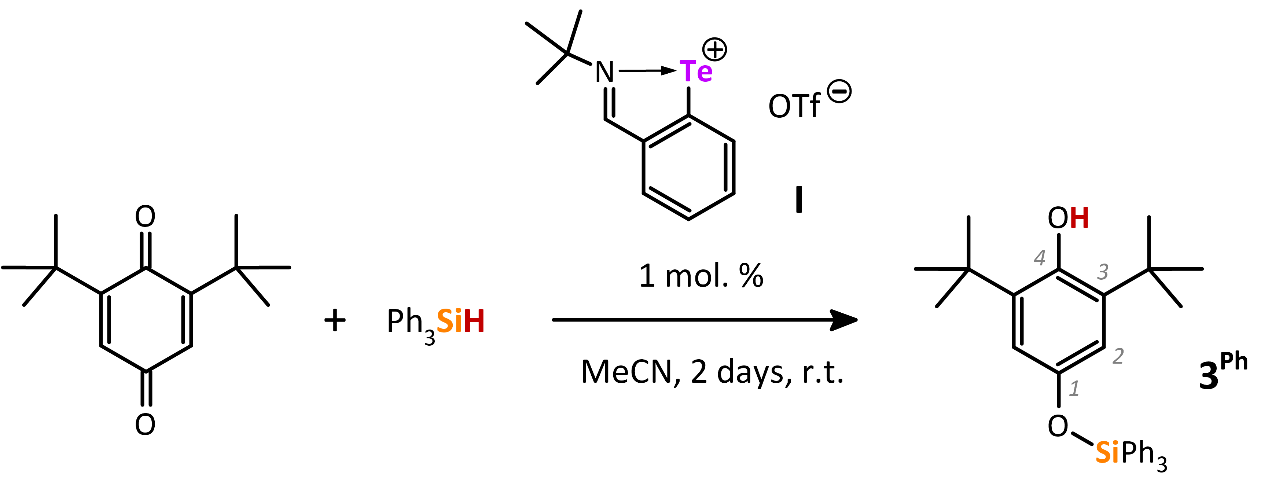


Scheme S2

10.0 mg (0.0229 mmol, 1 mol. %) of **I** (catalyst) and 504 mg (2.29 mmol) of 2,6-di-*tert*-butyl-1,4-benzoquinone (***p*-q(*t*Bu)_2_**) was loaded into a Schlenk tube and dissolved in 25 mL of dry and degassed acetonitrile under an argon atmosphere (**Scheme S2**). Subsequently, the obtained yellow solution was transferred by cannula to another Schlenk tube containing 596 mg (2.29 mmol) of triphenylsilane (a crystalline solid) under vigorous stirring. The color of the obtained solution turned to orange by generation of low steady-state concentration of blood red **II**. The reaction mixture was stirred for 2 days until the color faded. The resulting light orange solution was evaporated at low pressure and dried *in vacuo* to give a yellowish oil with a small amount of orange precipitate from which the product **3^Ph^** was extracted by hexane (20 mL). The obtained hexane solution of **3^Ph^** was concentrated at reduced pressure and upon storing at −30 °C the product **3^Ph^** was obtained as yellowish (almost colorless) polycrystals overnight (m.p. = 85 – 87 °C). Isolated yield of **3^Ph^** was 979 mg (89 %). Conversion of the reaction was >99 % based on NMR analysis of the crude oil in C_6_D_6_.

*NMR data for isolated* ***3^Ph^*** *in C_6_D_6_:*

**^1^H NMR** (500.20 MHz, C_6_D_6_) δ (ppm): 1.24 [18H, s, 2x (C*H*_3_)_3_C-]; 4.54 [1H, s, O*H*]; 6.99 [2H, s, 2x Ar(C2)*H*]; 7.12–7.17 [9H, m, *p*-Ph_3_Si- and *m*-Ph_3_Si-]; 7.82 [6H, dd, *o*-Ph_3_Si-]. **^13^C{^1^H} NMR** (125.78 MHz, C_6_D_6_) δ (ppm): 30.6 [s, 2x (*C*H_3_)_3_C-]; 34.8 [s, qC, (CH_3_)_3_*C*-]; 117.5 [s, 2x Ar-(*C2*)H]; 128.6 [s, *m*-*C*H, Ph_3_Si-]; 131.0 [s, *p*-*C*H, Ph_3_Si-]; 134.9 [qC, *ipso*-*C*, Ph_3_Si-, ^1^*J*(^29^Si,^13^C) = 81.6 Hz]; 136.4 [s, *o*-*C*H, Ph_3_Si-]; 137.4 [s, qC, 2x Ar-(*C3*)]; 148.8 [s, qC, Ar-(*C1 or 4*)]; 149.0 [s, qC, Ar-(*C1 or 4*)]. **^29^Si{^1^H} NMR** (99.38 MHz, C_6_D_6_) δ: −14.6 ppm


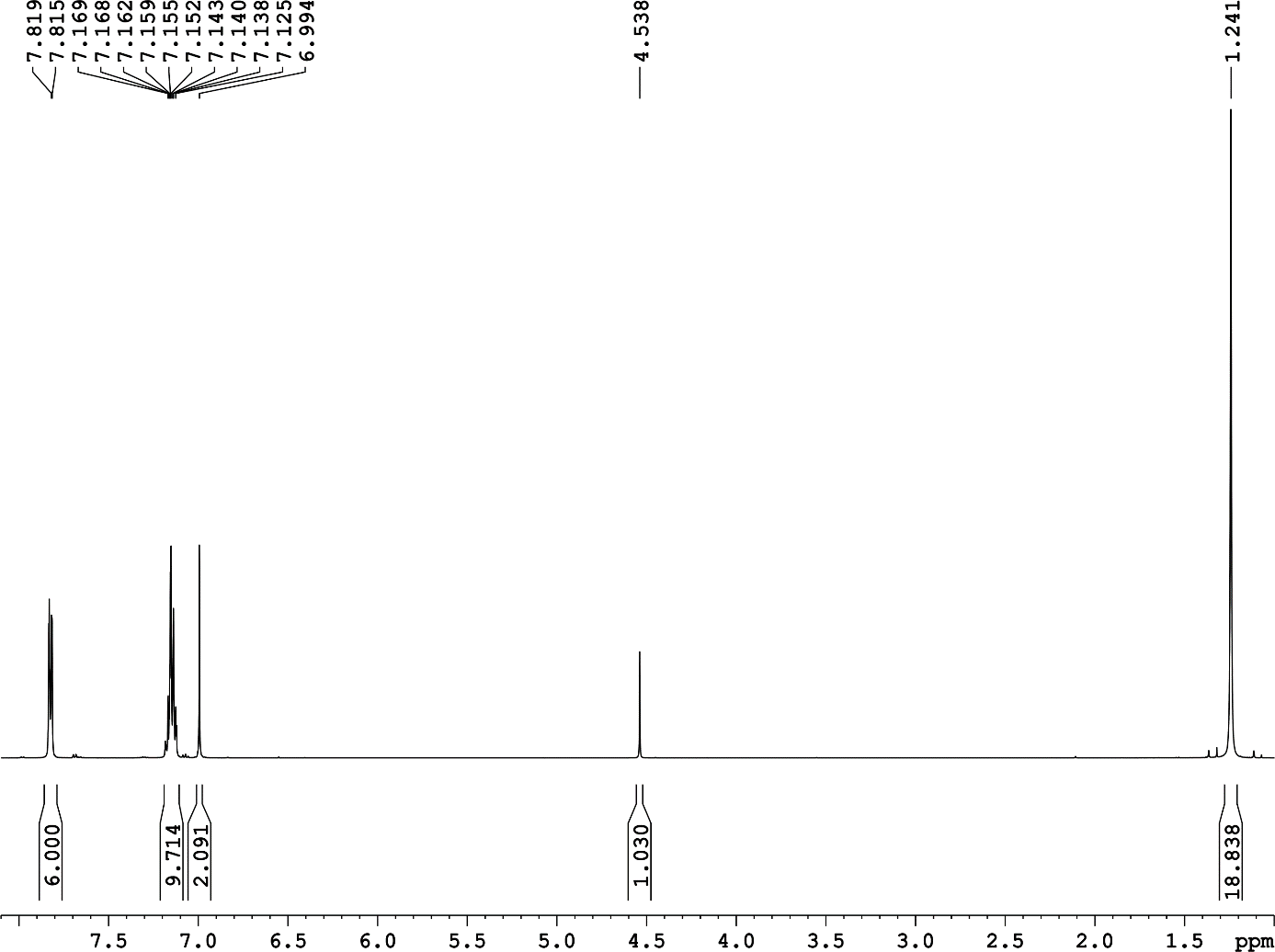

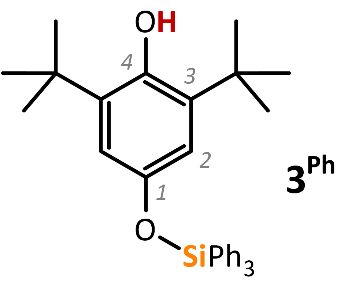


Figure S1: ^1^H NMR spectrum of isolated 3^Ph^ in C_6_D_6_ (500.20 MHz, 295 K).


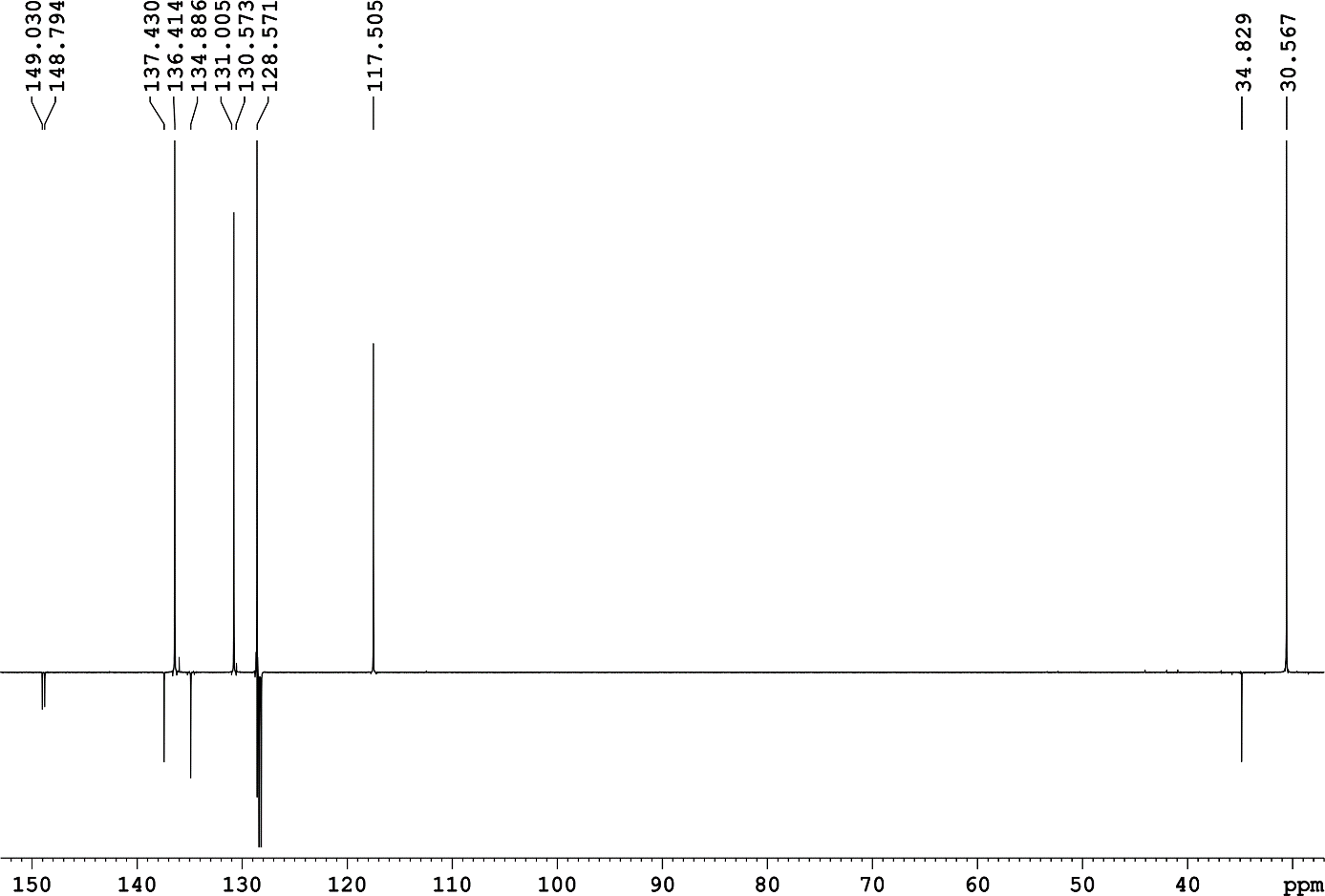

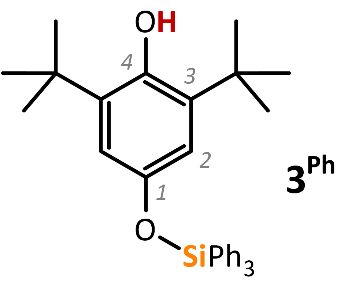


Figure S2: ^13^C{^1^H} APT NMR spectrum of isolated 3^Ph^ in C_6_D_6_ (125.78 MHz, 295 K).


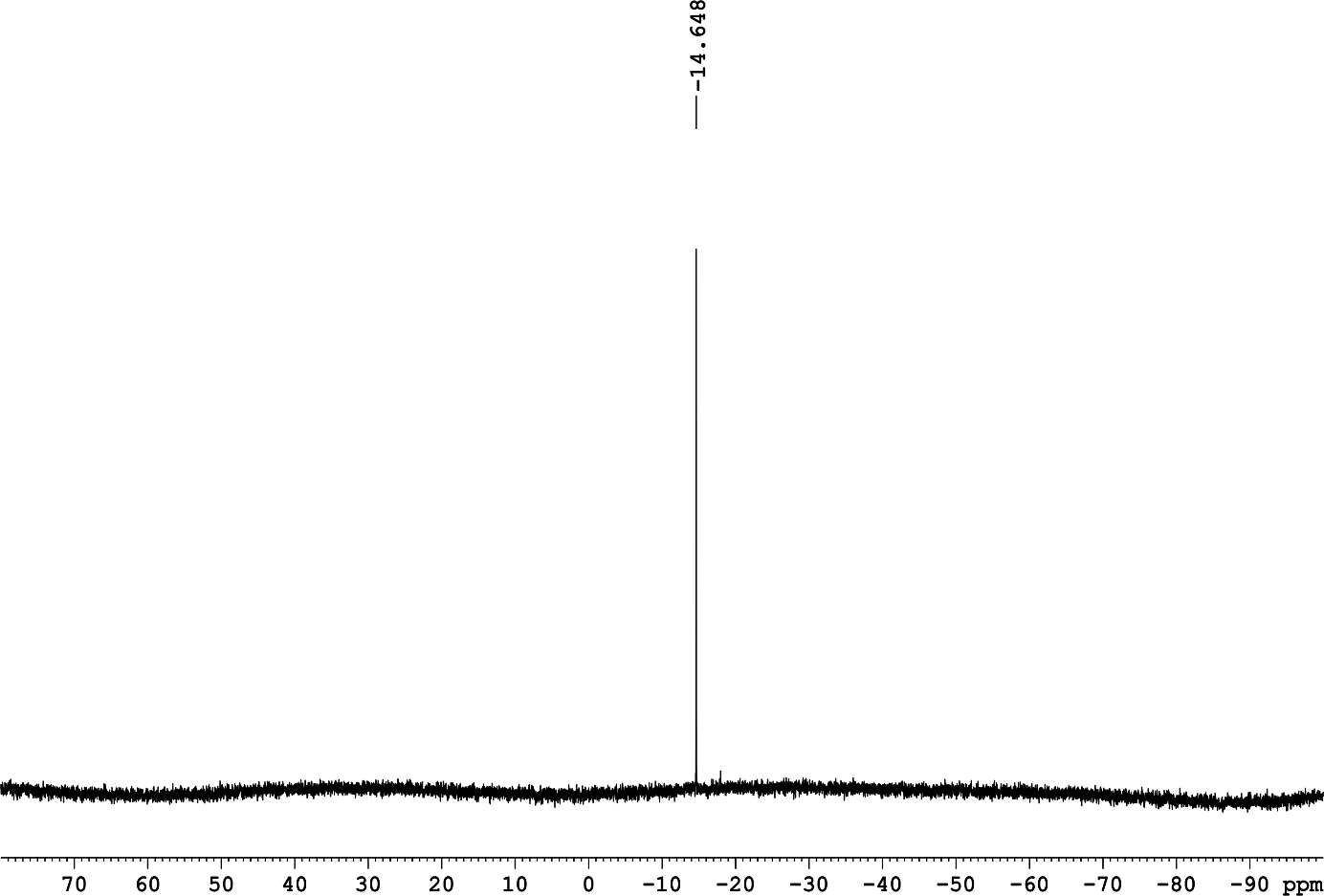

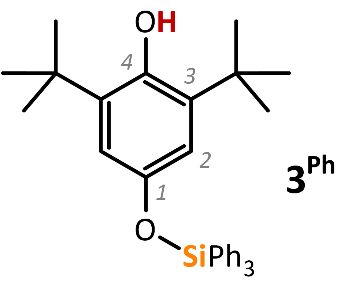


Figure S3: ^29^Si{^1^H} NMR spectrum of isolated 3^Ph^ in C_6_D_6_ (99.37 MHz, 295 K).

Catalytic synthesis of compound **4** using **I** as catalyst


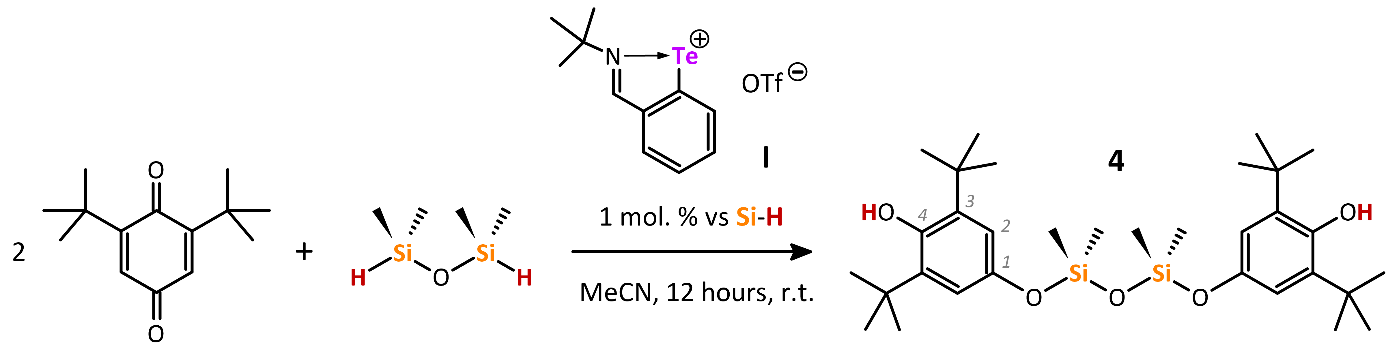


Scheme S3

60.0 mg (0.137 mmol, 1 mol. % *vs* Si-H) of **I** (catalyst) and 3.03 g (13.7 mmol) of 2,6-di-*tert*-butyl-1,4-benzoquinone (***p*-q(*t*Bu)_2_**) was loaded into a Schlenk tube and dissolved in 100 mL of dry and degassed acetonitrile under argon atmosphere. Subsequently, 1.25 mL (7.07 mmol) of neat 1,1,3,3-tetramethyldisiloxane was added to this yellow solution under vigorous stirring (**Scheme S3**). After addition of the silane, the color of the solution slowly turned to orange due to the generation of low steady-state concentration of blood red **II**. After about 3 hours of stirring, a white precipitate started to appear. The reaction mixture was stirred for 12 hours in total. The resulting suspension consisting of a light orange solution and a white powder was filtered. The obtained ivory white powder of crude product **4** was washed by cold acetonitrile (5 mL), dried *in vacuo* and recrystallized from a minimal amount of hot toluene. By slow cooling of the obtained solution to r.t., colorless single-crystals of **4** (3.71 g, 94 %) were obtained (m.p. = 139 – 141 °C). Conversion of the reaction was >99 % based on NMR analysis of both the white powder of precipitated **4** and of the evaporated orange solution in C_6_D_6_ showing no trace of unreacted ***p*-q(*t*Bu)_2_**. *Note:* A solubility curve for **4** in toluene is very steep with temperature. In hexane, the solubility of **4** is very limited.

*NMR data for isolated* ***4*** *in C_6_D_6_:*

**^1^H NMR** (500.20 MHz, C_6_D_6_) δ (ppm): 0.31 [12H, s, 4x C*H*_3_-Si]; 1.35 [36H, s, 4x (C*H*_3_)_3_C-]; 4.58 [2H, s, 2x O*H*]; 7.05 [4H, s, 4x Ar(C2)*H*]. **^13^C{^1^H} NMR** (125.78 MHz, C_6_D_6_) δ (ppm): 0.0 [s, *C*H_3_-Si, ^1^*J*(^29^Si,^13^C) = 75.0 Hz]; 30.7 [s, (*C*H_3_)_3_C-]; 34.9 [s, qC, (CH_3_)_3_*C*-]; 117.1 [s, Ar-(*C2*)H]; 137.7 [s, qC, Ar-(*C3*)]; 148.2 [s, qC, Ar-(*C1 or 4*)]; 149.2 [s, qC, Ar-(*C1 or 4*)]. **^29^Si{^1^H} NMR** (99.38 MHz, C_6_D_6_) δ: −13.9 ppm.


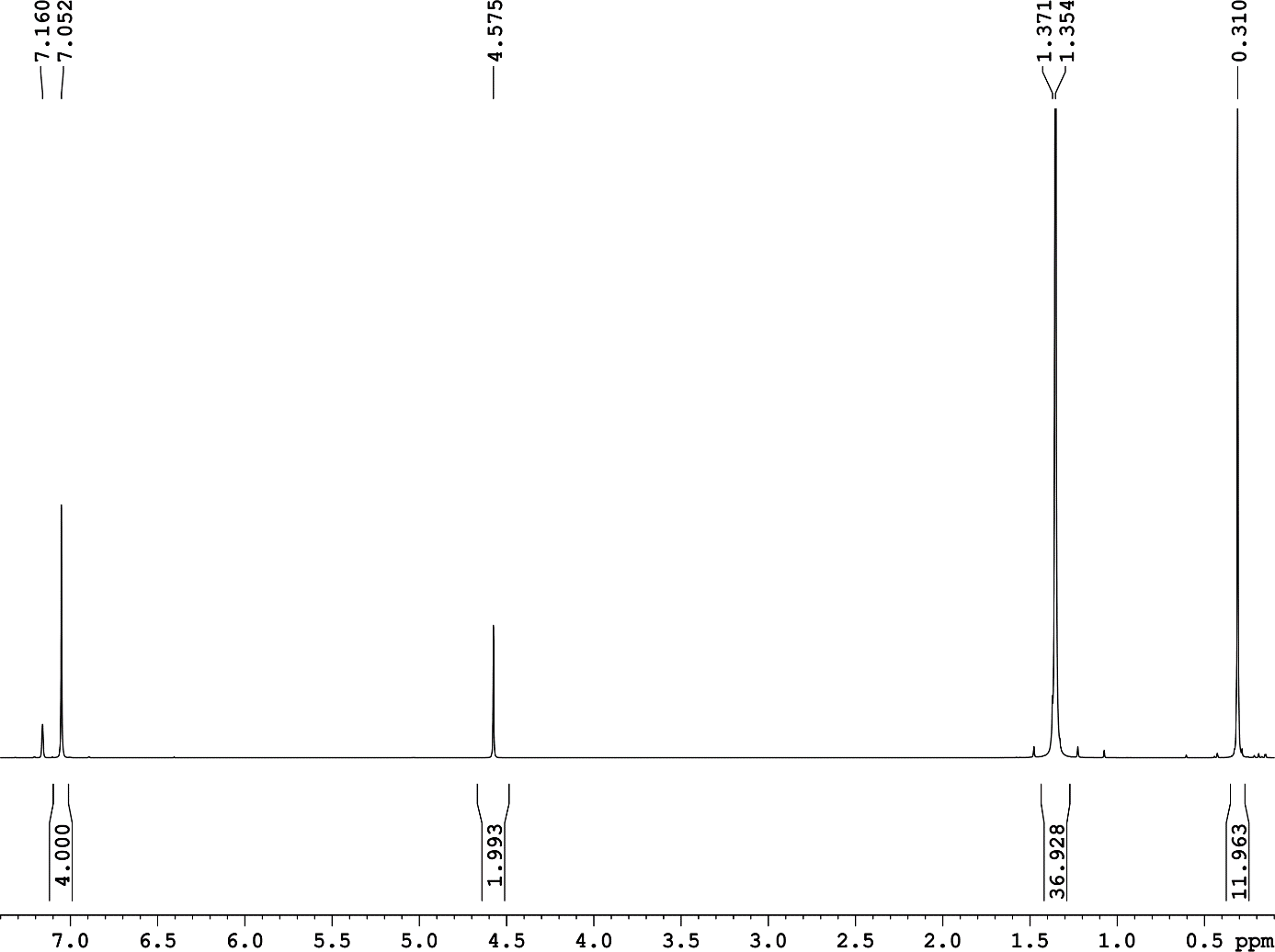

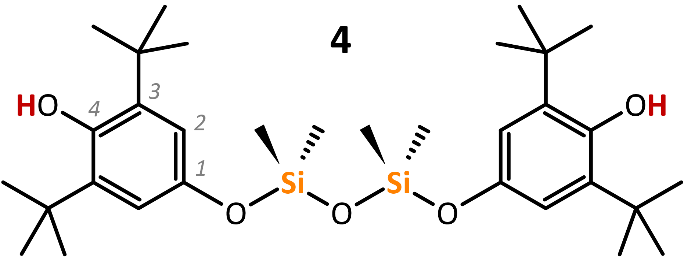


Figure S4: ^1^H NMR spectrum of isolated 4 in C_6_D_6_ (500.20 MHz, 295 K).


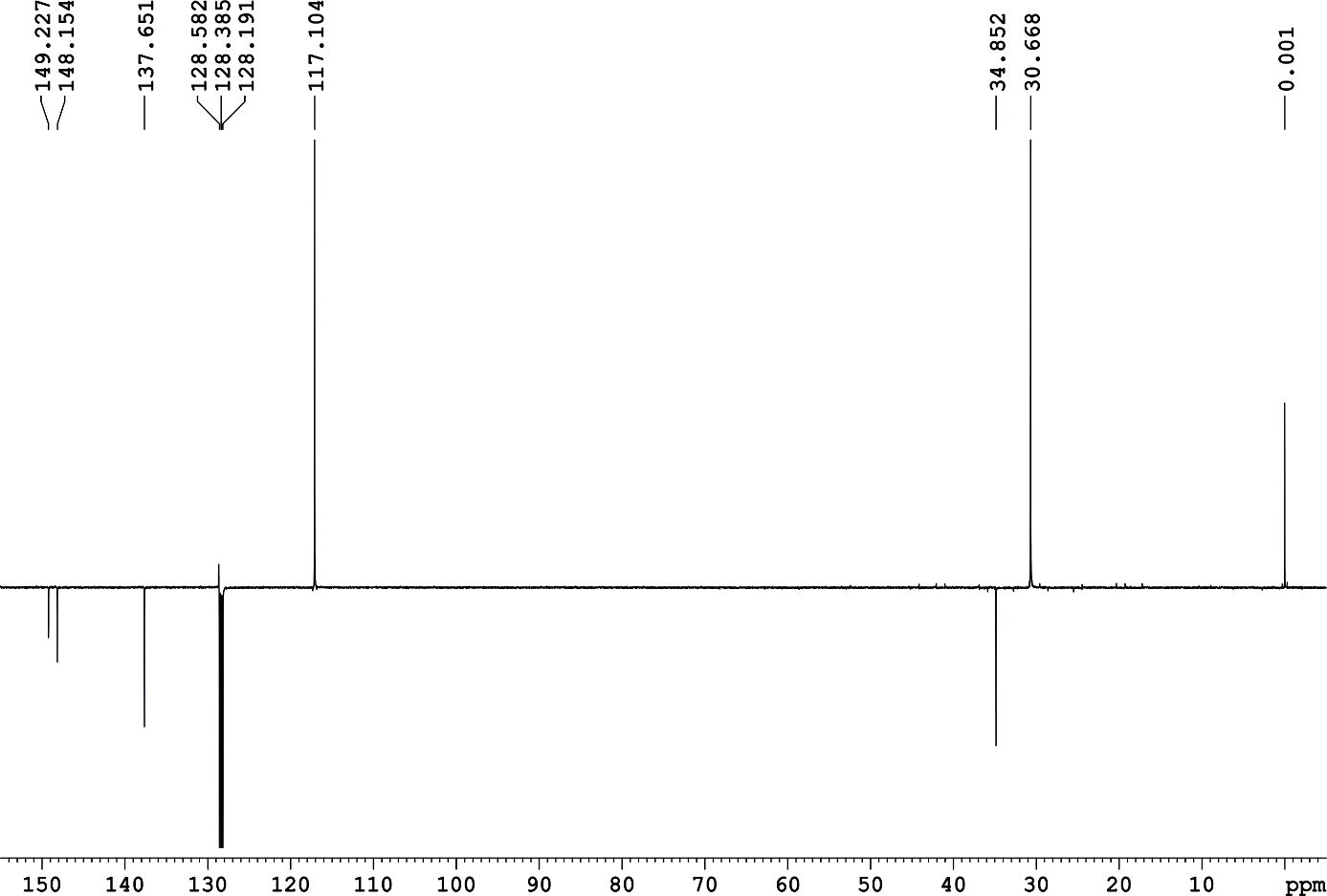

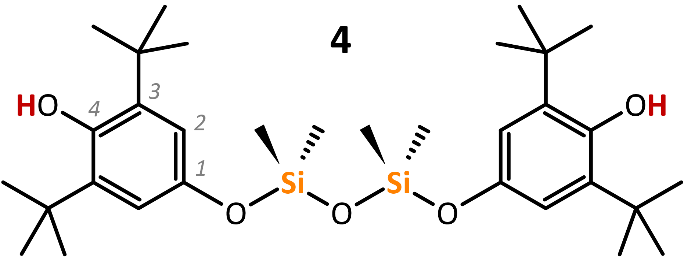


Figure S5: ^13^C{^1^H} APT NMR spectrum of isolated 4 in C_6_D_6_ (125.78 MHz, 295 K).


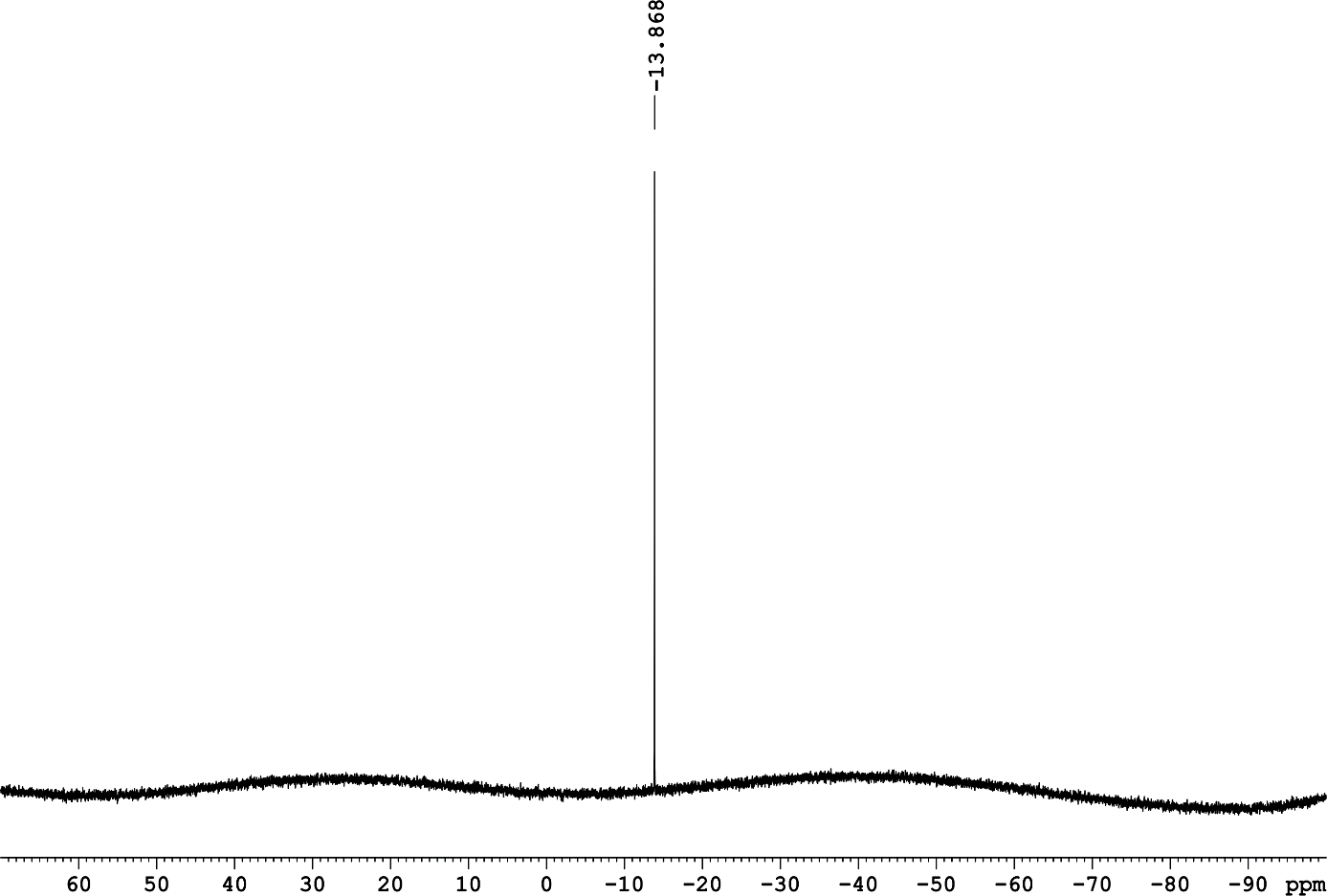

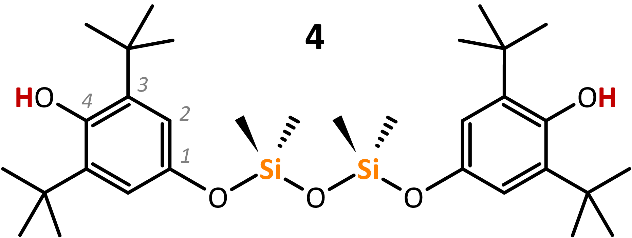


Figure S6: ^29^Si{^1^H} NMR spectrum of isolated 4 in C_6_D_6_ (99.37 MHz, 295 K).

Catalytic synthesis of compound **5** using **I** as catalyst


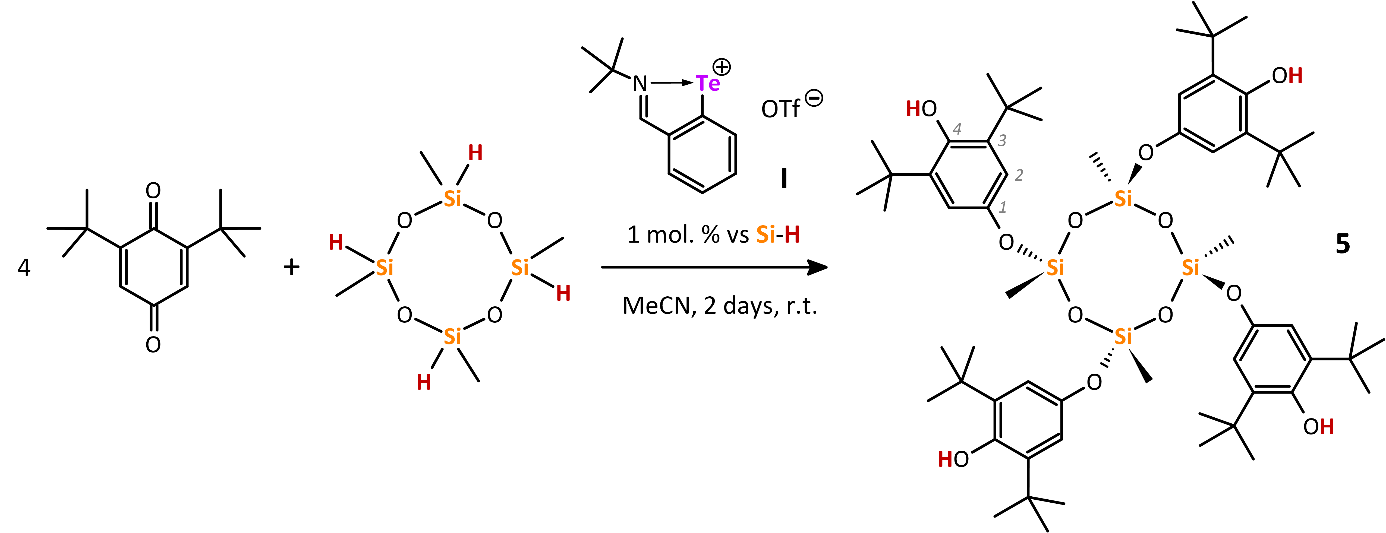


Scheme S4

73.0 mg (0.167 mmol, 1 mol. % *vs* Si-H) of **I** (catalyst) and 3.68 g (16.7 mmol) of 2,6-di-*tert*-butyl-1,4-benzoquinone (***p*-q(*t*Bu)_2_**) was loaded into a Schlenk tube and dissolved in 100 mL of dry and degassed acetonitrile under argon atmosphere. Subsequently, 1.02 mL (4.18 mmol) of neat 2,4,6,8-tetramethylcyclotetrasiloxane was added to this yellow solution under vigorous stirring (**Scheme S4**). After the addition of the silane, the color of the solution slowly turned to orange due to the generation of low steady-state concentration of blood red **II**. After 2 days of stirring, filtration of the orange suspension, gave an ivory white powder of crude product **5**, which was washed by cold acetonitrile (5 mL; *Note:* **5** is slightly soluble in acetonitrile!), dried *in vacuo* and recrystallized from a minimal amount of boiling toluene. By slow cooling of the obtained solution to 6 °C huge colorless single-crystals of **5** (4.31 g, 92 %) were obtained (m.p. = 217 – 219 °C). Conversion of the reaction was >99 % based on NMR analysis of both the ivory white powder of precipitated **5** and of the evaporated orange solution in C_6_D_6_ showing no trace of unreacted ***p*-q(*t*Bu)_2_**.

*NMR data for isolated* ***5*** *in C_6_D_6_:*

**^1^H NMR** (500.20 MHz, C_6_D_6_) δ (ppm): 0.37 [12H, s, 4x C*H*_3_-Si]; 1.34 [72H, s, 4x (C*H*_3_)_3_C-]; 4.58 [4H, s, 4x O*H*]; 7.15 [8H, s, 8x Ar(C2)*H*]. **^13^C{^1^H} NMR** (125.78 MHz, C_6_D_6_) δ (ppm): −4.0 [s, *C*H_3_-Si, ^1^*J*(^29^Si,^13^C) = 103.0 Hz]; 30.6 [s, (*C*H_3_)_3_C-]; 34.9 [s, qC, (CH_3_)_3_*C*-]; 117.1 [s, Ar-(*C2*)H]; 137.7 [s, qC, Ar-(*C3*)]; 147.2 [s, qC, Ar-(*C1 or 4*)]; 149.6 [s, qC, Ar-(*C1 or 4*)]. **^29^Si{^1^H} NMR** (99.38 MHz, C_6_D_6_) δ: −60.4 ppm


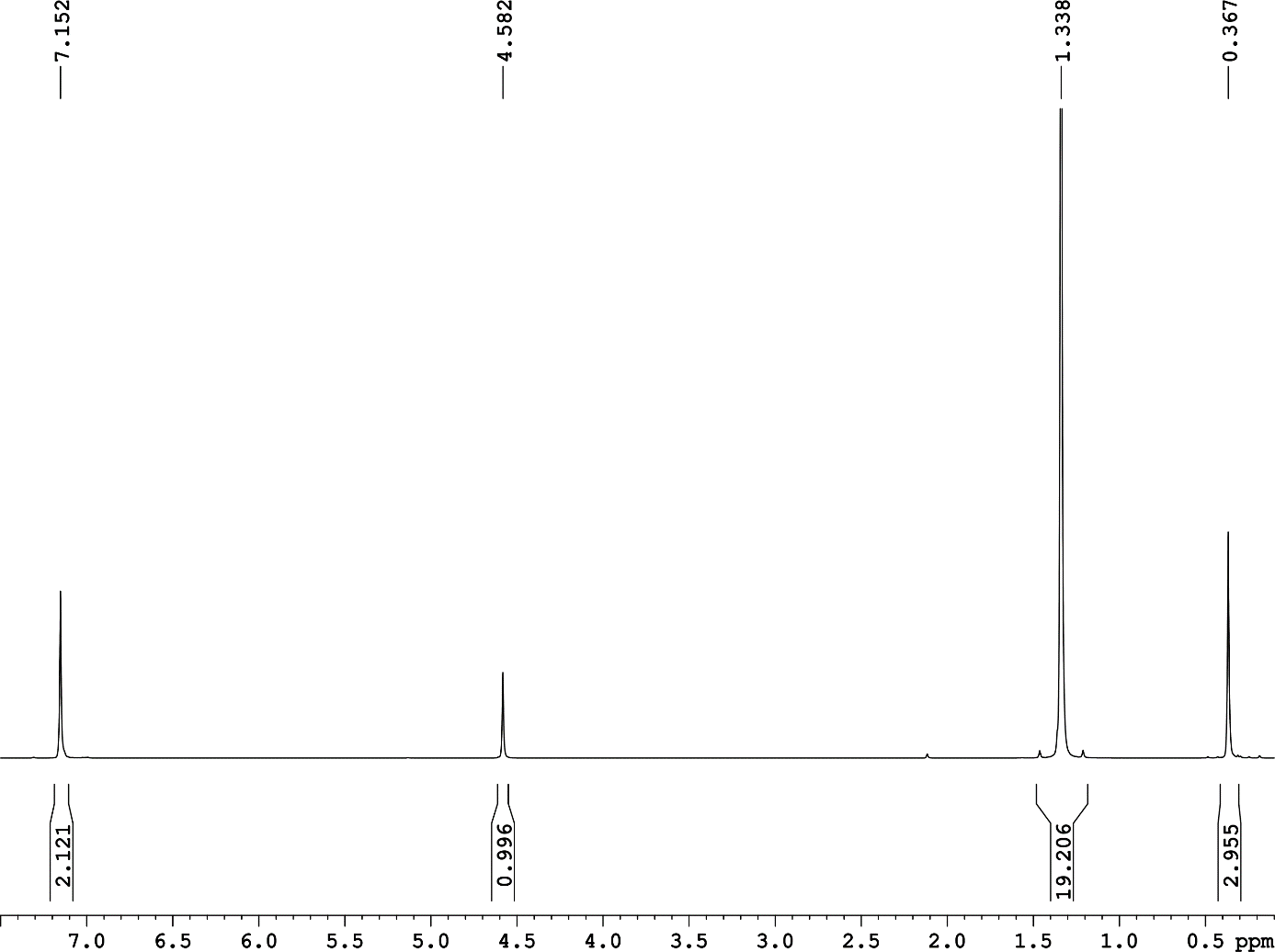

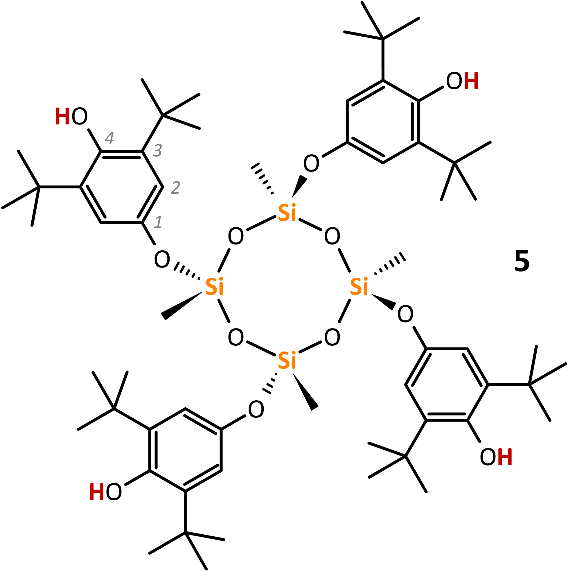


Figure S7: ^1^H NMR spectrum of isolated 5 in C_6_D_6_ (500.20 MHz, 295 K).


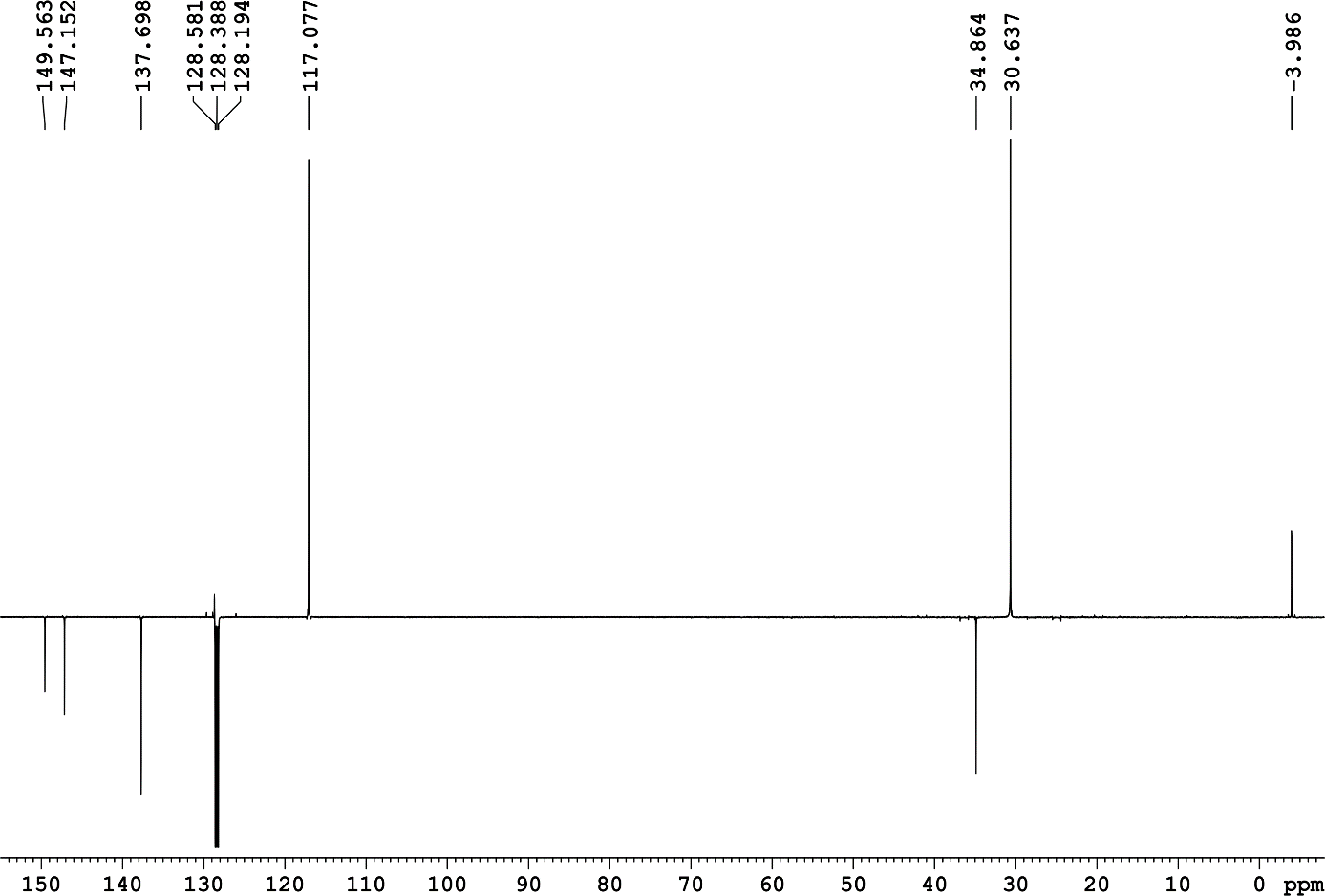

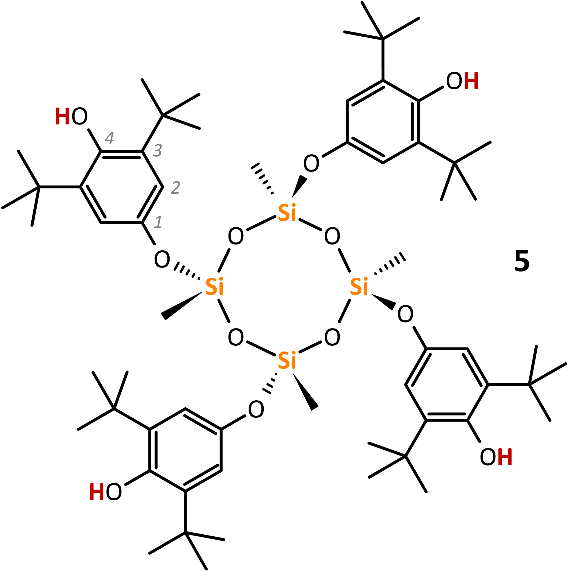


Figure S8: ^13^C{^1^H} APT NMR spectrum of isolated 5 in C_6_D_6_ (125.78 MHz, 295 K).


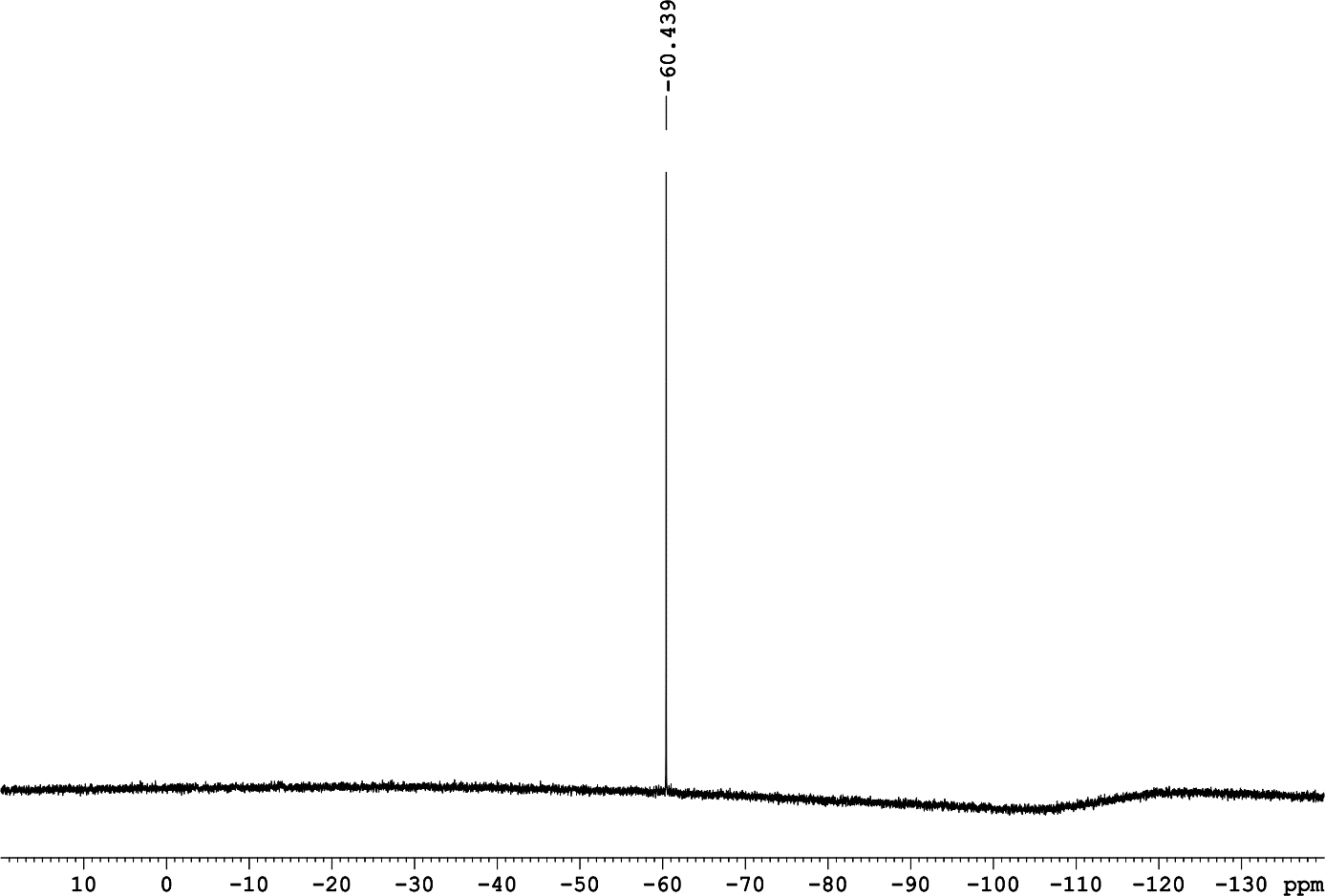

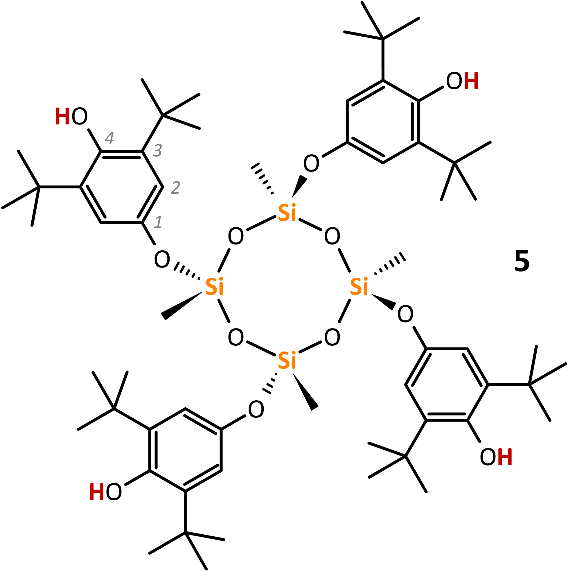


Figure S9: ^29^Si{^1^H} NMR spectrum of isolated 5 in C_6_D_6_ (99.37 MHz, 295 K).

Catalytic synthesis of compound **6** using **I** as catalyst


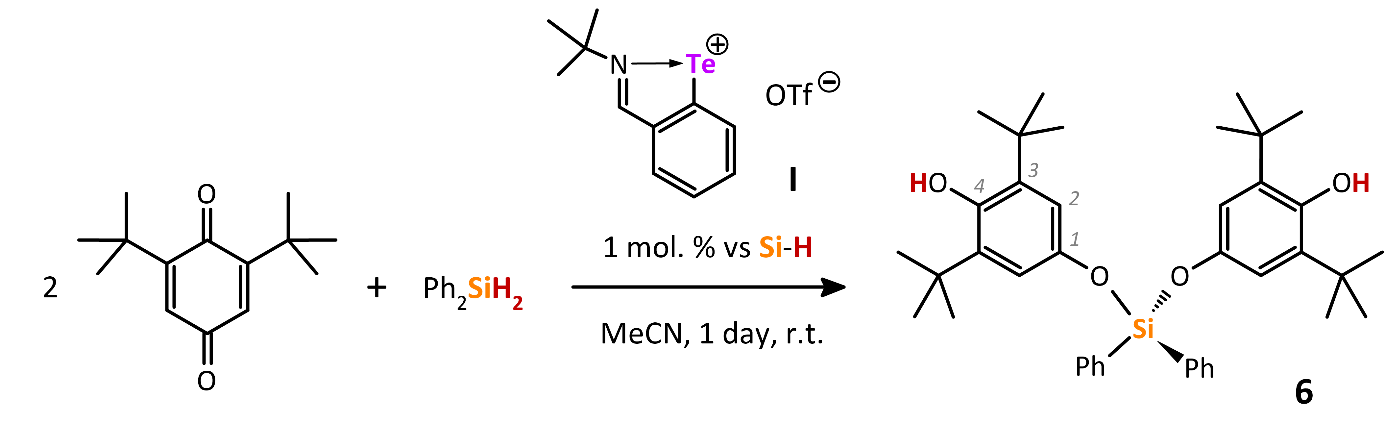


Scheme S5

10.0 mg (0.0229 mmol, 1 mol. % *vs* Si-H) of **I** (catalyst) and 504 mg (2.29 mmol) of 2,6-di-*tert*-butyl-1,4-benzoquinone (***p*-q(*t*Bu)_2_**) was loaded into a Schlenk tube and dissolved in 25 mL of dry and degassed acetonitrile under argon atmosphere. Subsequently, 214 μL (1.17 mmol) of neat diphenylsilane Ph_2_SiH_2_ was added to this yellow solution under vigorous stirring (**Scheme S5**). After the addition of the silane, the color of the solution slowly turned to orange by the generation of low steady-state concentration of blood red **II**. After a day of stirring, the resulting suspension of light orange solution and white powder was filtered. The obtained ivory white powder of crude product **6** was washed with acetonitrile (5 mL), dried *in vacuo* and recrystallized from a minimal amount of boiling toluene. By slow cooling of the obtained solution to 6 °C, colorless single-crystals of **6** (622 mg, 87 %) were obtained (m.p. = 172 – 173 °C, reported 171 – 172 °C^[7]^). Conversion of the reaction was >99 % based on NMR analysis of both the white powder of precipitated **6** and of the evaporated orange solution in C_6_D_6_ showing no trace of unreacted ***p*-q(*t*Bu)_2_**.

*NMR data for isolated* ***6*** *in C_6_D_6_:*

**^1^H NMR** (500.20 MHz, C_6_D_6_) δ (ppm): 1.26 [36H, s, 4x (C*H*_3_)_3_C-]; 4.54 [2H, s, 2x O*H*]; 7.11 [4H, s, 4x Ar(C2)*H*]; 7.13–7.17 [6H, m, *p*-Ph_2_Si and *m*-Ph_2_Si]; 8.00 [4H, m, *o*-Ph_2_Si]. **^13^C{^1^H} NMR** (125.78 MHz, C_6_D_6_) δ (ppm): 30.6 [s, 4x (*C*H_3_)_3_C-]; 34.9 [s, qC, 4x (CH_3_)_3_*C*-]; 117.1 [s, 4x Ar-(*C2*)H]; 128.6 [s, *m*-*C*H, Ph_2_Si]; 131.3 [s, *p*-*C*H, Ph_2_Si]; 133.3 [qC, *ipso*-*C*, Ph_2_Si-, ^1^*J*(^29^Si,^13^C) = 100.1 Hz]; 135.9 [s, *o*-*C*H, Ph_2_Si]; 137.5 [s, qC, 4x Ar-(*C3*)]; 148.0 [s, qC, 2x Ar-(*C1 or 4*)]; 149.3 [s, qC, 2x Ar-(*C1 or 4*)]. **^29^Si{^1^H} NMR** (99.38 MHz, C_6_D_6_) δ: −37.9 ppm.


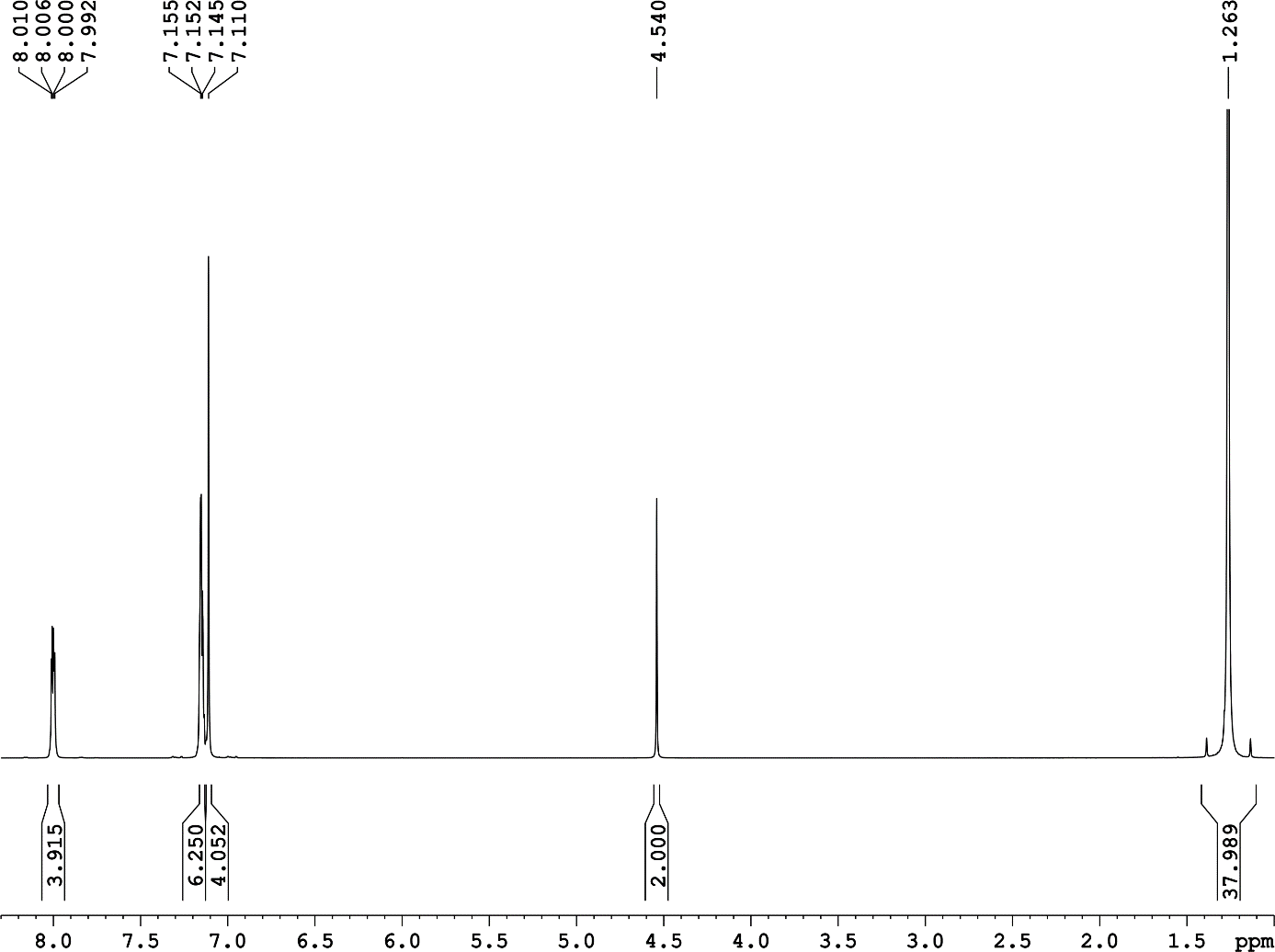

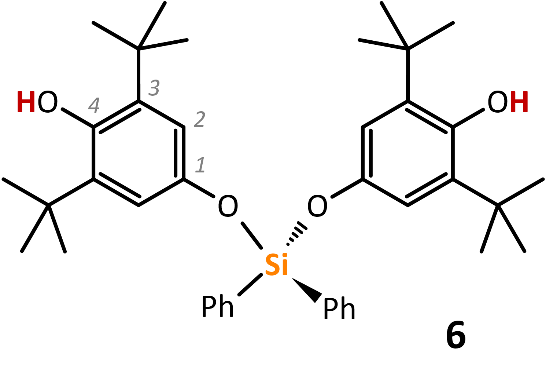


Figure S10: ^1^H NMR spectrum of isolated 6 in C_6_D_6_ (500.20 MHz, 295 K).


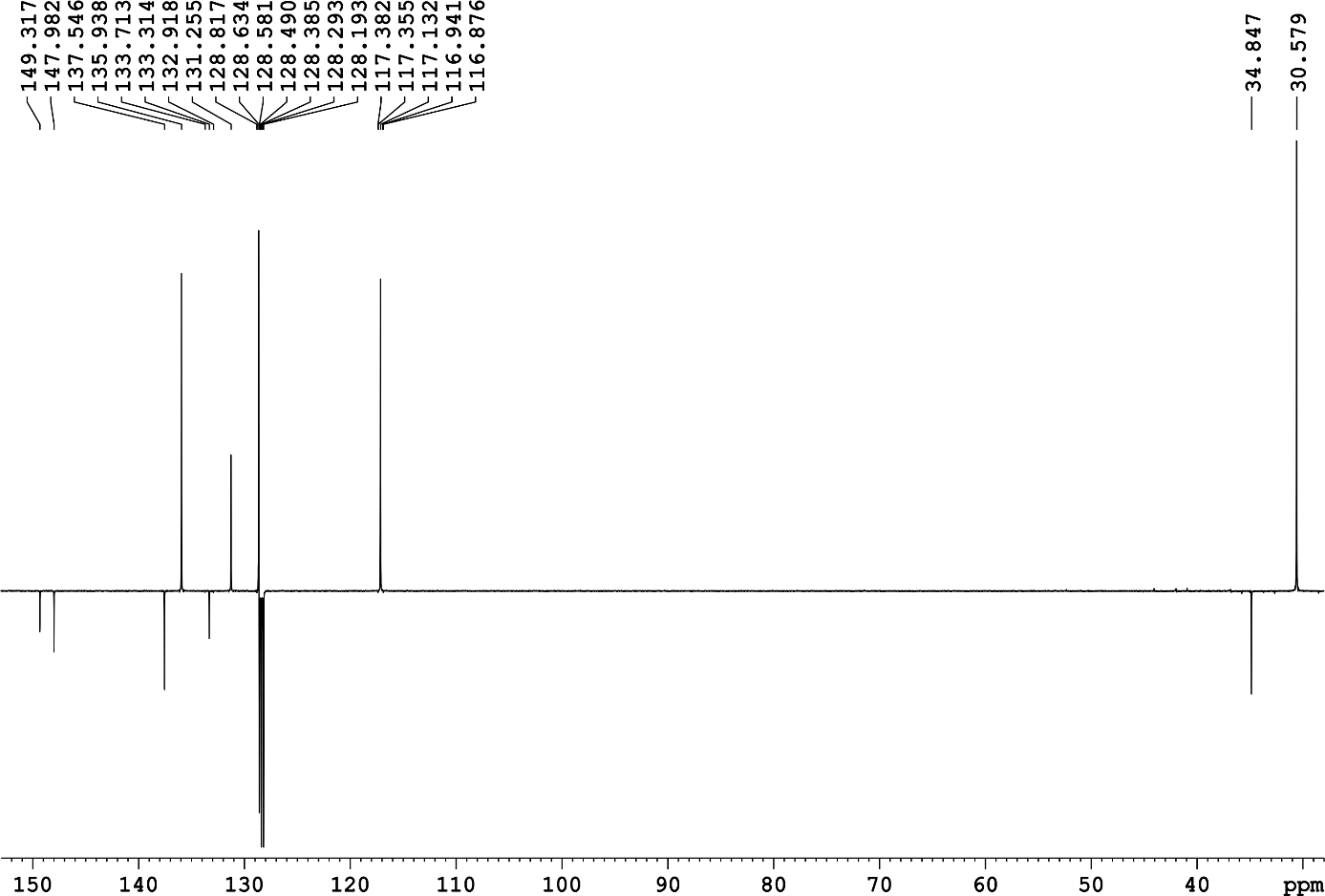

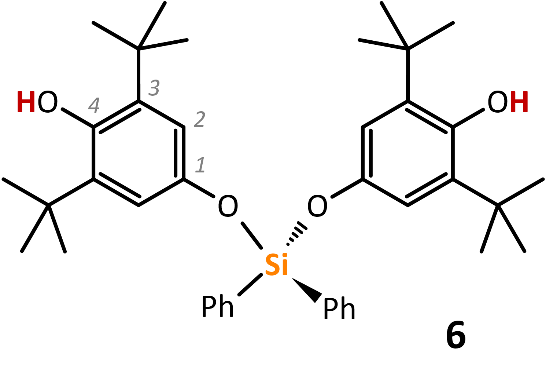


Figure S11: ^13^C{^1^H} APT NMR spectrum of isolated 6 in C_6_D_6_ (125.78 MHz, 295 K).


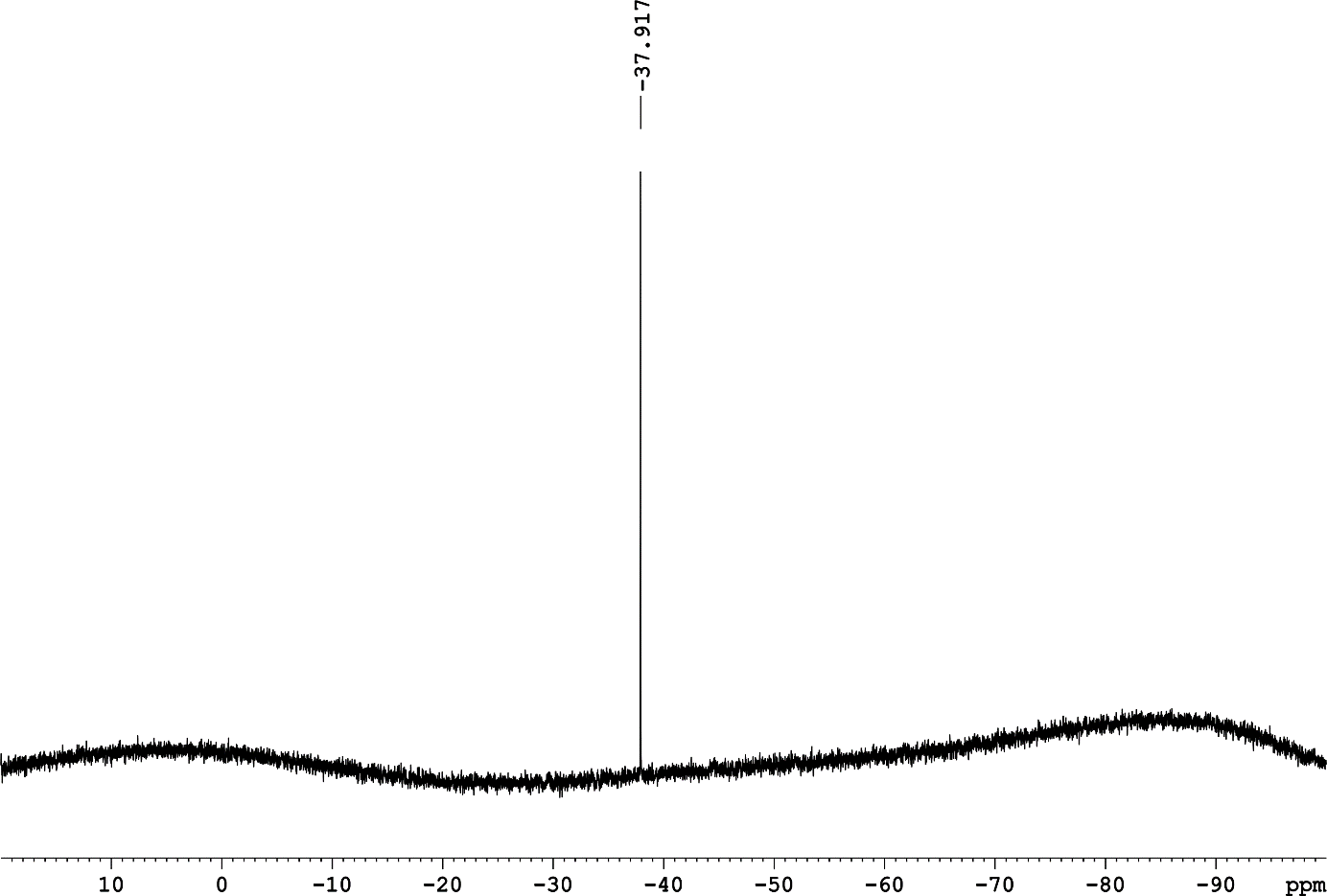

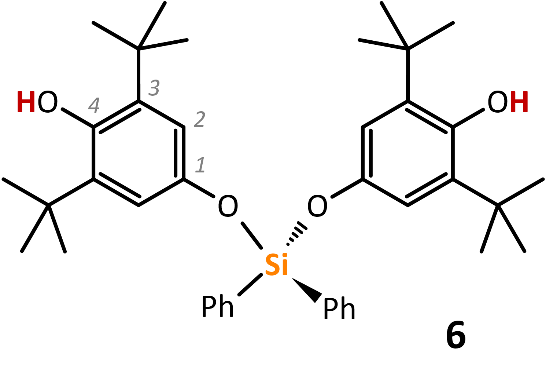


Figure S12: ^29^Si{^1^H} NMR spectrum of isolated 6 in C_6_D_6_ (99.37 MHz, 295 K).

Catalytic synthesis of compound **7** using **I** as catalyst


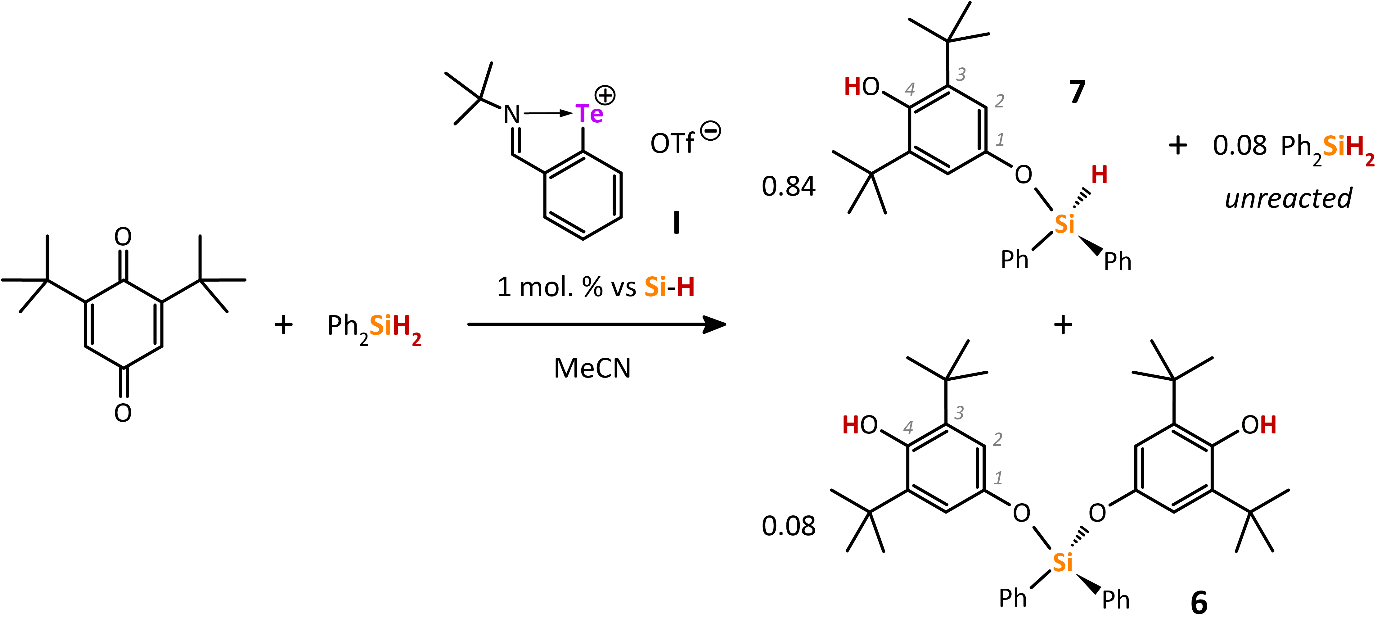


Scheme S6

41.1 mg (0.0941 mmol, 1 mol. %) of **I** (catalyst) and 2.07 g (9.38 mmol) of 2,6-di-*tert*-butyl-1,4-benzoquinone (***p*-q(*t*Bu)_2_**) was loaded into a Schlenk tube and dissolved in 100 mL of dry and degassed acetonitrile under argon atmosphere. Subsequently, 1.74 mL (9.38 mmol) of neat diphenylsilane Ph_2_SiH_2_ was added to this yellow solution under vigorous stirring (**Scheme S6**). After the addition of the silane, the color of the solution slowly turned to orange due to the generation of low steady-state concentration of blood red **II**. After a day of stirring, the resulting light orange solution was evaporated at reduced pressure and properly dried *in vacuo* while forming an orange oil containing **7** and **6** in a relative ratio of 0.91 : 0.09, see **Figure S13** below. The oil was extracted by warm hexane (30 mL) and the resulting yellowish filtrate was concentrated to 15 mL volume. By storing the hexane solution overnight at 6 °C, white polycrystals of the by-product **6** were formed (530 mg). The mother liquor (containing mostly **7**) separated from these crystals (**6**) was concentrated at low pressure to 8 mL. By keeping the obtained solution overnight at 6 °C, white polycrystals of **7** (isolated yield 2.87 g, 76 %; m.p. = 157 – 159 °C) were obtained. Conversion of the reaction is >99 % based on NMR analysis of the reaction mixture (the oil) with no ***p*-q(*t*Bu)_2_** left. *Note:* Lowering the temperature to –10 °C for the first 12 hours of the reaction showed no impact on selectivity.


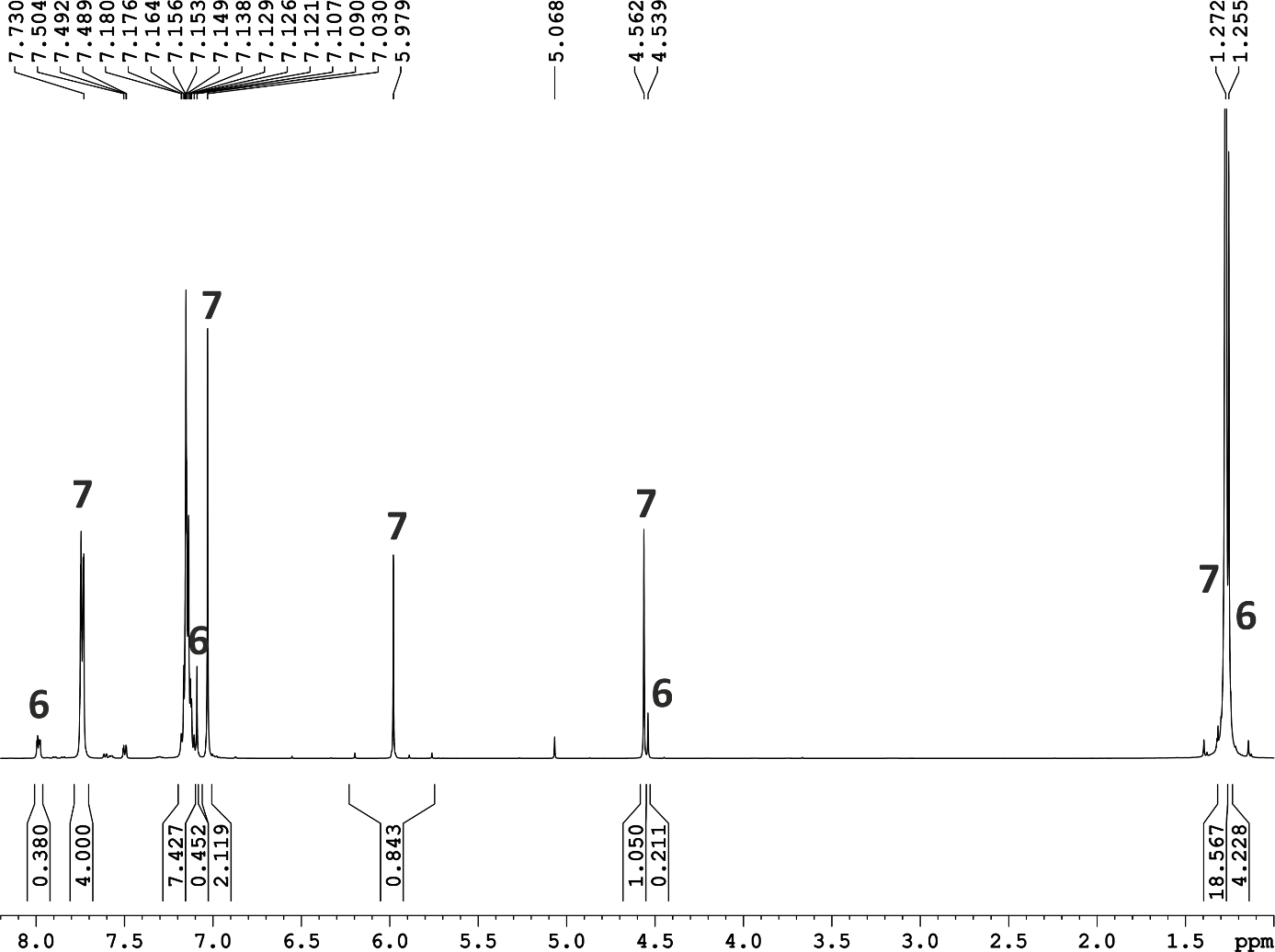


Figure S13: ^1^H NMR spectrum of evaporated reaction mixture (the orange oil described above) containing not only the expected product 7 but also minor of 6 amount (9 % relative to 7) due non-selectivity of the reaction (500.20 MHz, 295 K, C_6_D_6_).


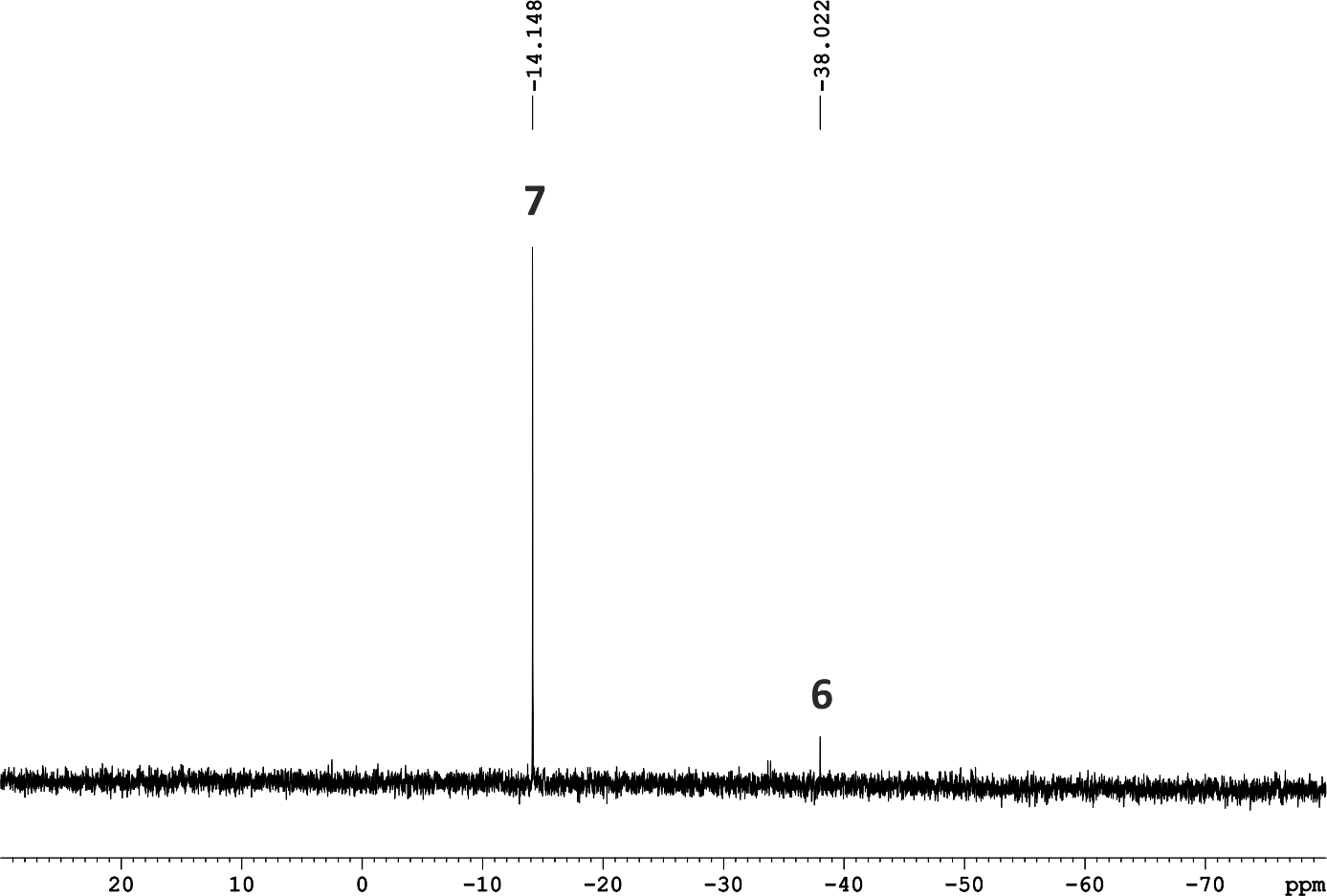


Figure S14: ^29^Si{^1^H} spectrum of evaporated reaction mixture (the orange oil described above) containing not only the expected product 7 but also minor amount (9 %) of 6 due non-selectivity of the reaction (99.37 MHz, 295 K, C_6_D_6_).

*NMR data for isolated* ***7*** *in C_6_D_6_:*

**^1^H NMR** (500.20 MHz, C_6_D_6_) δ (ppm): 1.28 [18H, s, 2x (C*H*_3_)_3_C-]; 4.57 [1H, s, O*H*]; 6.00 [1H, s, Si-*H*, ^1^*J*(^29^Si,^1^H) = 217.7 Hz]; 7.05 [2H, s, 2x Ar(C2)*H*]; 7.13–7.17 [6H, m, 2x *p*-Ph-*H* and 4x *m*-Ph-*H*]; 7.75 [4H, d, 2x *o*-Ph-*H*]. **^13^C{^1^H} NMR** (125.78 MHz, C_6_D_6_) δ (ppm): 30.5 [s, (*C*H_3_)_3_C-]; 34.9 [s, qC, (CH_3_)_3_*C*-]; 116.6 [s, Ar-(*C2*)H]; 128.6 [s, *m*-*C*H, Ph_2_Si-]; 131.1 [s, *p*-*C*H, Ph_2_Si-]; 134.3 [qC, *ipso*-*C*, Ph_3_Si-]; 135.6 [s, *o*-*C*H, Ph_2_Si-]; 137.7 [s, qC, Ar-(*C3*)]; 149.2 [s, qC, Ar-(*C1 or 4*)]; 149.3 [s, qC, Ar-(*C1 or 4*)]. **^29^Si NMR** (99.38 MHz, C_6_D_6_) δ: −14.1 ppm (dq, ^1^*J*(^29^Si,^1^H) = 217.7 Hz).


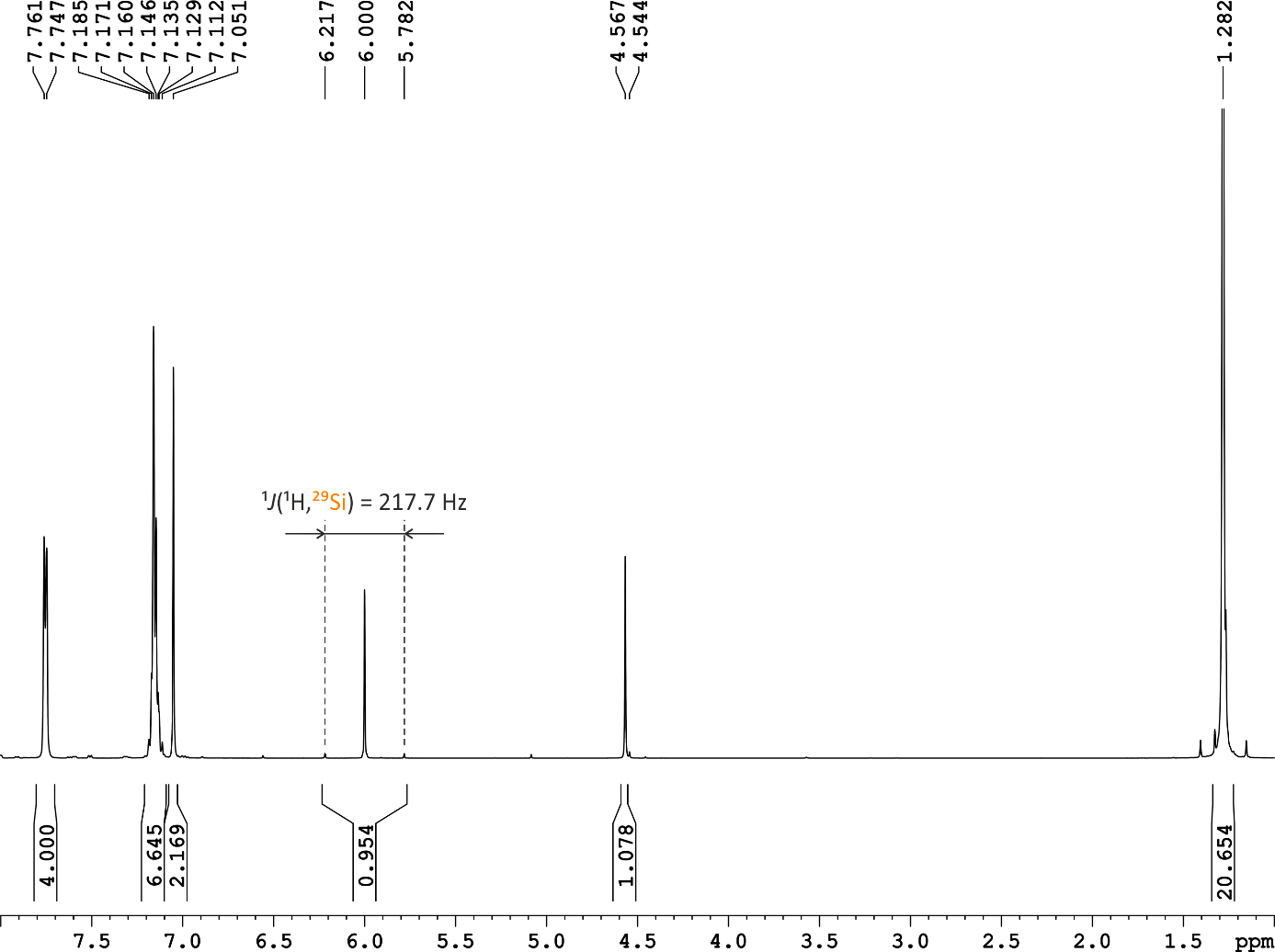

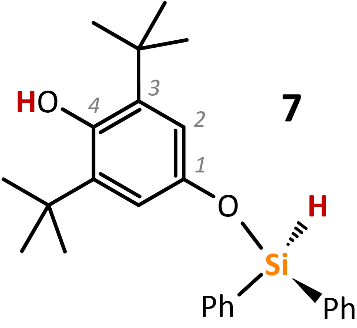


Figure S15: ^1^H NMR spectrum of isolated 7 in C_6_D_6_ (500.20 MHz, 295 K).


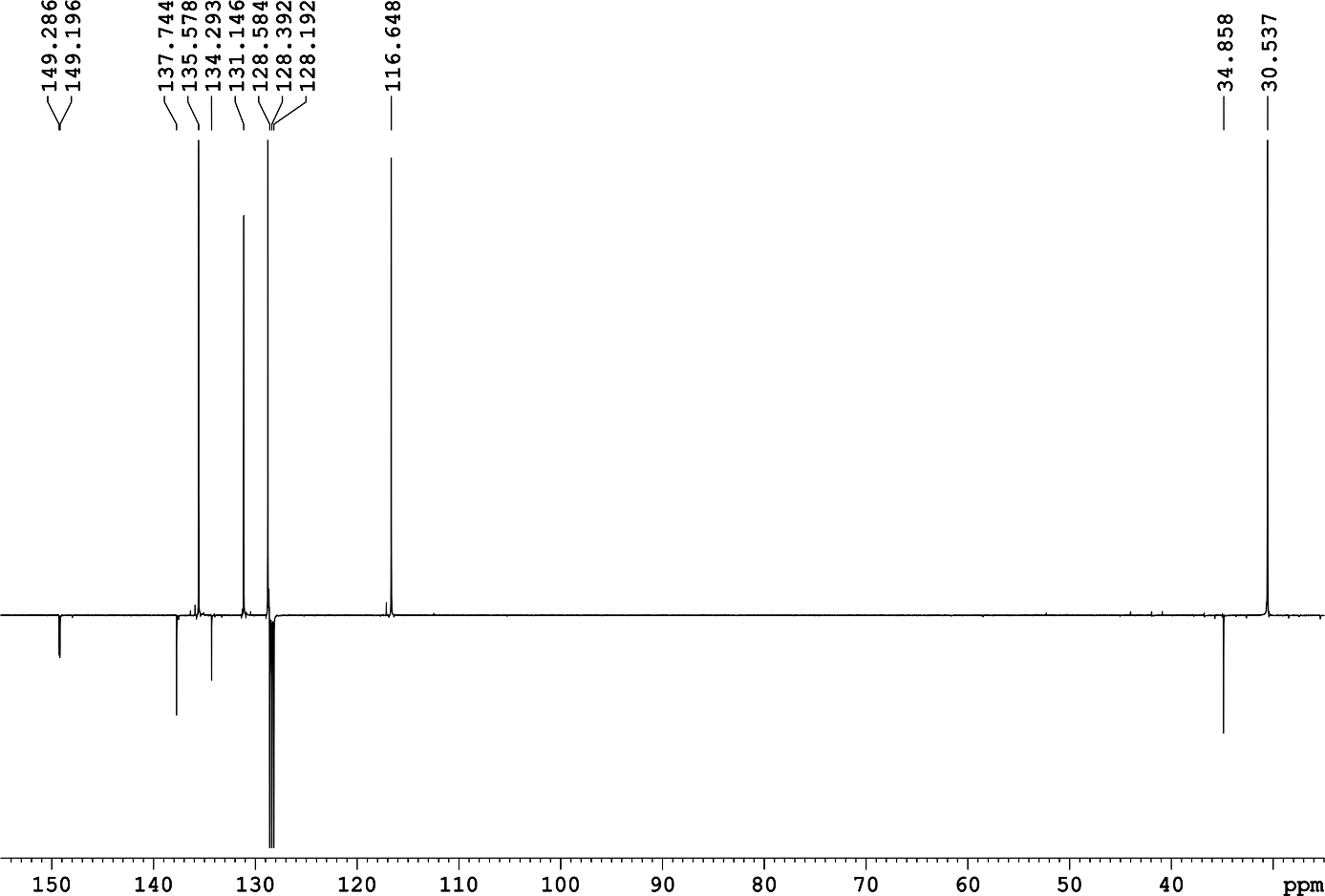

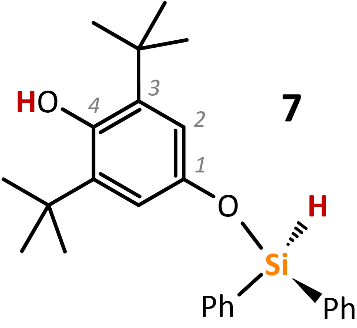


Figure S16: ^13^C{^1^H} APT NMR spectrum of isolated 7 in C_6_D_6_ (125.78 MHz, 295 K).


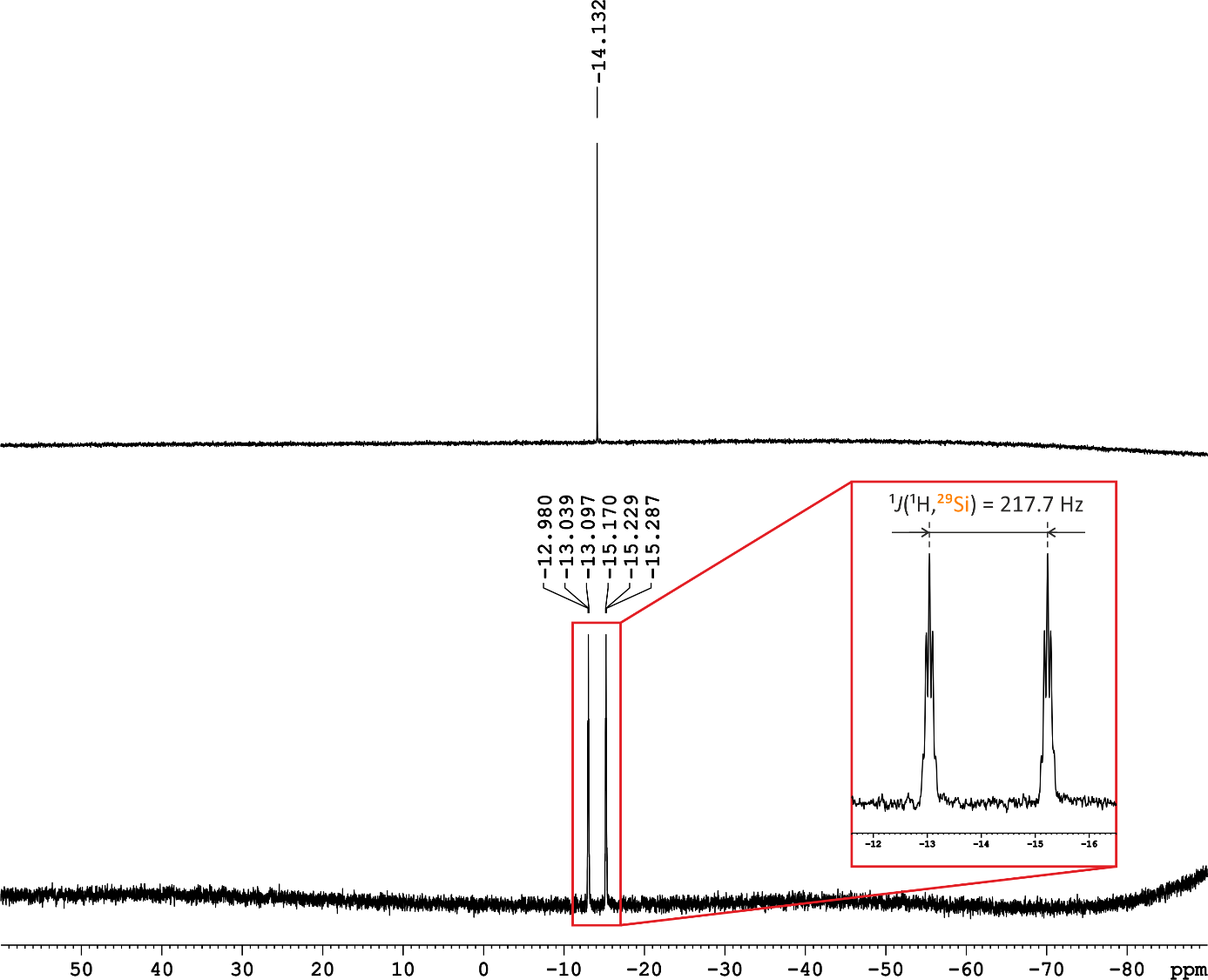

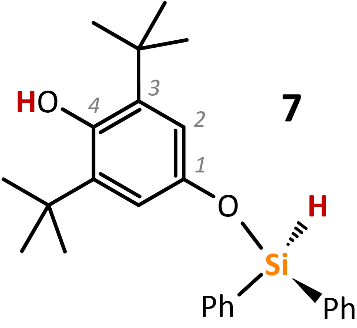


Figure S17: ^29^Si{^1^H} (top) and ^29^Si (bottom) NMR spectrum of isolated 7 in C_6_D_6_ (99.37 MHz, 295 K).

Catalytic synthesis of compound **6** from compound **7** using **I** as catalyst


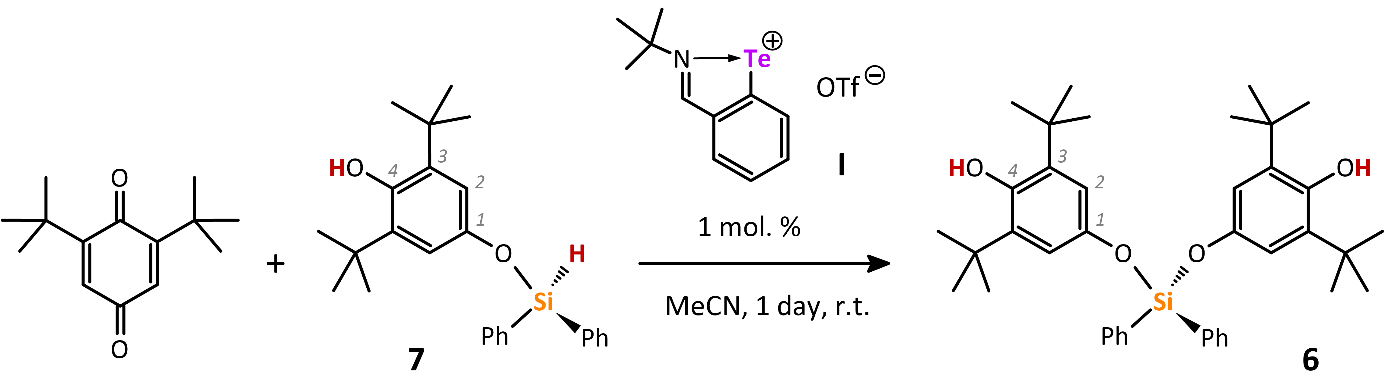


Scheme S7

3.6 mg (0.0084 mmol, 1 mol. %) of **I** (catalyst), 0.18 g (0.84 mmol) of 2,6-di-*tert*-butyl-1,4-benzoquinone (***p*-q(*t*Bu)_2_**) and 0.34 g (0.84 mmol) of compound **7** was loaded into a Schlenk tube and dissolved in 25 mL of dry and degassed acetonitrile under argon atmosphere (**Scheme S7**). The color of the solution slowly turned to orange by the generation of low steady-state concentration of blood red **II**. After a day of stirring, the resulting suspension of light orange solution and white powder (**6**) was evaporated and dried *in vacuo*. Based on NMR analysis of this crude evaporated reaction mixture in C_6_D_6_, conversion of **7** into **6** was >99 %. From this crude material, **6** was extracted from the catalyst by toluene extraction. By keeping the obtained solution overnight at 6 °C, white polycrystals of **6** (isolated yield 0.47 g, 89 %) were obtained. For NMR data of isolated **6** in C_6_D_6_ see the corresponding part above.

Catalytic synthesis of compound **8** using **I** as catalyst


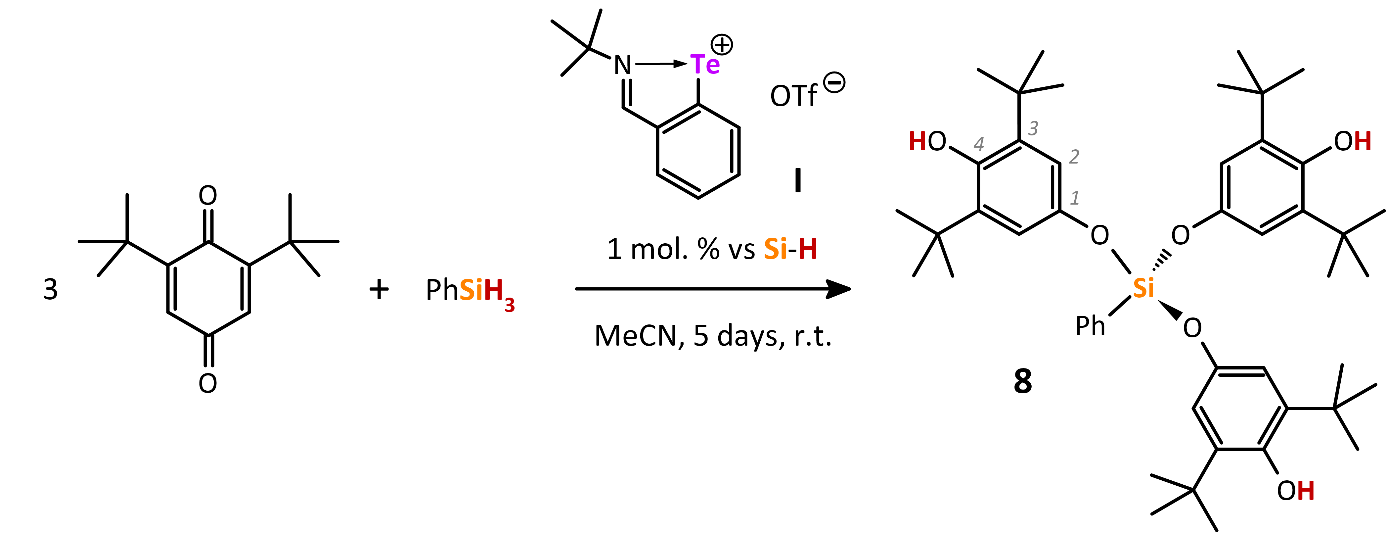


Scheme S8

49.9 mg (0.114 mmol, 1 mol. % *vs* Si-H) of **I** (catalyst) and 2.52 g (11.4 mmol) of 2,6-di-*tert*-butyl-1,4-benzoquinone (***p*-q(*t*Bu)_2_**) was loaded into a Schlenk tube and dissolved in 100 mL of dry and degassed acetonitrile under argon atmosphere. Subsequently, 0.471 μL (3.81 mmol) of neat phenylsilane PhSiH_3_ was added to this yellow solution under vigorous stirring (**Scheme S8**). After addition of the silane, the color of the solution slowly turned to orange due to the generation of low steady-state concentration of blood red **II**. After 5 days of stirring, the orange color of the solution finally faded into light orange. The reaction mixture was evaporated at low pressure and properly dried *in vacuo* to form a foamy material. From this foamy material, product **8** was extracted by boiling hexane (30 mL). The resulting yellowish filtrate was evaporated at low pressure into yellowish powder of **8**. The powder of **8** was recrystallized from a minimal amount of boiling hexane, by slow cooling of the obtained solution to r.t., to give yellowish single-crystals of **8** (isolated yield 2.73 g, 93 %; m.p. = 143 – 145 °C). Conversion of the reaction is >99 % based on NMR analysis of the foamy material in C_6_D_6_ with no ***p*-q(*t*Bu)_2_** left.

*NMR data for isolated* ***8*** *in C_6_D_6_:*

**^1^H NMR** (500.20 MHz, C_6_D_6_) δ (ppm): 1.27 [54H, s, 6x (C*H*_3_)_3_C-]; 4.56 [3H, s, 3x O*H*]; 7.12–7.16 [3H, m, *p*-PhSi and *m*-PhSi]; 7.17 [6H, s, 6x Ar(C2)*H*]; 8.11 [2H, m, *o*-PhSi]. **^13^C{^1^H} NMR** (125.78 MHz, C_6_D_6_) δ (ppm): 30.6 [s, 6x (*C*H_3_)_3_C-]; 34.8 [s, qC, 6x (CH_3_)_3_*C*-]; 117.0 [s, 6x Ar-(*C2*)H]; 128.6 [s, *m*-*C*H, PhSi]; 130.9 [qC, *ipso*-*C*, PhSi]; 131.6 [s, *p*-*C*H, PhSi]; 135.8 [s, *o*-*C*H, PhSi]; 137.6 [s, qC, 6x Ar-(*C3*)]; 147.4 [s, qC, 3x Ar-(*C1 or 4*)]; 149.5 [s, qC, 3x Ar-(*C1 or 4*)]. **^29^Si{^1^H} NMR** (99.38 MHz, C_6_D_6_) δ: −69.2 ppm


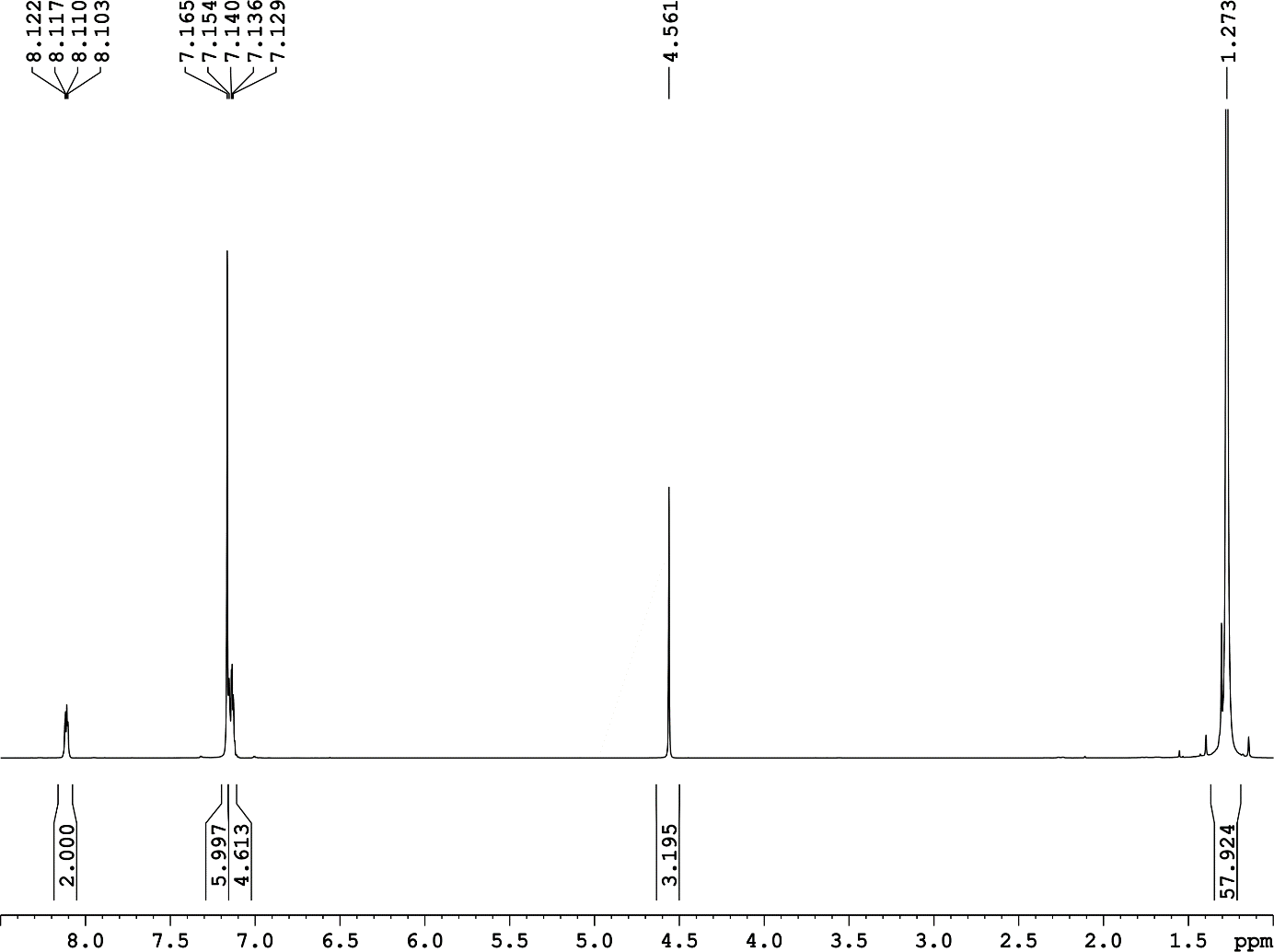

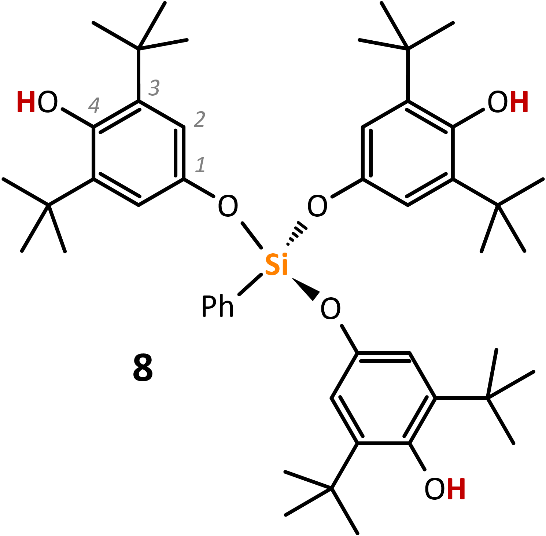


Figure S18: ^1^H NMR spectrum of isolated 8 in C_6_D_6_ (500.20 MHz, 295 K).


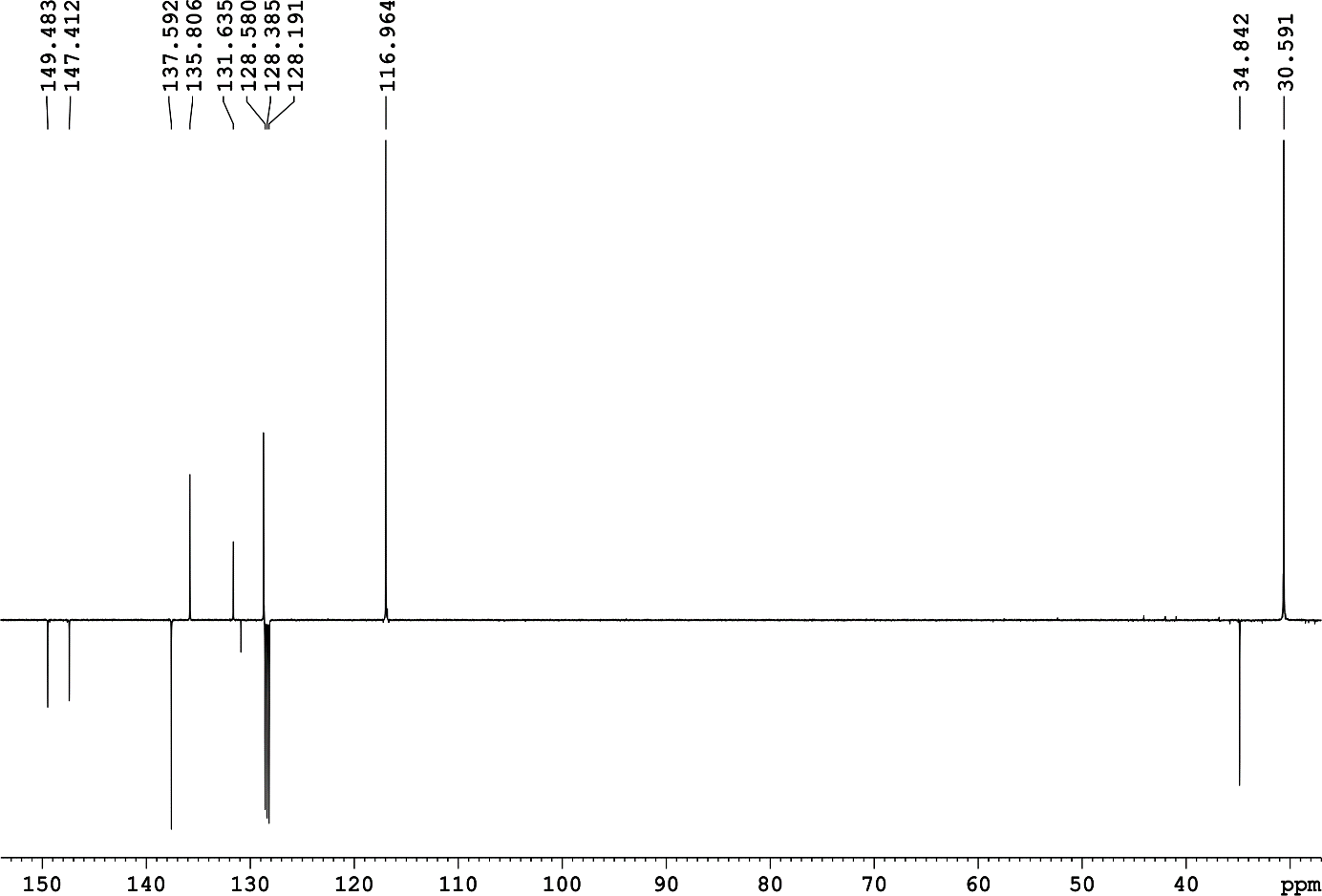

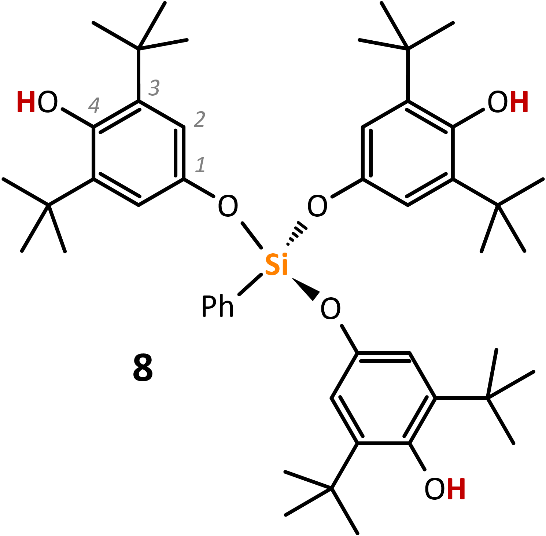


Figure S19: ^13^C{^1^H} APT NMR spectrum of isolated 8 in C_6_D_6_ (125.78 MHz, 295 K).


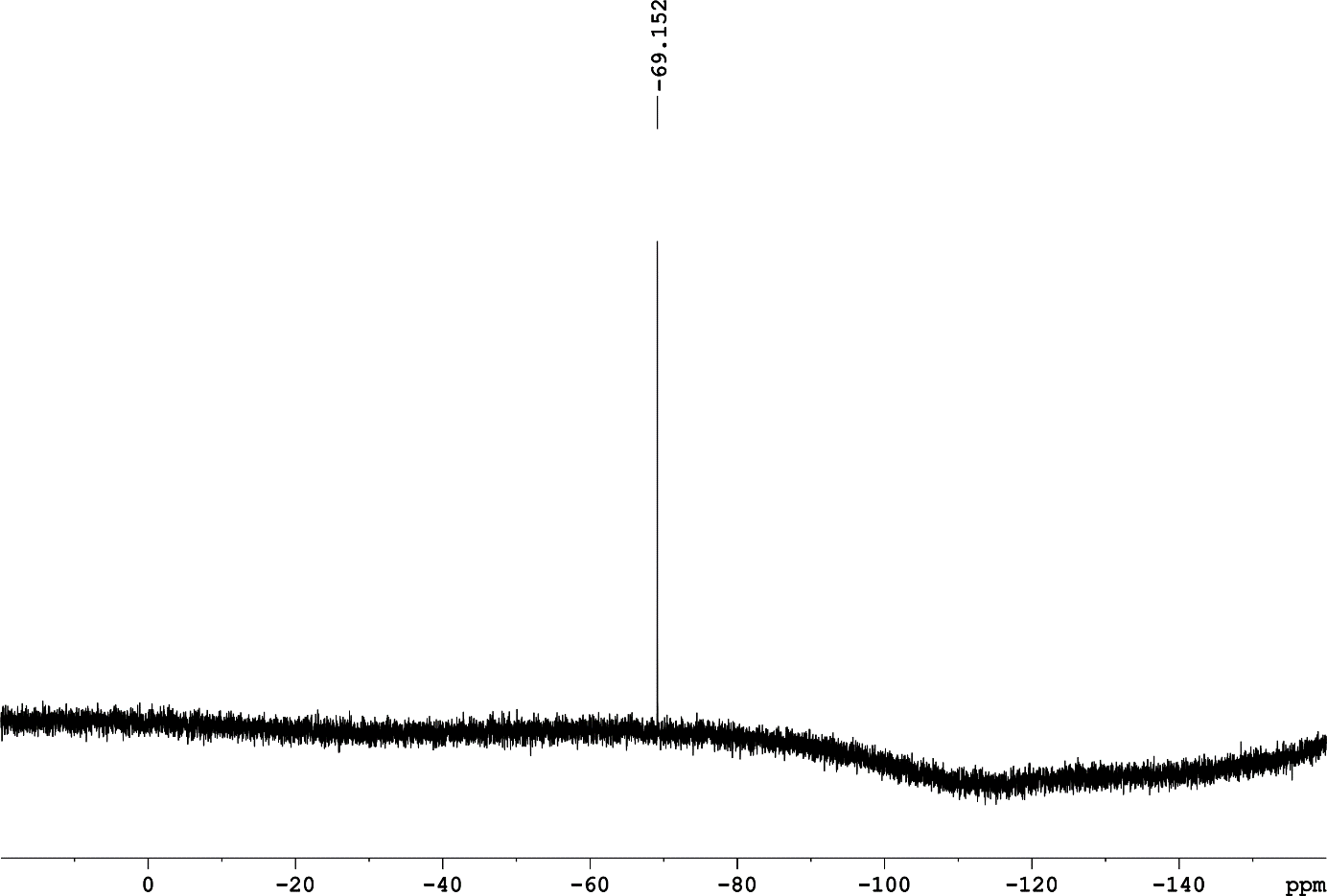

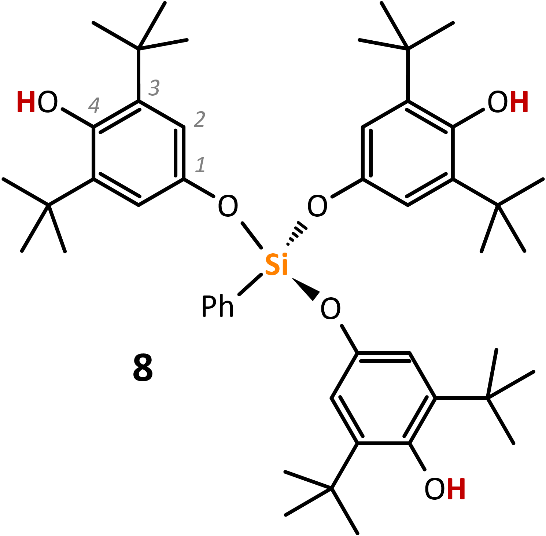


Figure S20: ^29^Si{^1^H} NMR spectrum of isolated 8 in C_6_D_6_ (99.37 MHz, 295 K).

Catalytic synthesis of compound **9** using **I** as catalyst


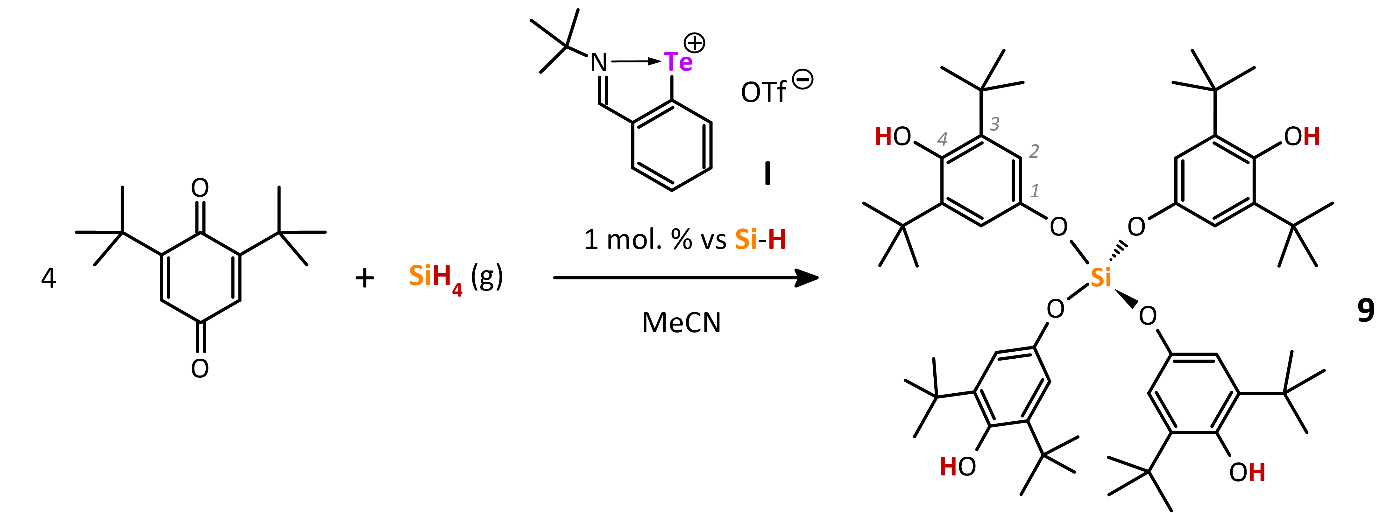


Scheme S9

**Caution:** SiH_4_ is a pyrophoric gas. For safety reasons, only small amount of SiH_4_ was *in situ* prepared inside evacuated and sealed vessel with enough ***p*-q(*t*Bu)_2_** to assure that all the generated SiH_4_ will be consumed.


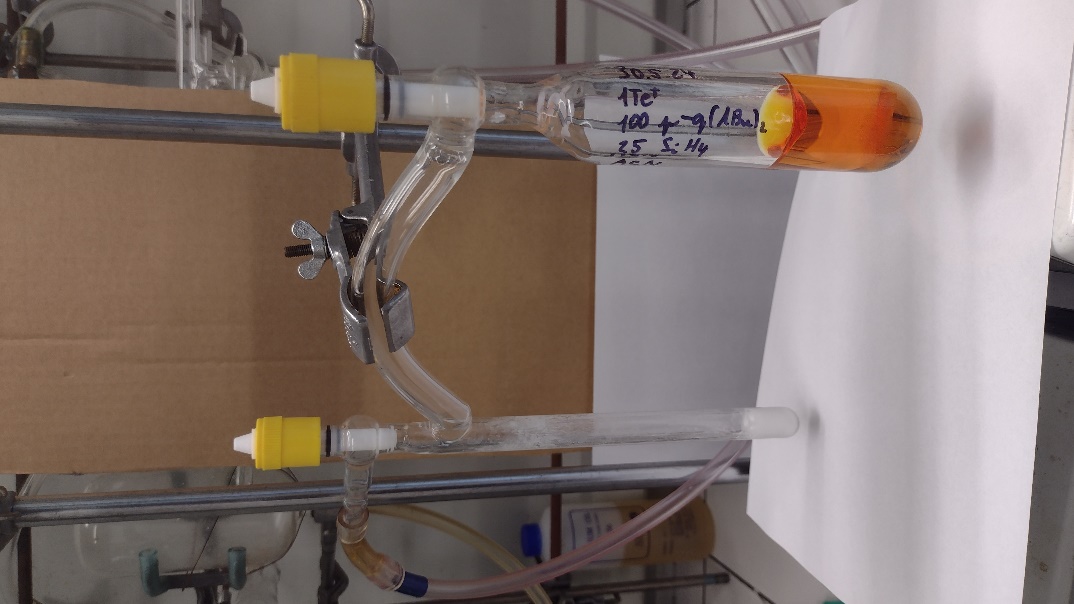

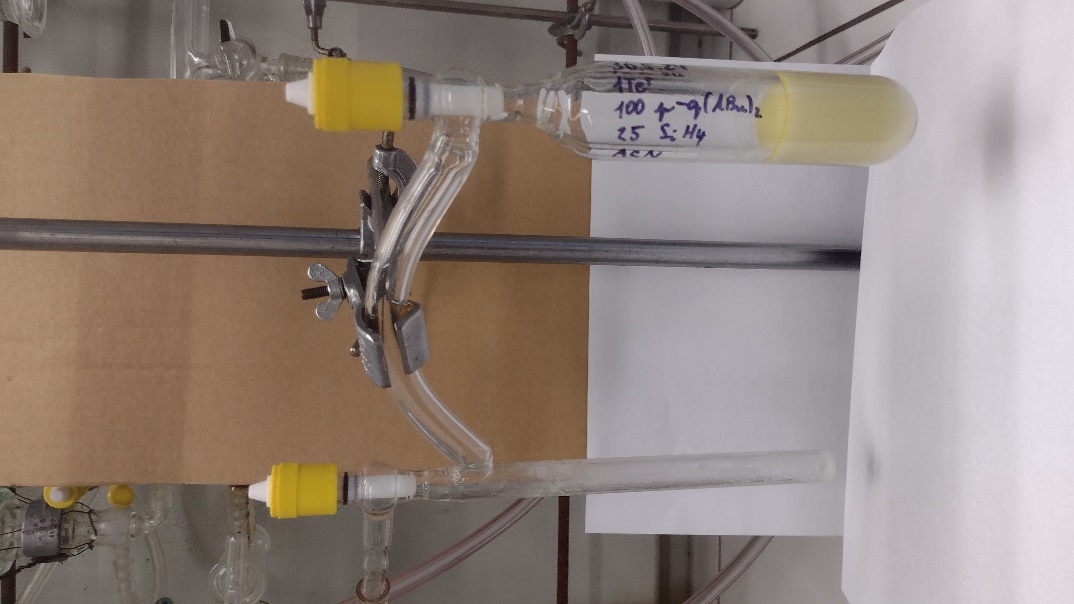


Figure S21: Photos of used apparatus for synthesis of compound 9. The left picture was taken 30 minutes after initialization of the reaction (described in the text below). The picture on right was taken after 3 days and it shows product 9 precipitated in the right vessel (the vessel closer to the camera)

For the synthesis of compound **9** (**Scheme S9**), a specially made glassware equipped with two PTFE Young valves was used (see **Figure S21** above; the exact same piece of glassware can be used also for vacuum transfer of deuterated solvents or other volatile liquids). One Young valve (on left on pictures) allows to disconnect the apparatus from the Schlenk line (inert/vacuum line) while the second (on right on pictures) allows to close the connection between both reaction vessels.

The left vessel of this specially made glassware was equipped with a stirring bar and loaded under argon atmosphere with 7.2 mL of 0.26 M Et_2_O solution of LiAlH_4_ (i.e. 1.868 mmol, 150 % of theoretical amount; *the solution was made by refluxing solid LiAlH_4_ in Et_2_O and the concentration was determined by titration using a standardized solution of H_2_SO_4_ and phenolphthalein as an indicator*).

The right vessel of the apparatus was equipped with a stirring bar and loaded with a solution of 22.0 mg (0.0504 mmol, 1 mol. % *vs* Si-H) of **I** (catalyst) and 1.10 g (5.04 mmol) of 2,6-di-*tert*-butyl-1,4-benzoquinone (***p*-q(*t*Bu)_2_**) dissolved in 25 mL of dry and degassed acetonitrile.

Afterwards, the left side containing the solution of LiAlH_4_ was cooled down to −50 °C and THEN, using a septum, neat SiCl_4_ (0.143 mL, 1.26 mmol) was added via a syringe to this solution. Keeping the temperature so low, LiAlH_4_ and SiCl_4_ do not react despite they are mixed together.^[2^[^5^](#_ENREF_5)^]^ Then, the septum on the left was exchanged for a PTFE Young plug and both solutions of both sides of this apparatus were frozen in liquid nitrogen and then finally fully evacuated by active vacuum. After about 1 minute of evacuation, the Young valve on the left was closed (so the apparatus was fully disconnected from the Schlenk line), however, the Young valve on right was kept open. Then, the liquid nitrogen bath from both reaction vessels were removed so the contents of each reaction vessels could melt. When the temperature of left side (containing the mixture of LiAlH_4_ and SiCl_4_) reached approx. 0 °C, these reagents started to slowly react, i.e. started releasing gaseous SiH_4_. The generated SiH_4_ was slowly absorbed by the solution of the right vessel containing the catalyst **I** and ***p*-q(*t*Bu)_2_** which outwardly manifested by changing its light-yellow color to orange caused by the generation of low steady-state concentration of blood red **II**. By the 3^rd^ day of stirring at r.t., the orange solution turned into a white suspension. The suspension was filtered and the obtained white precipitate of **9** was washed by 5 mL of acetonitrile and dried in vacuo to give 980 mg (86 %) of **9** as white powder. Compound **9** was recrystallized from a minimal amount of boiling toluene, and by slow cooling of the obtained solution to r.t., colorless single-crystals of **9** (m.p. = 213 – 215 °C) were obtained. Conversion of the reaction is >99 % based on NMR analysis of evaporated reaction mixture in C_6_D_6_ with no ***p*-q(*t*Bu)_2_** left.

*NMR data for isolated* ***9*** *in C_6_D_6_:*

**^1^H NMR** (500.20 MHz, C_6_D_6_) δ (ppm): 1.29 [72H, s, 8x (C*H*_3_)_3_C-]; 4.59 [4H, s, 4x O*H*]; 7.25 [8H, s, 8x Ar(C2)*H*]. **^13^C{^1^H} NMR** (125.78 MHz, C_6_D_6_) δ (ppm): 30.6 [s, (*C*H_3_)_3_C-]; 34.8 [s, qC, (CH_3_)_3_*C*-]; 116.8 [s, Ar-(*C2*)H]; 137.7 [s, qC, Ar-(*C3*)]; 146.9 [s, qC, Ar-(*C1 or 4*)]; 149.8 [s, qC, Ar-(*C1 or 4*)]. **^29^Si{^1^H} NMR** (99.38 MHz, C_6_D_6_) δ: −98.6 ppm


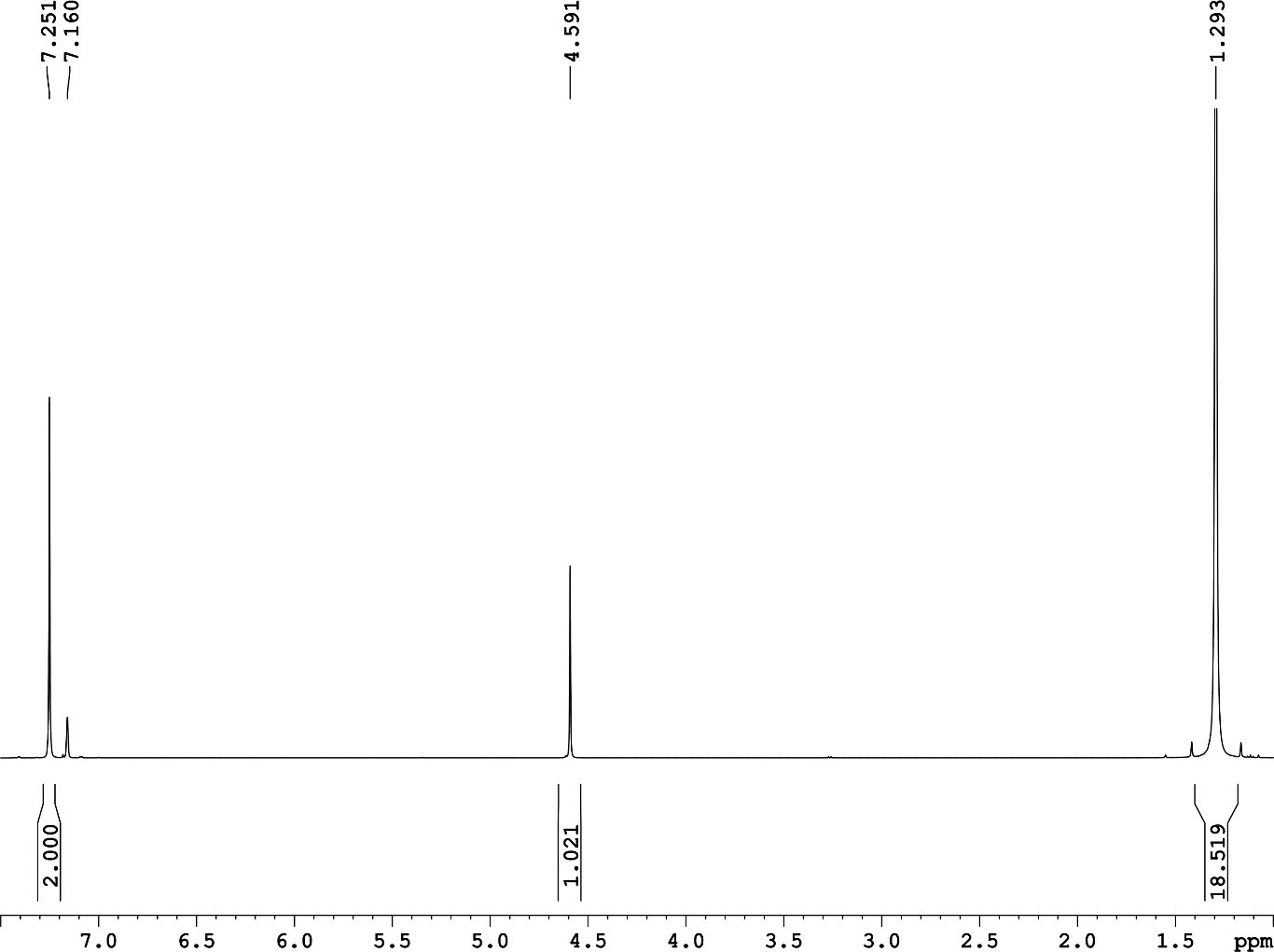

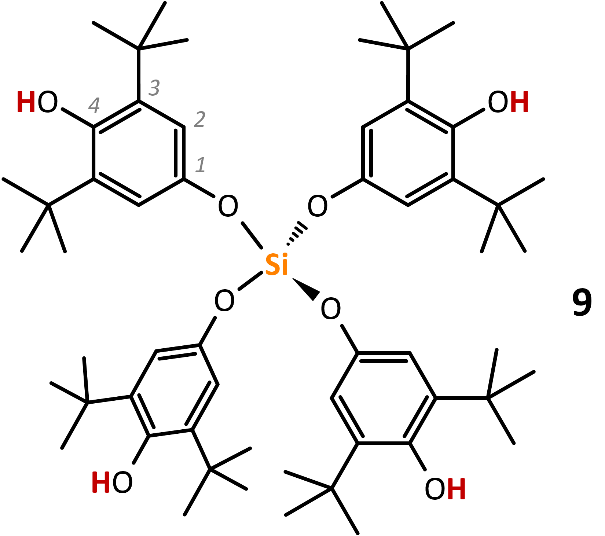


Figure S22: ^1^H NMR spectrum of isolated 9 in C_6_D_6_ (500.20 MHz, 295 K).


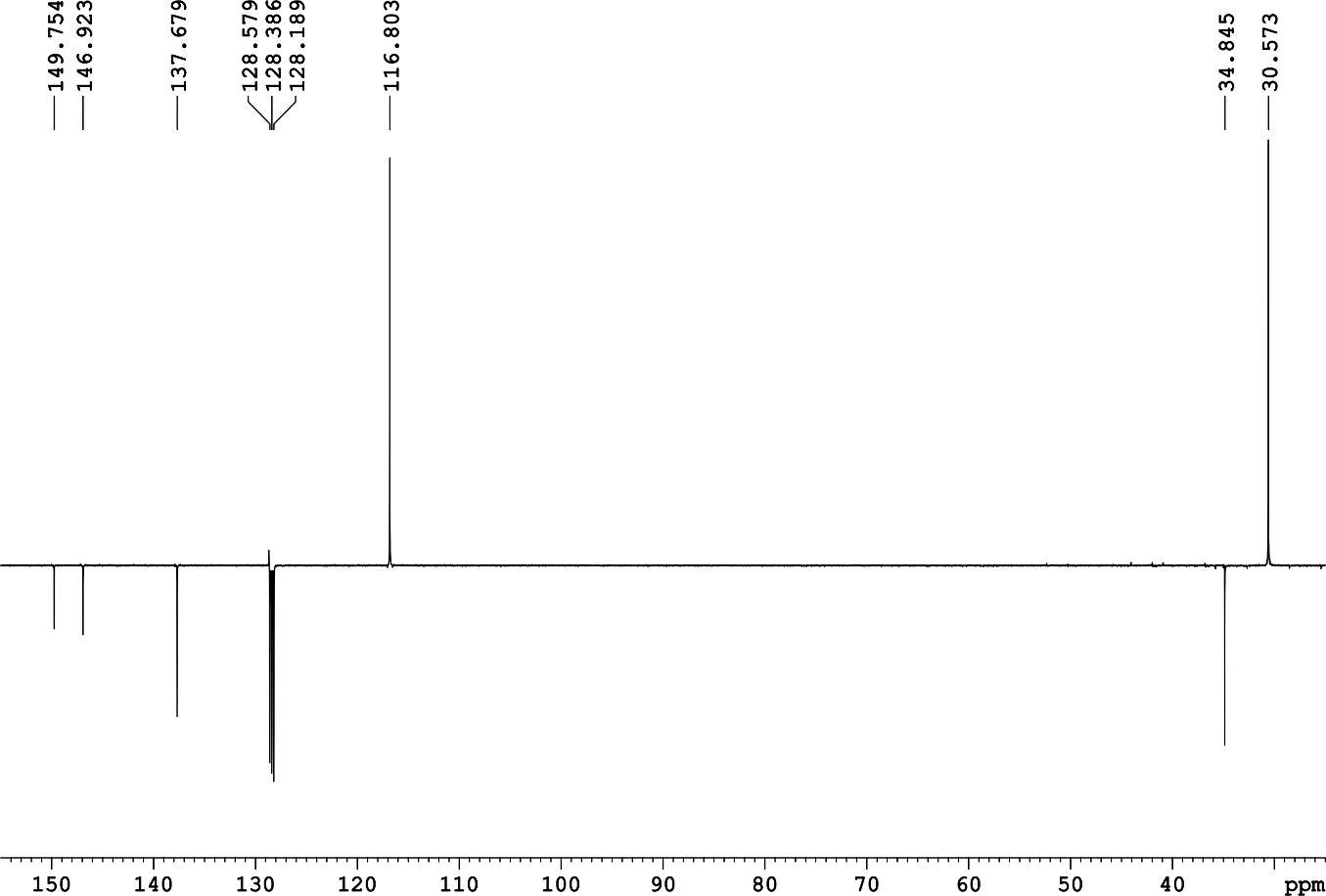

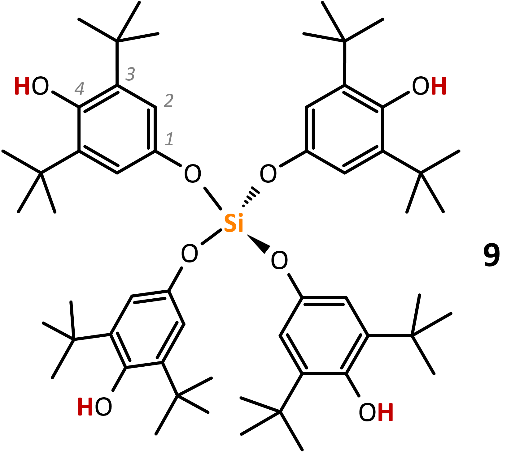


Figure S23: ^13^C{^1^H} APT NMR spectrum of isolated 9 in C_6_D_6_ (125.78 MHz, 295 K).


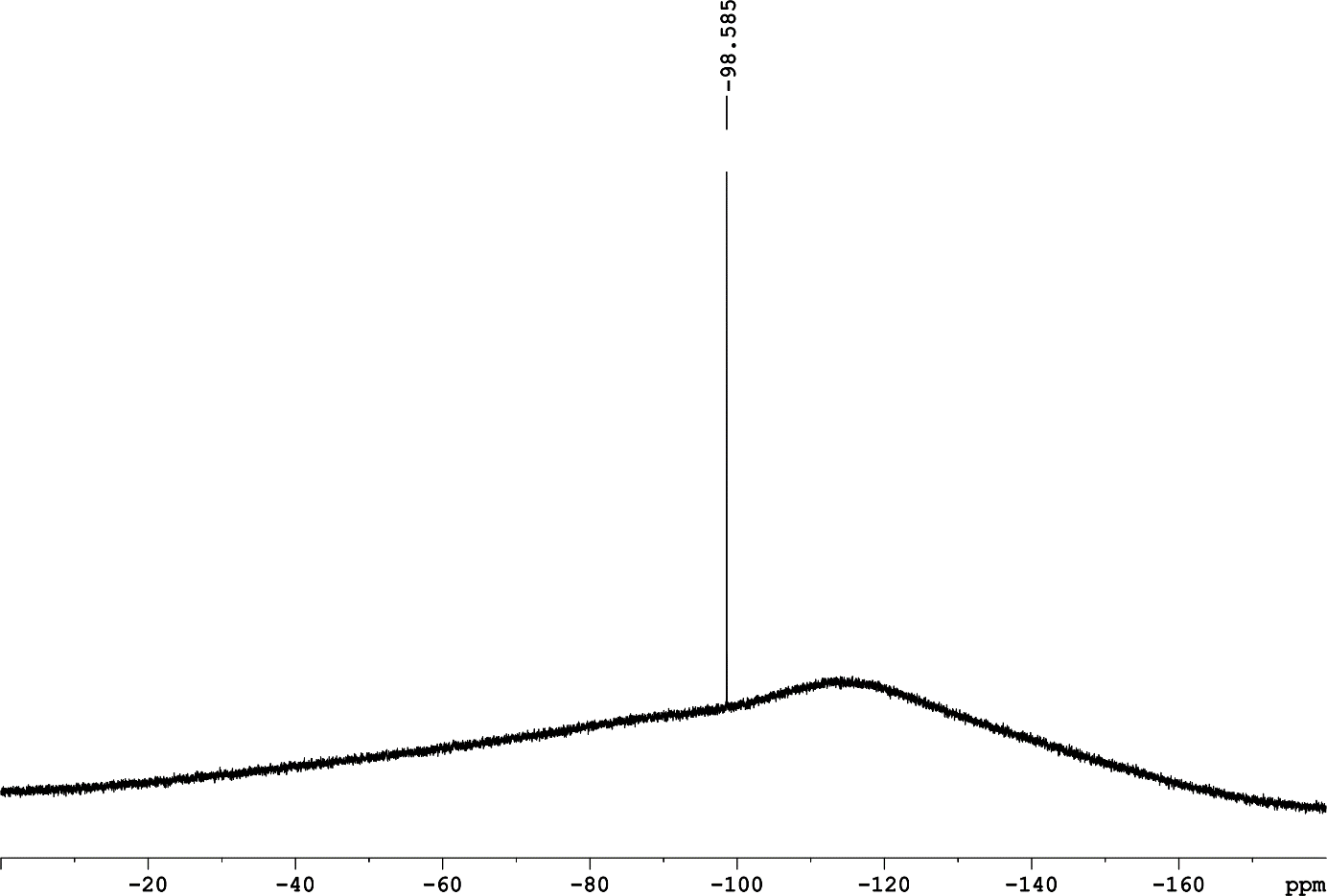

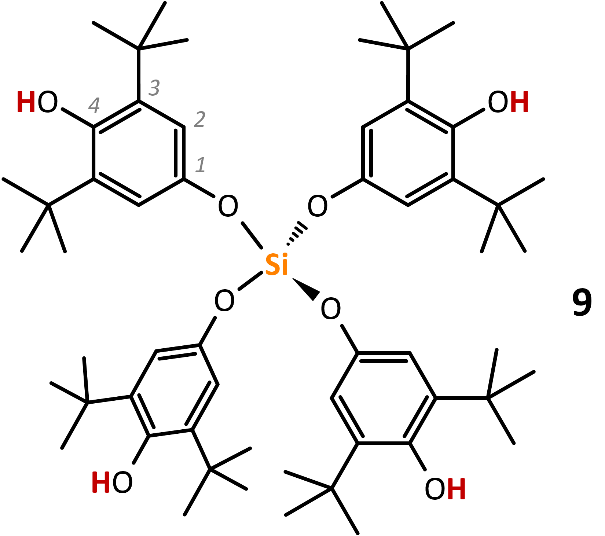


Figure S24: ^29^Si{^1^H} NMR spectrum of isolated 9 in C_6_D_6_ (99.37 MHz, 295 K).

***Mechanistic study***

Control experiments at stochiometric reaction conditions


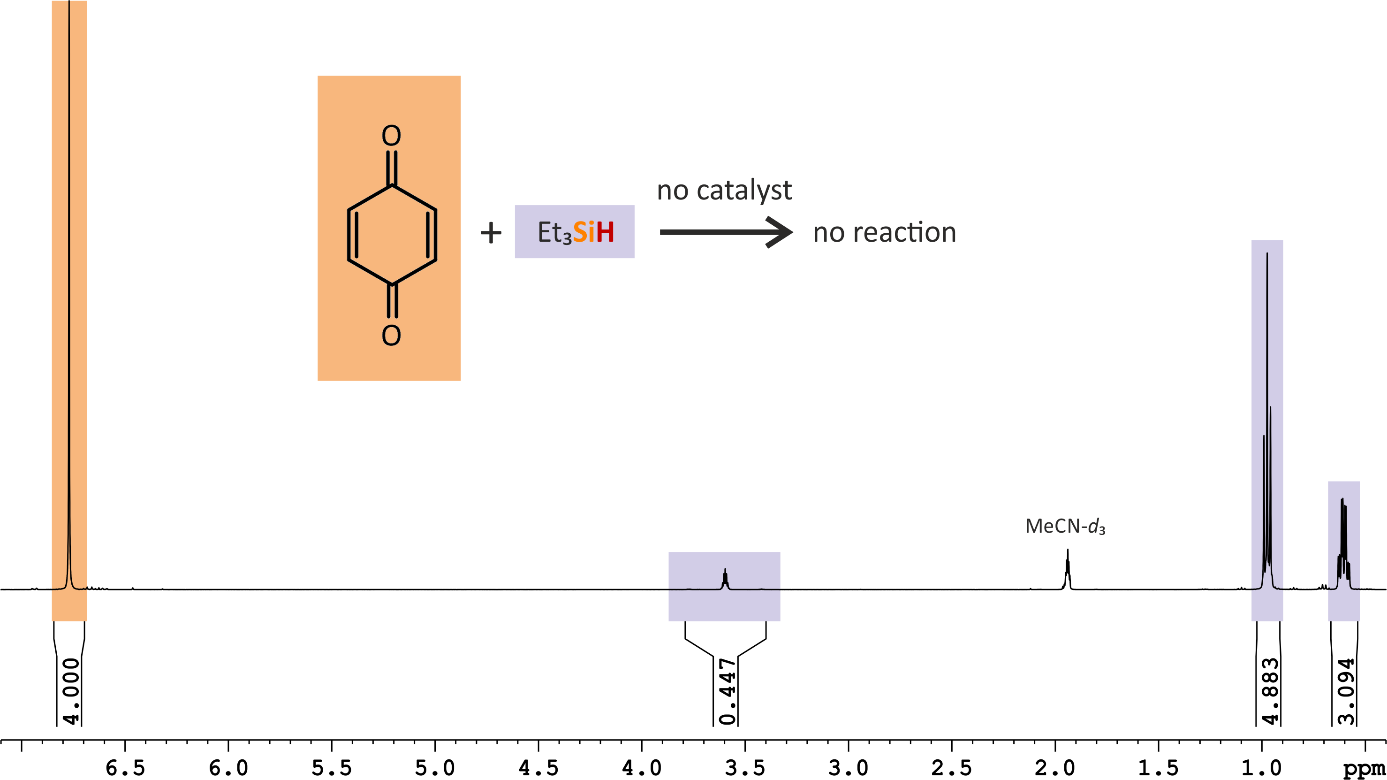


Figure S25: ^1^H NMR spectrum of mixture of *p*-q with Et_3_SiH showing no reaction without addition of the catalyst I (500.20 MHz, 295 K, MeCN-*d*_3_).


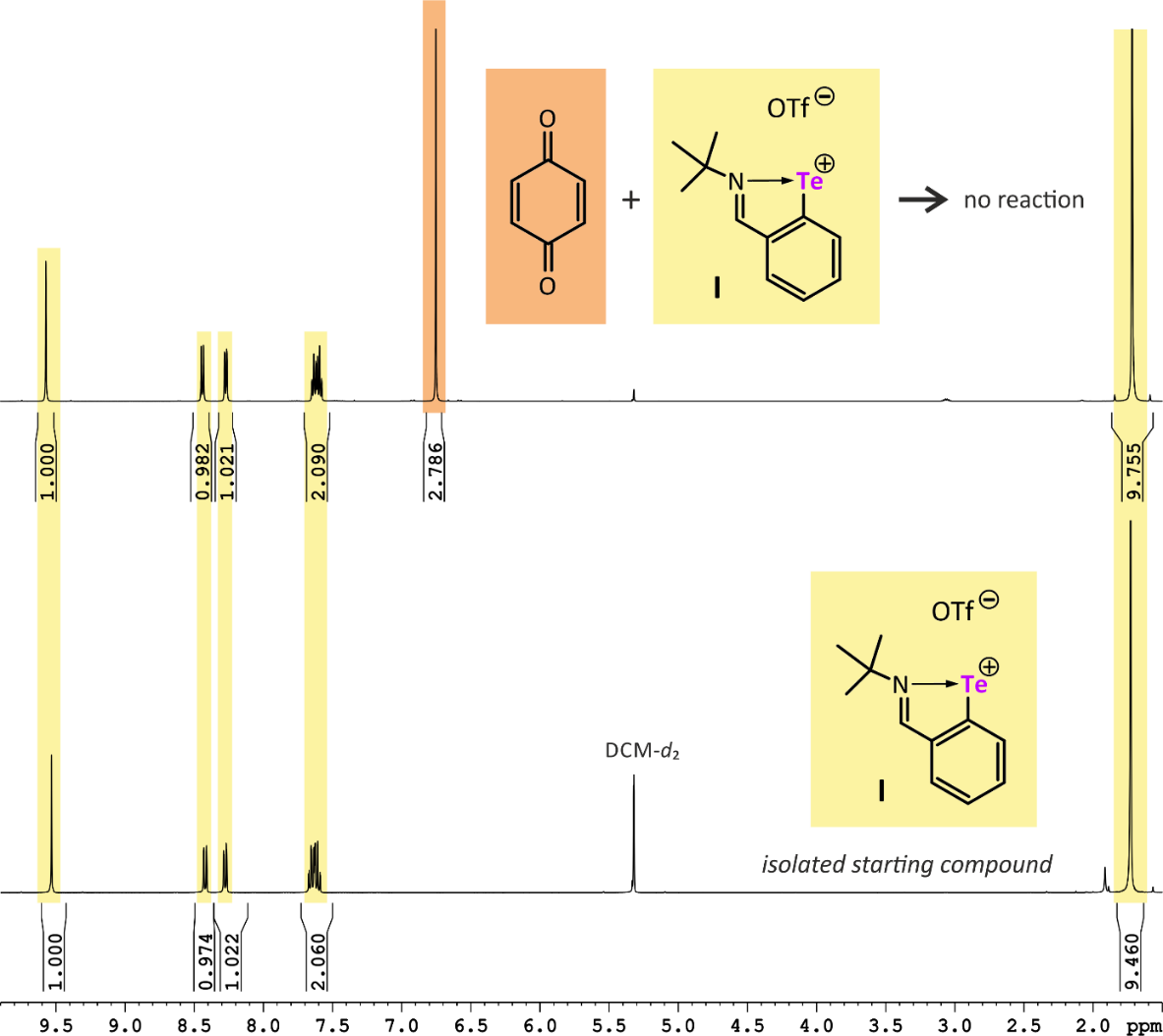


Figure S26: Stacked plot of ^1^H NMR spectra of mixture of *p*-q with I showing no reaction (top) and of isolated I for comparison (bottom). Both spectra at 500.20 MHz, 295 K, DCM-*d*_2_).

A role of proton scrambling in **II** for the catalysis

In our previous paper^[6]^ we have shown that the proton in the mono-iminium ditelluride **II** undergoes intramolecular scrambling leading to di-iminium ditelluride **IIb** and non-protonated imino ditelluride **IIa** (**Scheme S10**):


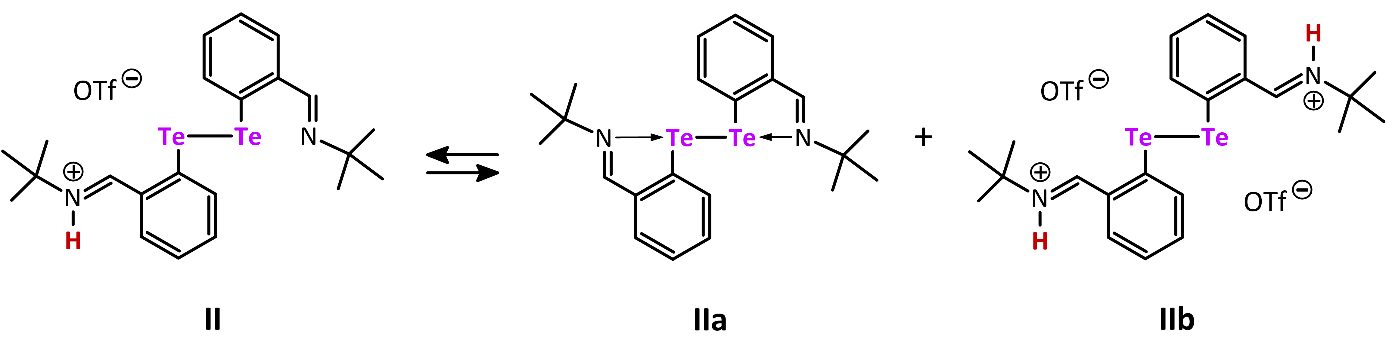


Scheme S10: Proton scrambling between ditellurides described in our previous paper.^[6]^

Such a process raised the question whether **IIa** or **IIb** play any important role during the catalysis. To answer this question, the isolated compounds **IIa** and **IIb** were each reacted with ***p*-q** (**Scheme S11**). Under stoichiometric conditions the non-protonated bis(imino) ditelluride **IIa** reacts with ***p*-q** in both DCM-*d*_2_ and MeCN-*d*_3_ quantitatively to a product, in which ***p*-q** inserts into the Te-Te bond, i.e. leading to compound **IV** (see **Scheme S11** below). Importantly, the reaction takes about a month to completion regardless of DCM-*d*_2_ or MeCN-*d*_3_ is used as a solvent, hampering the potential of **IIa** and **IIb** to be effective species during the catalytic cycle. Further discussion follows respective synthetic part.


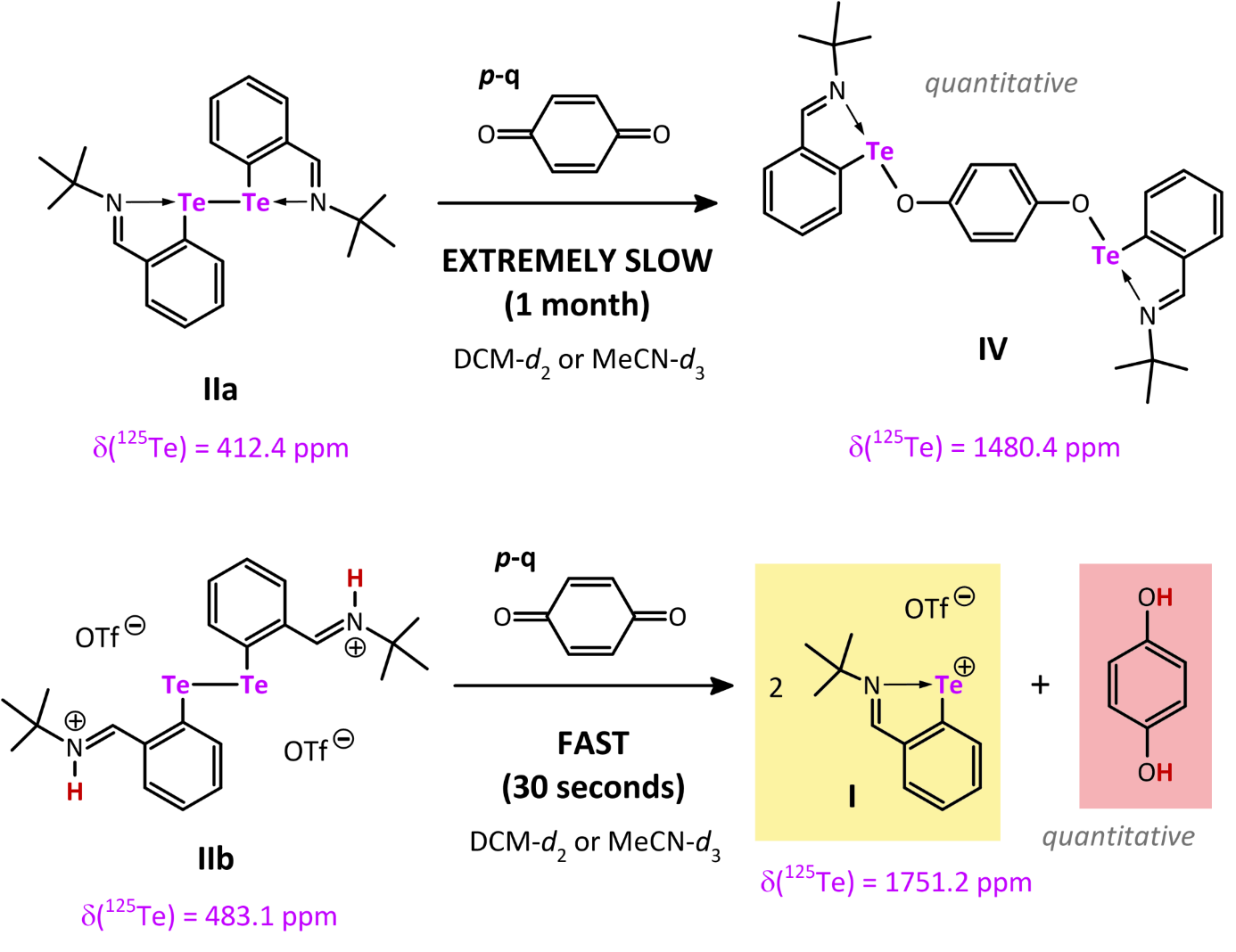


Scheme S11: Reactivity of isolated ditellurides IIa and IIb with *p*-q.


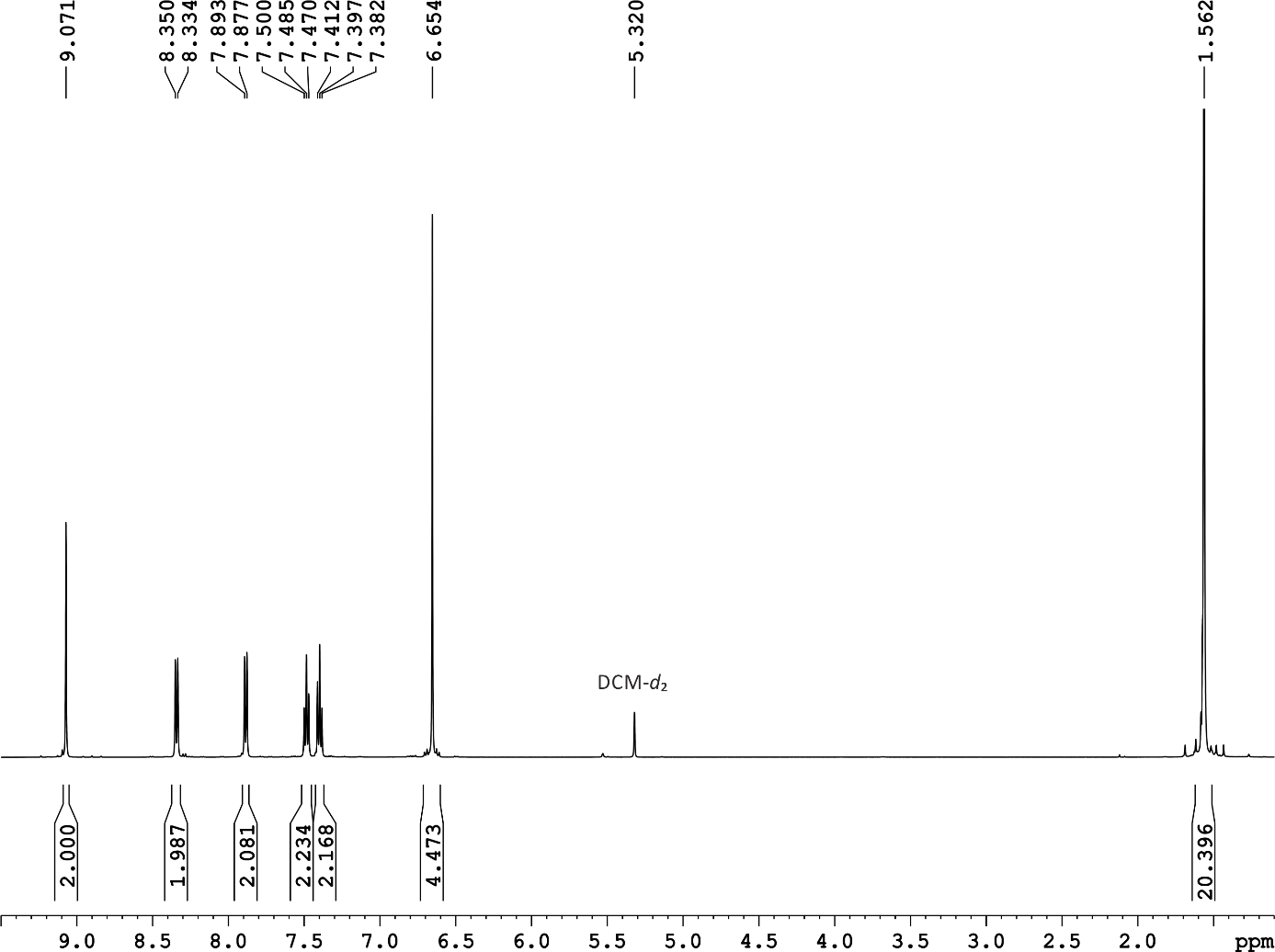

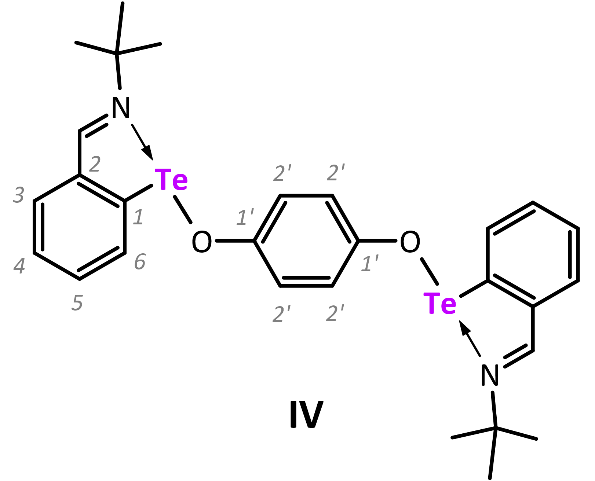


Figure S27: ^1^H NMR spectrum of isolated IV in DCM-*d*_2_ (500.20 MHz, 295 K).


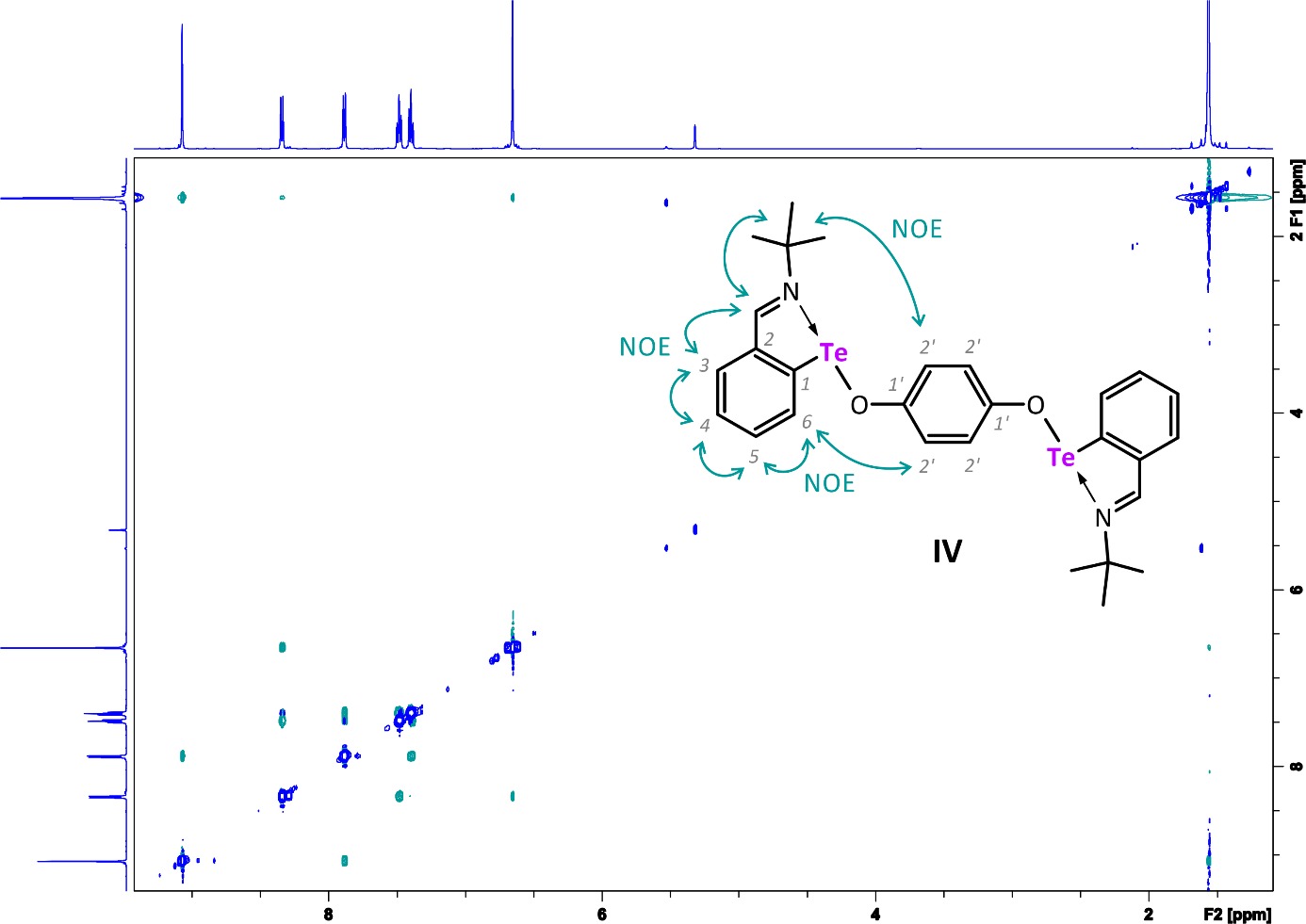


Figure S28: ^1^H-^1^H NOESY/EXSY NMR spectrum of isolated IV in DCM-*d*_2_ (500.20 MHz, 295 K, d8 = 2 s).


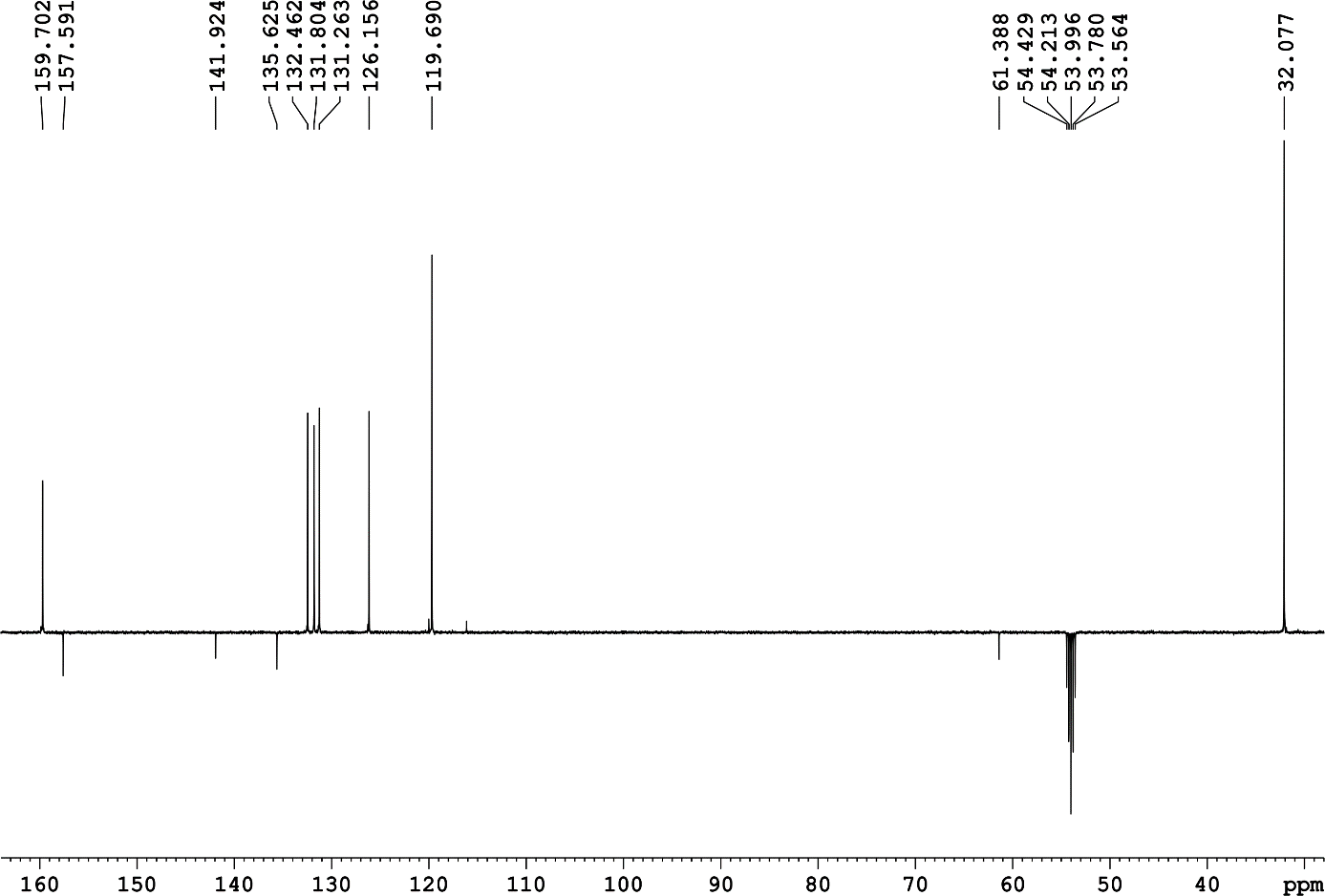

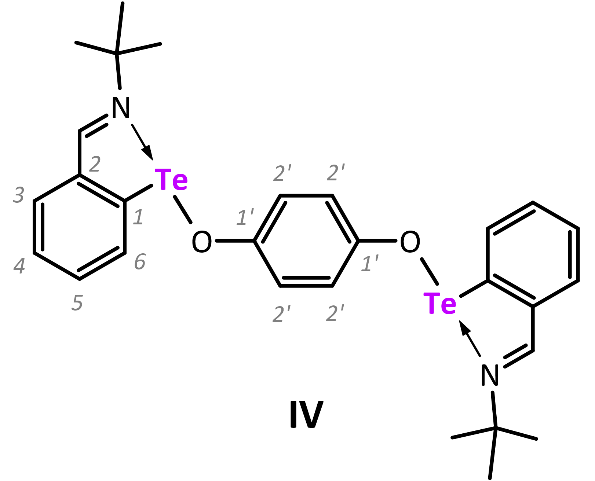


Figure S29: ^13^C{^1^H} APT NMR spectrum of isolated IV in DCM-*d*_2_ (125.78 MHz, 295 K).


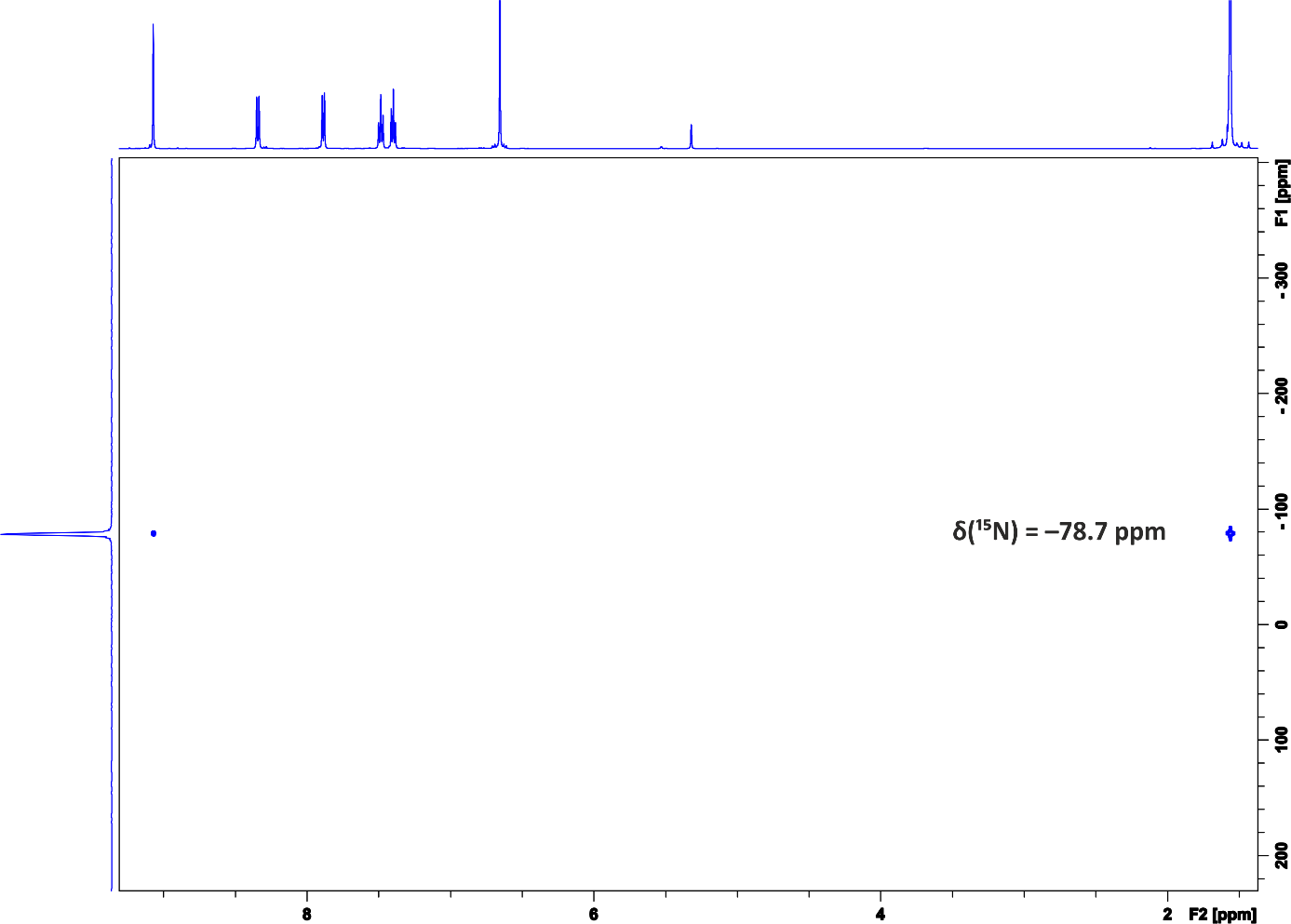

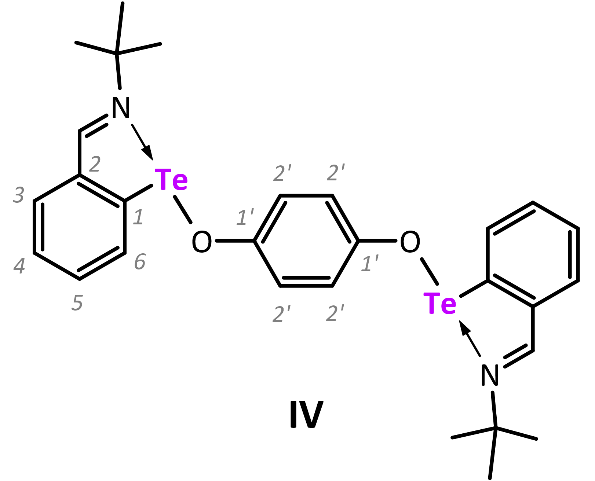


Figure S30: ^1^H-^15^N HMBC NMR spectrum of isolated IV in DCM-*d*_2_ (500.20 MHz, 295 K).


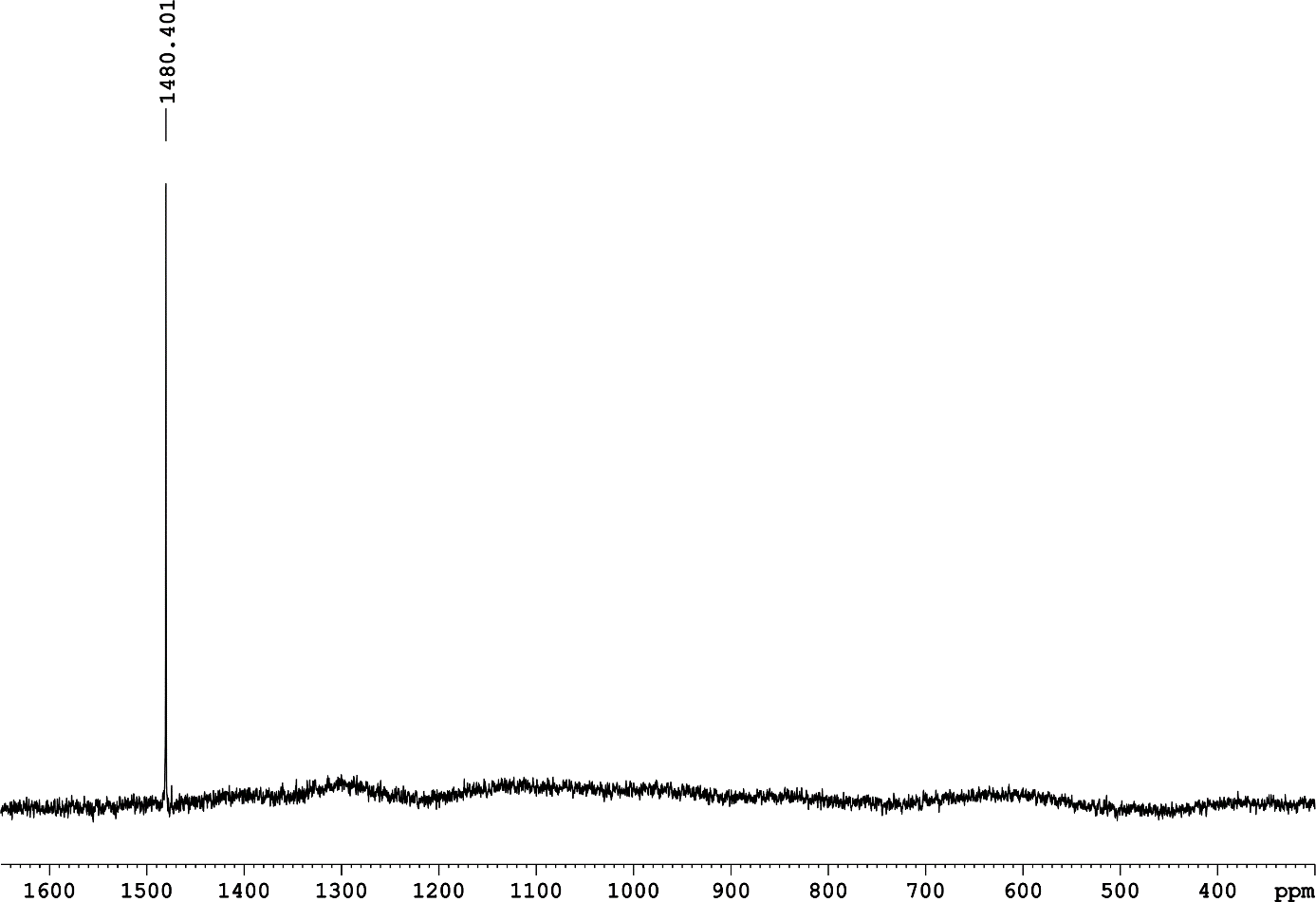

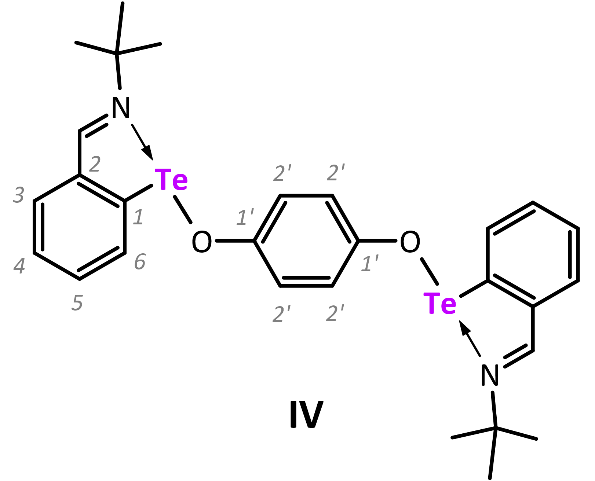


Figure S31: ^125^Te{^1^H} NMR spectrum of isolated IV in DCM-*d*_2_ (157.79 MHz, 295 K, NS = 51200).

Synthesis of ***IV***:

139 mg (0.241 mmol) of di-imino ditelluride **IIa** and 26 mg (0.241 mmol) 1,4-benzoquinone (***p*-q**) was loaded into a Young valve container and dissolved in 3 mL of dry and degassed DCM-*d*_2_. After 10 minutes of stirring, a sample of the obtained yellow solution was transferred into NMR tube, flame-sealed and analyzed. It turned out that it takes about a month to completion the reaction (>99 %) into **IV**, which outwardly manifests by gradual darkening of the reaction mixture into black-brown solution. After a month of stirring of the content in the sealed Young container, the black-brown solution was concentrated at low pressure to 1 mL and a hexane was added (2 mL) to crystalize the compound **IV**. By staying the solution at room temperature, 101 mg (61 %) of dark brown polycrystals of **IV** was obtained (m.p. 199 – 201 °C). Unfortunately, no single crystals suitable for sc-X-ray diffraction analysis were obtained.


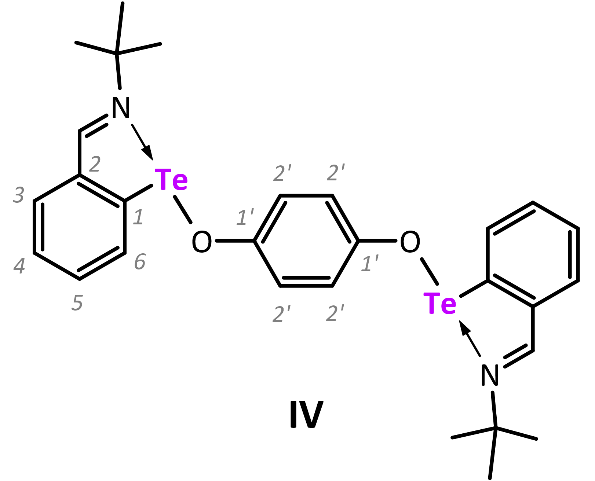


*NMR data for isolated* ***IV*** *in DCM-d_2_:*

**^1^H NMR** (500.20 MHz, DCM-*d*_2_) δ (ppm): 1.56 [18H, s, 2x (C*H*_3_)_3_C-]; 6.65 [4H, m, 4x (C2’)*H*, i.e. O-C_6_*H_4_*-O]; 7.40 [2H, t, 2x Ar(C4)*H*]; 7.49 [2H, t, 2x Ar(C5)*H*]; 7.89 [2H, d, 2x Ar(C3)*H*]; 8.34 [2H, d, 2x Ar(C6)*H*]; 9.07 [2H, s, 2x C*H*=N]. **^13^C{^1^H} NMR** (125.78 MHz, DCM-*d*_2_) δ (ppm): 32.1 [s, (*C*H_3_)_3_C-]; 61.4 [s, qC, (CH_3_)_3_*C*-]; 119.7 [s, (*C2’)*H) of O-C_6_H_4_-O]; 126.2 [s, Ar(*C4*)H]; 131.3 [s, Ar(*C6*)H]; 131.8 [s, Ar(*C5*)H]; 132.5 [s, Ar(*C3*)H]; 135.6 [s, qC, Ar(*C1 or C2*)]; 141.9 [s, qC, Ar(*C1 or C2*)]; 157.6 [s, qC, 2x (*ipso*)*C1’-*OTe_2_]; 159.7 [s, CH=*N*]. **^15^N NMR** (40.54 MHz, DCM-*d*_2_) δ: –78.7 ppm [CH=*N*🡪Te]. **^125^Te NMR** (157.79 MHz, DCM-*d*_2_) δ: 1480.4 ppm [s].

Noteworthy, the extremely long reaction time for full conversion into **IV** means that the formation of such species cannot play any crucial role in our presented catalytic cycle as normally these catalytic reactions take from hours to days to be finished at catalytic conditions. Yet, once compound **IV** is formed, it cleanly reacts with silyl triflates such as Me_3_SiOTf in a 1:2 molar ratio under formation of 2 eq. of the tellurenyl species **I** and the bis(silylated) hydroquinone **2^Me^** (**Scheme S12**). Besides, the same species **IV** also cleanly reacts with HOTf in a 1:2 molar ratio under formation of 2 eq. of the tellurenyl species **I** and hydroquinone (**Scheme S12**). Most importantly, the exact same products are formed in analogous reactions when **IIIa** is used instead of **IV** (see **Scheme S16** and figures below). Thus, the potential involvement of **IIa** in the catalysis, slowly leading to compound **IV**, has no impact on the products produced during the catalysis.


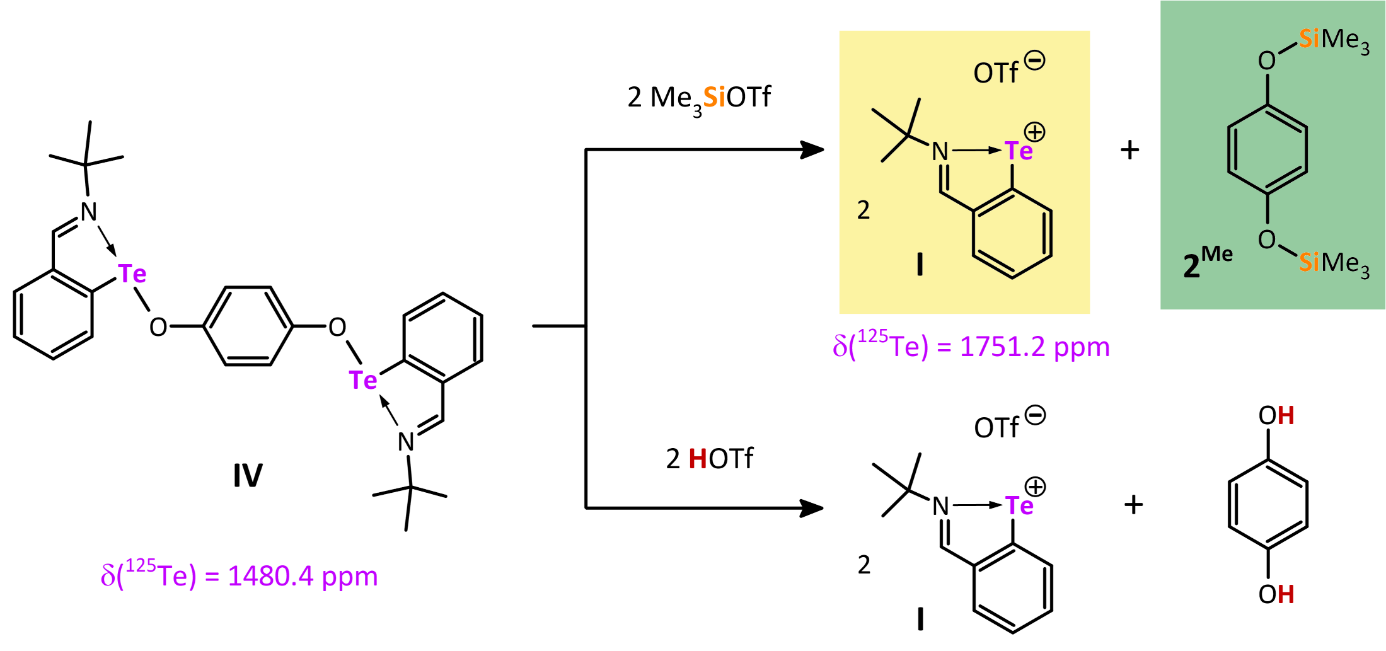


Scheme S12: Reactivity of isolated IV with 2 eq. of Me_3_SiOTf or HOTf.


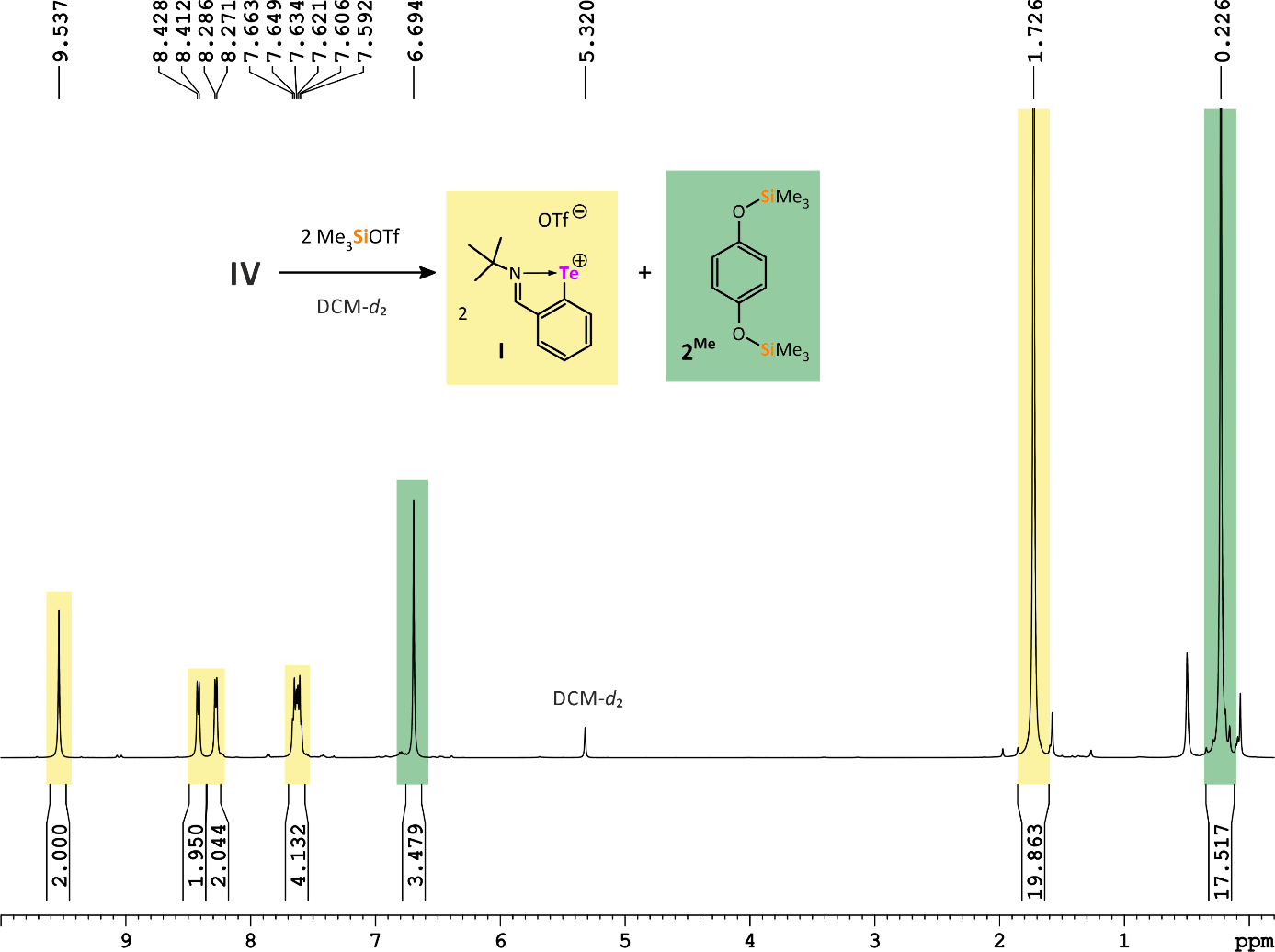


Figure S32: ^1^H NMR spectrum of rection mixture from reaction of IV with 2 eq. of Me_3_SiOTf in DCM-*d*_2_ (500.20 MHz, 295 K).


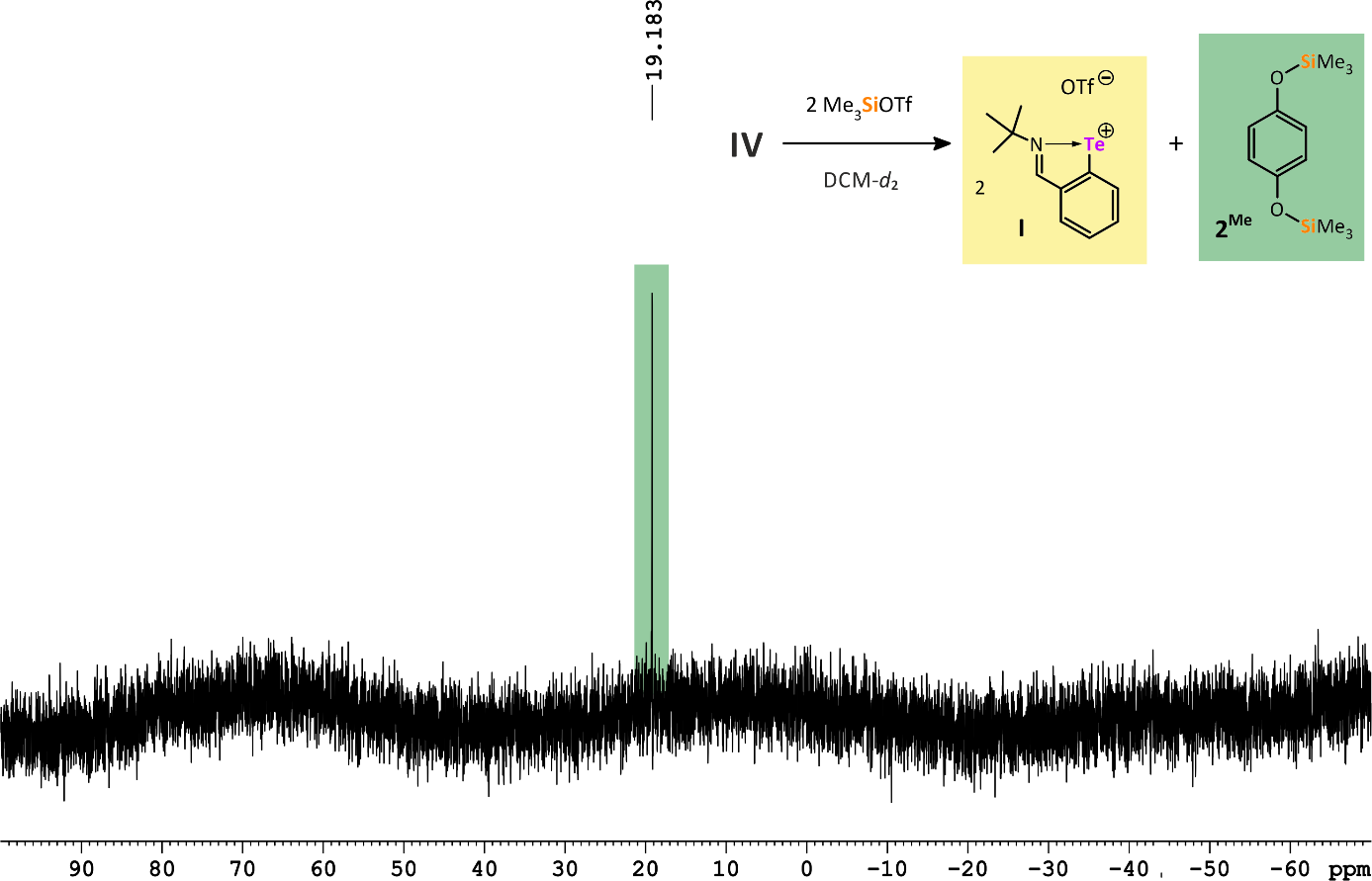


Figure S33: ^29^Si{^1^H} NMR spectrum of rection mixture from reaction of IV with 2 eq. of Me_3_SiOTf in DCM-*d*_2_ (500.20 MHz, 295 K).

On the other hand, unlike to **IIa**, isolated sample of di-iminium ditelluride **IIb** reacts with ***p*-q** very rapidly. In less than 30 seconds, the reaction is done (as judged by discoloration of blood red **IIb** to yellow **I**) and 2 eq. of tellurenyl species **I** next to 1. eq of hydroquinone is quantitatively formed (see **Figure S34** below and **Scheme S11** above):


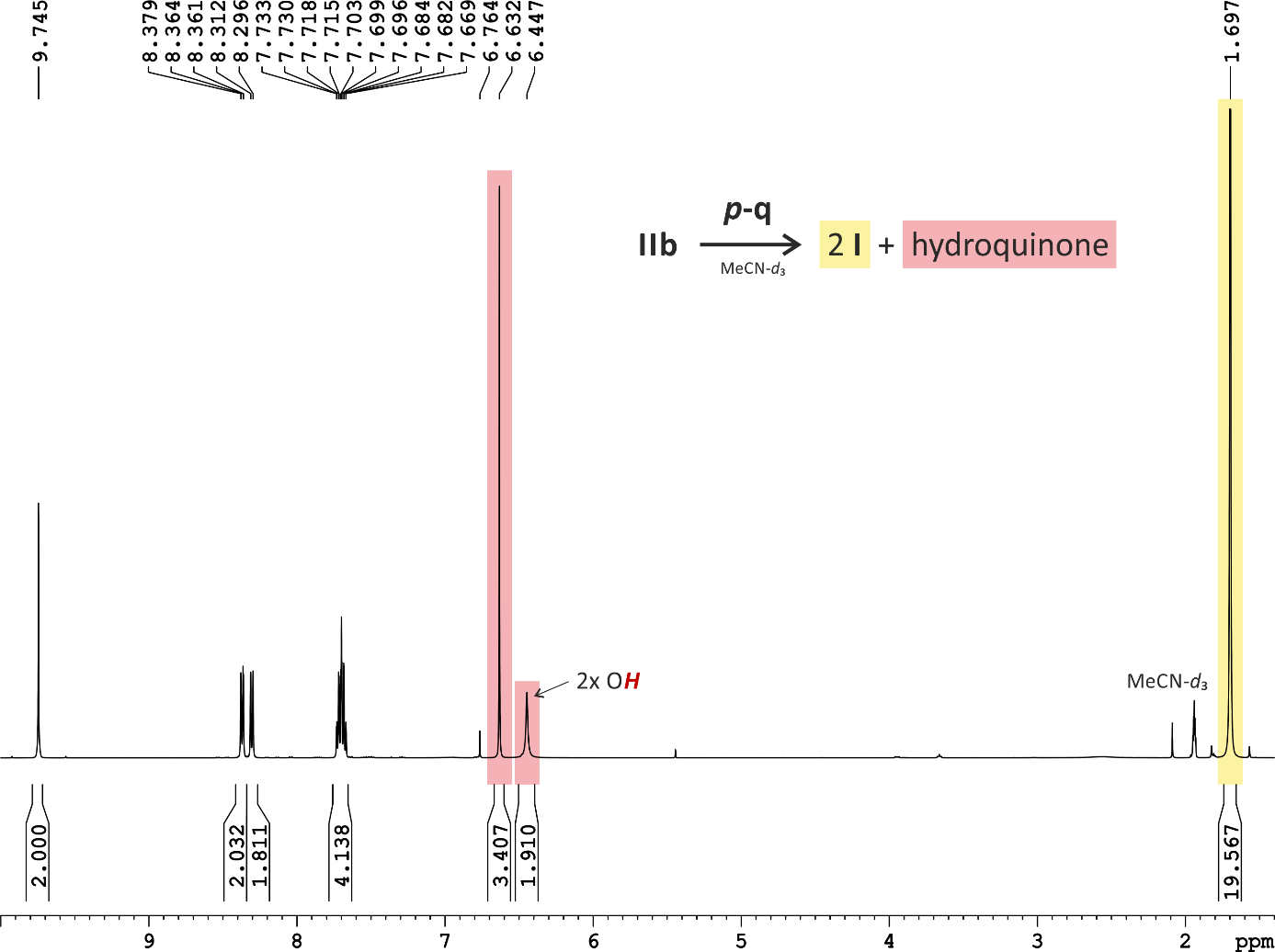


Figure S34: ^1^H NMR spectrum of rection mixture from reaction of IIb with *p*-q in 1:1 molar ratio in MeCN-*d*_3_ (500.20 MHz, 295 K).

To sum up, involvement of **IIa** and **IIb** (formed *in-situ* from **II** by autoionization – a proton scrambling shown in **Scheme S10**) in the catalytic cycle presented in the main text is highly improbable as deprotonated **IIa** reacts with ***p*-q** only extremely slowly into **IV**. Even though, on contrary, **IIb** reacts with ***p*-q** very quickly, the proton scrambling shown in the **Scheme S10** assures that for each molecule of **IIb** there must be a molecule of **IIa**, i.e. kinetics of reaction **IIa** with ***p*-q** is the limiting factor. On the other hand, after addition of silyl triflate, sum of products from both isolated reactions are the exact same products as by using only mono-iminium ditelluride **II** (see **Figure S35** and following figures below), thus, there is no impact of potential involvement of **IIa** and **IIb** on the mechanism outlined in **Scheme 7** in the main text.

*Final note*: There is no wonder why the abovementioned reaction of compound **IV** with Me_3_SiOTf and HOTf leads to the same products as when **IIIa** is used instead of compound **IV**. This is due to the fact, that compounds **IV** and **IIIa** are structurally related species, and one species can be turned into the second one only by addition/elimination of two molecules of **I**. This hypothesis was unambiguously proven by reaction of **IV** with 2 eq. of **I**, resulting in a quantitative formation of **IIIa** (**Scheme S13**).


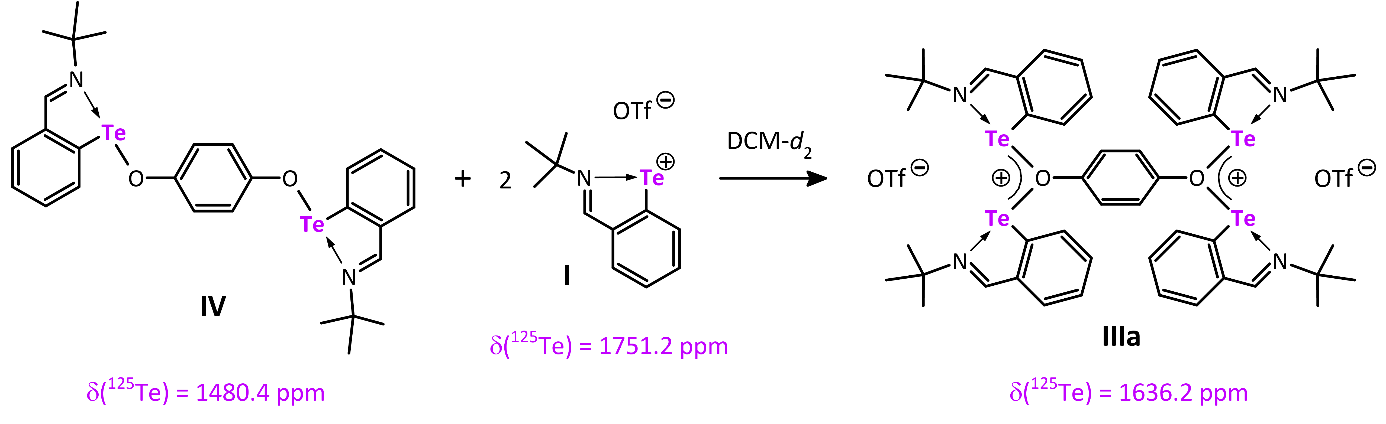


Scheme S13: Reactivity of isolated IV with 2 eq. of I leading to IIIa.

NMR spectra from stoichiometric reaction of 1:1 mixture
of **II** and Et_3_SiOTf with ***p*-q**


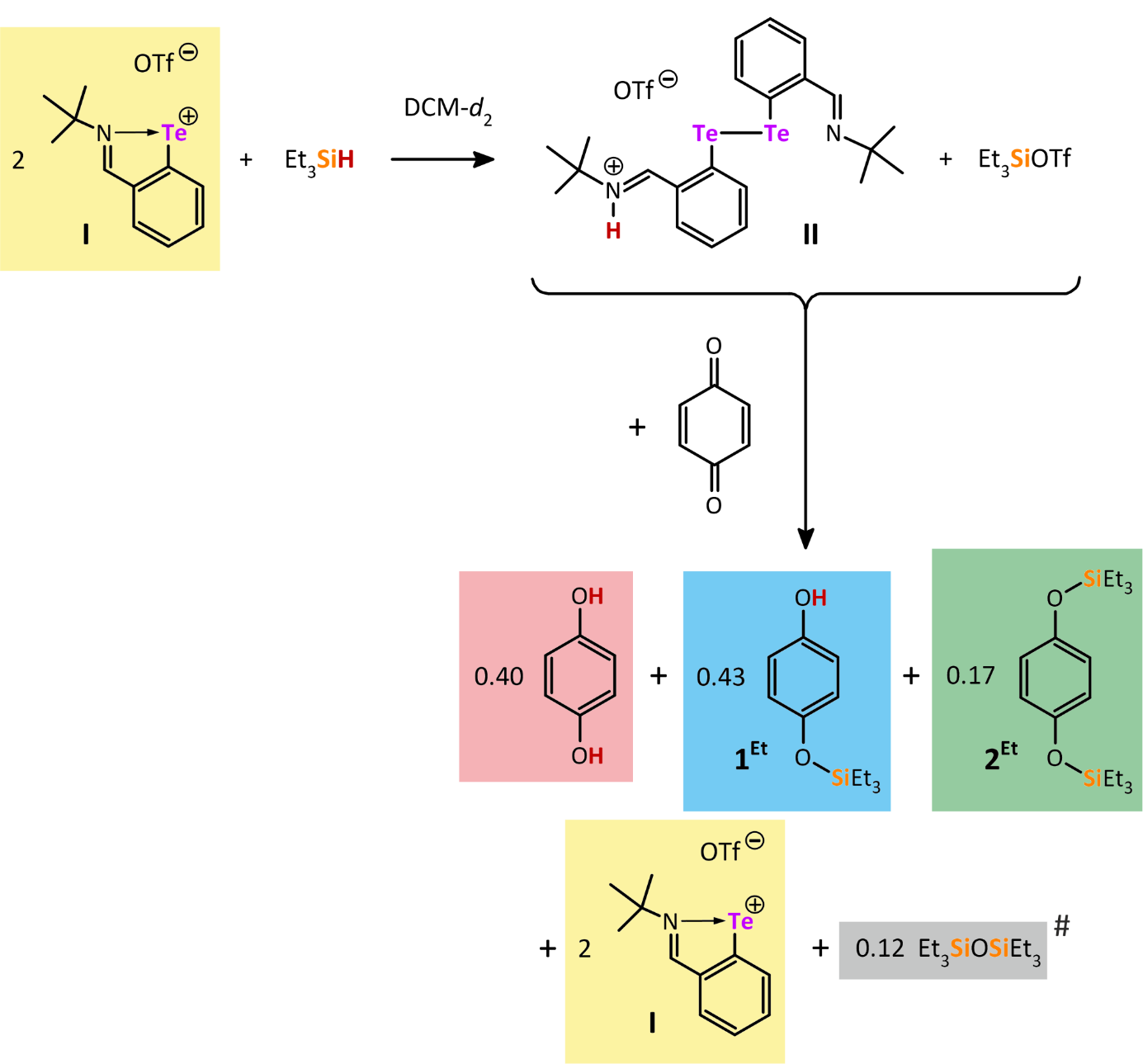


Scheme S14

^#^ At stoichiometric conditions, we faced a non-negligible hydrolysis of the generated Et_3_SiOTf resulting in formation of siloxane of type Et_3_SiOSiOEt_3_. Consequently, this affected the final molar ratio of silylated products **1^Et^** and **2^Et^** *vs* hydroquinone, favoring the hydroquinone. In contrast, at catalytic conditions, hydrolysis is basically non-observable. At these conditions, it is probably suppressed due to a rapid consumption of *in-situ* generated silyl triflate in the catalytic cycle, given large excess of present oxidant (***p*-q**) *vs* the generated silyl triflate.


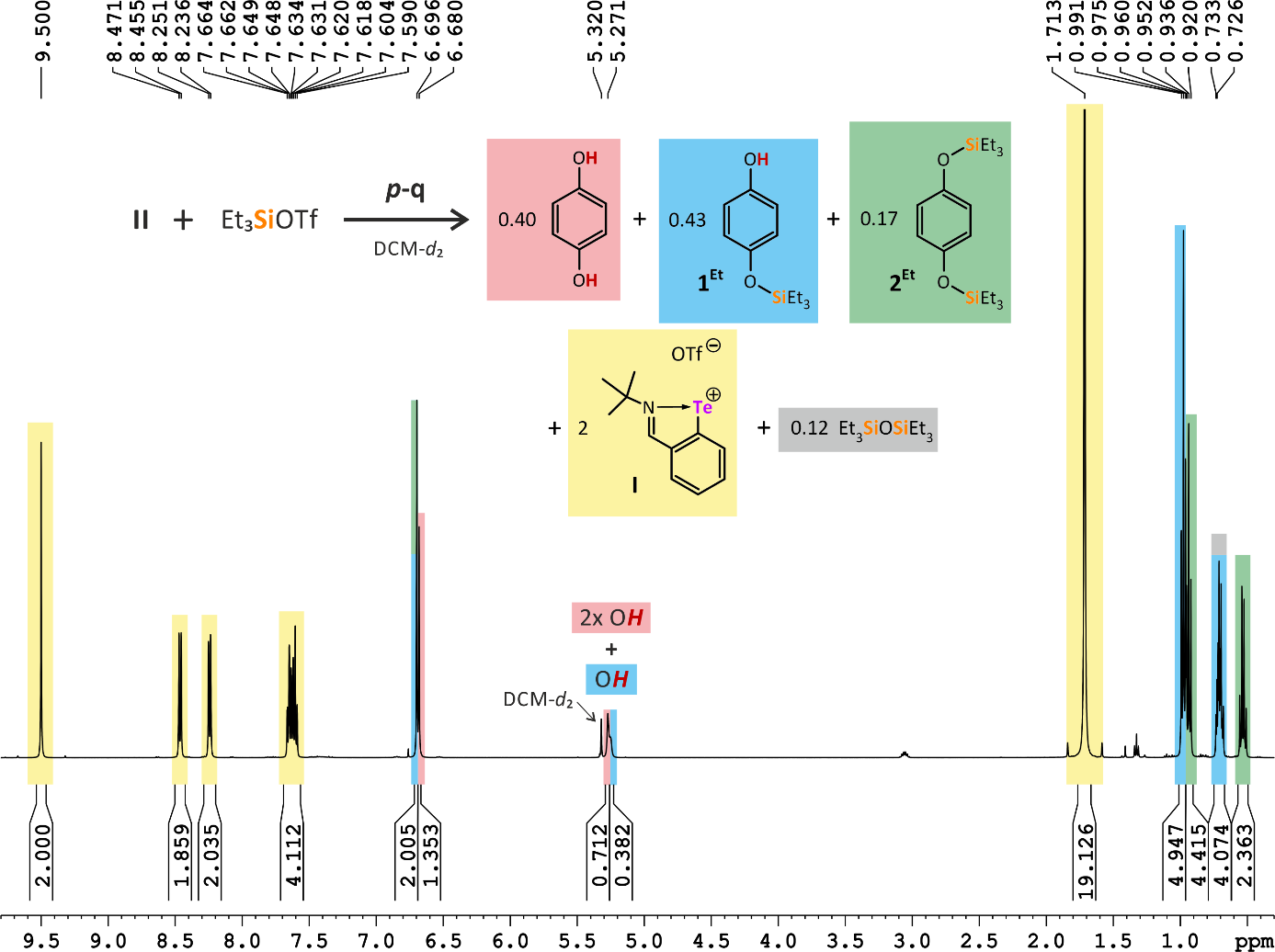


Figure S35: ^1^H NMR spectrum of obtained reaction mixture after addition of 1 eq. of *p*-q to a 1:1 mixture of II and Et_3_SiOTf in DCM-*d*_2_ (500.20 MHz, 295 K).


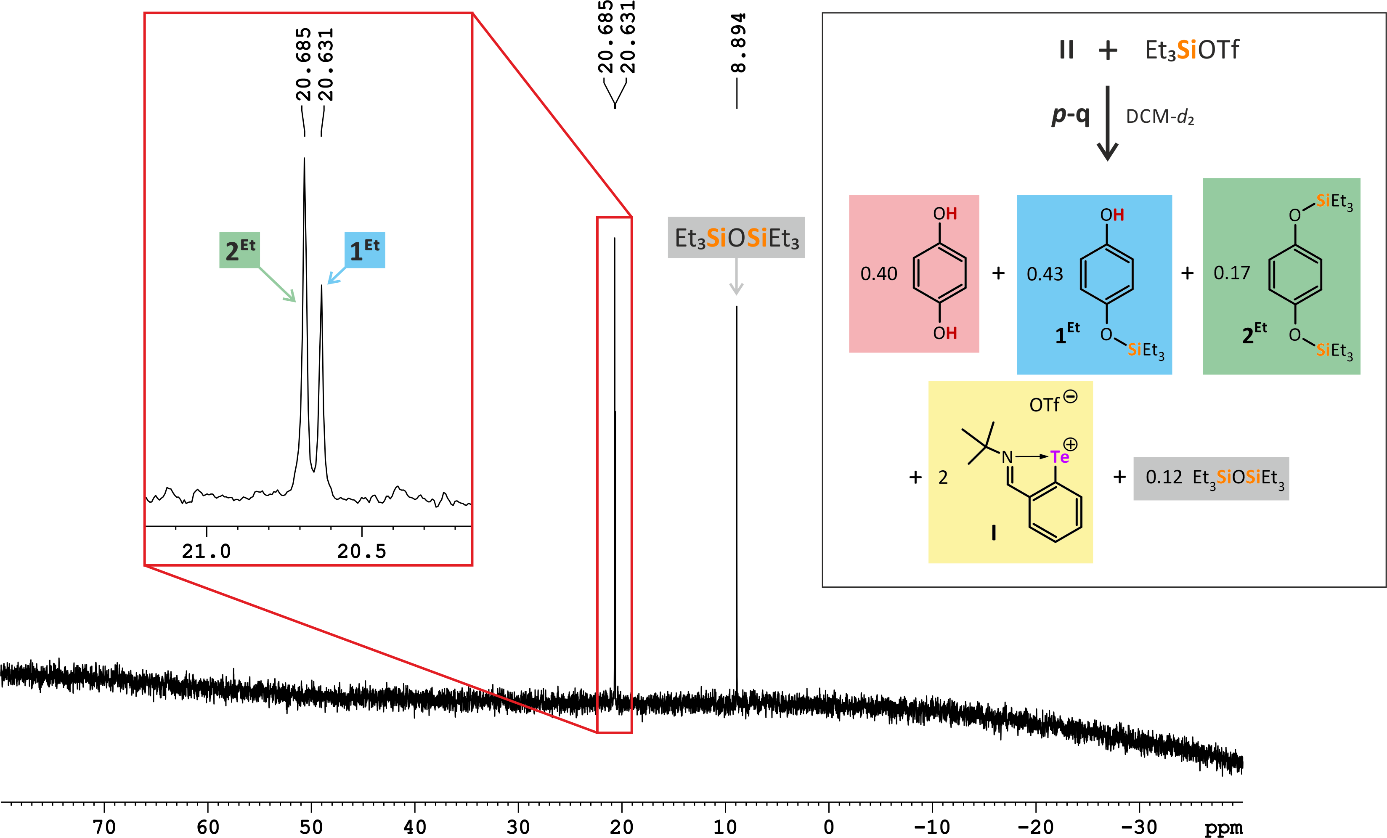


Figure S36: ^29^Si{^1^H} NMR spectrum of obtained reaction mixture after addition of 1 eq. of *p*-q to a 1:1 mixture of II and Et_3_SiOTf in DCM-*d*_2_ (99.37 MHz, 295 K).

NMR spectra from reaction of **II** with ***p*-q** leading to **IIIa** and hydroquinone


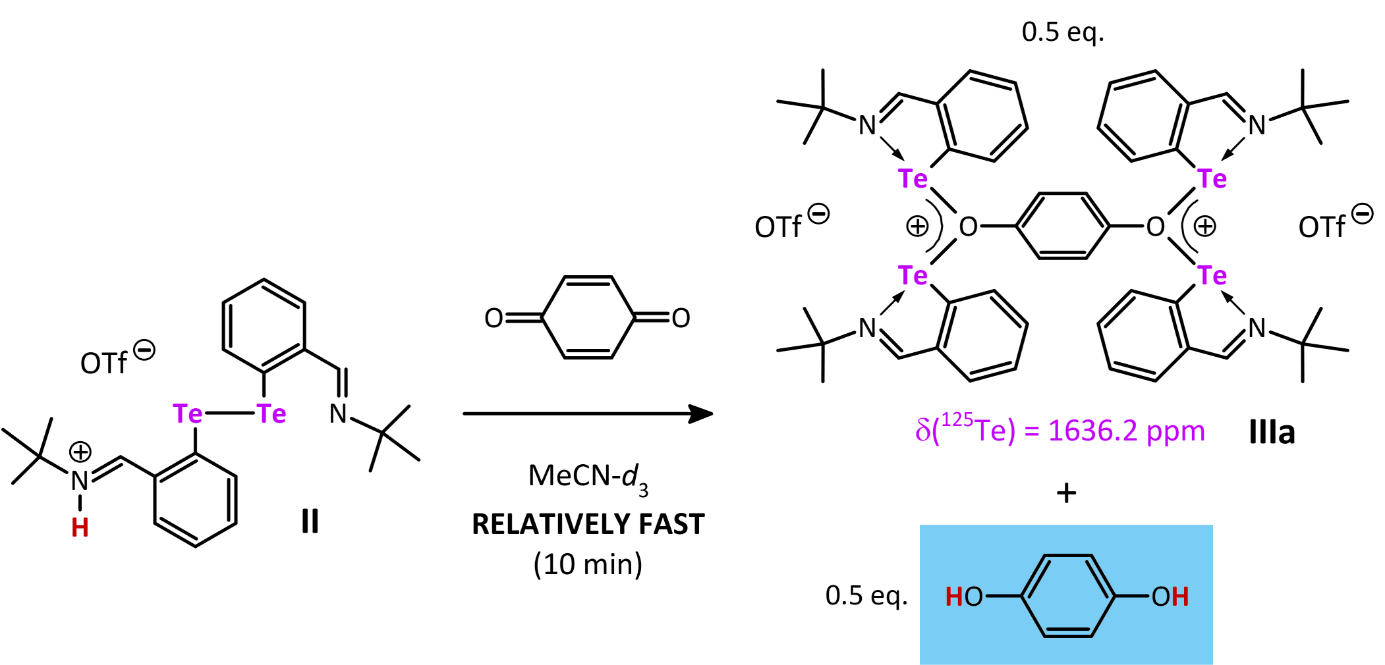


Scheme S15

0.370 mmol of compound **II** was *in situ* synthetized in a Schlenk tube from 213 mg (0.370 mmol) of **IIa** and 33 μL (0.370 mmol) of HOTf in dry and degassed MeCN-*d*_3_ (3 mL) according to a **Scheme S1**. Then, the obtained blood red solution of **II** was transferred *via* canula to another Schlenk tube containing 40 mg (0.370 mmol) of 1,4-benzoquinone (***p*-q**) under vigorous stirring (**Scheme S15**). Immediately after mixing, the blood-red color of the solution turned into yellow. After about 20 minutes of stirring, a sample of the reaction mixture was transferred to NMR tube, flame-sealed and analyzed, proving formation of 1:1 molar mixture of **IIIa** and hydroquinone (see spectra below). By storing the concentrated solution of the reaction mixture overnight at 6 °C, light-yellow single-crystals of **IIIa** were obtained (190 °C decomp.).


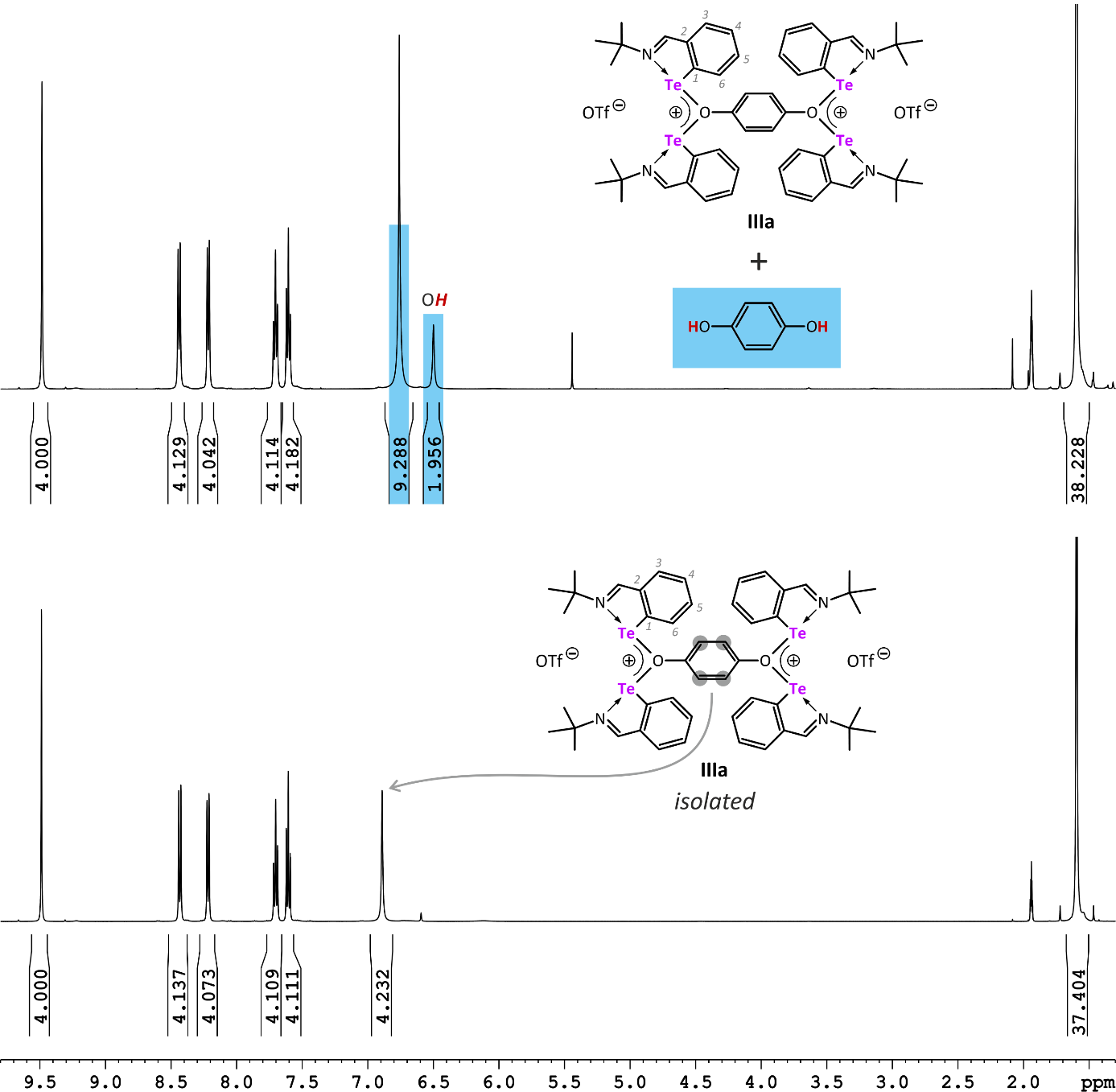


Figure S37: Stacked plot of ^1^H NMR spectra of 1 : 1 molar mixture of bis(oxonium) species IIIa and *para*-hydroquinone, formed from the reaction of II and *p*-q(*t*Bu)_2_ (top spectrum) along with spectrum of isolated bis(oxonium) species IIIa (bottom spectrum) (500.20 MHz, 295 K, MeCN-*d*_3_).


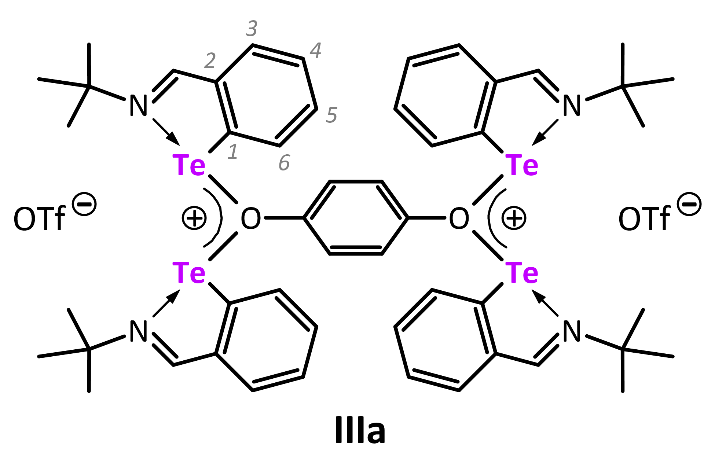


*NMR data for* ***IIIa*** *in MeCN-d_3_:*

**^1^H NMR** (500.20 MHz, MeCN-*d*_3_) δ (ppm): 1.59 [36H, s, 4x (C*H*_3_)_3_C-]; 6.89 [4H, s, O-C_6_*H_4_*-O]; 7.61 [4H, td, 4x Ar(C4)*H*]; 7.70 [4H, td, 4x Ar(C5)*H*]; 8.22 [4H, d, 4x Ar(C3)*H*]; 8.43 [4H, d, 4x Ar(C6)*H*]; 9.49 [4H, s, 4x C*H*=N]. **^13^C{^1^H} NMR** (125.78 MHz, MeCN-*d*_3_) δ (ppm): 32.4 [s, (*C*H_3_)_3_C-]; 64.9 [s, qC, (CH_3_)_3_*C*-]; 121.1 [vbr. s, Ar(*C*H) of O-C_6_H_4_-O]; 122.3 [q, qC, F_3_*C*-, ^1^*J*(^19^F, ^13^C) = 320.6 Hz]; 128.6 [s, Ar(*C4*)H]; 132.7 [s, Ar(*C6*)H]; 133.5 [s, Ar(*C5*)H]; 134.7 [s, Ar(*C3*)H]; 138.2 [s, qC, Ar(*C1 or C2*)]; 142.6 [s, qC, Ar(*C1 or C2*)]; 158.5 [vbr. s, qC, 2x (*ipso*)*C-*OTe_2_]; 165.2 [s, CH=*N*]. **^15^N NMR** (40.54 MHz, MeCN-*d*_3_) δ: –110.0 ppm [CH=*N*🡪Te]. **^125^Te NMR** (157.79 MHz, MeCN-*d*_3_) δ: 1636.2 ppm [br. s].


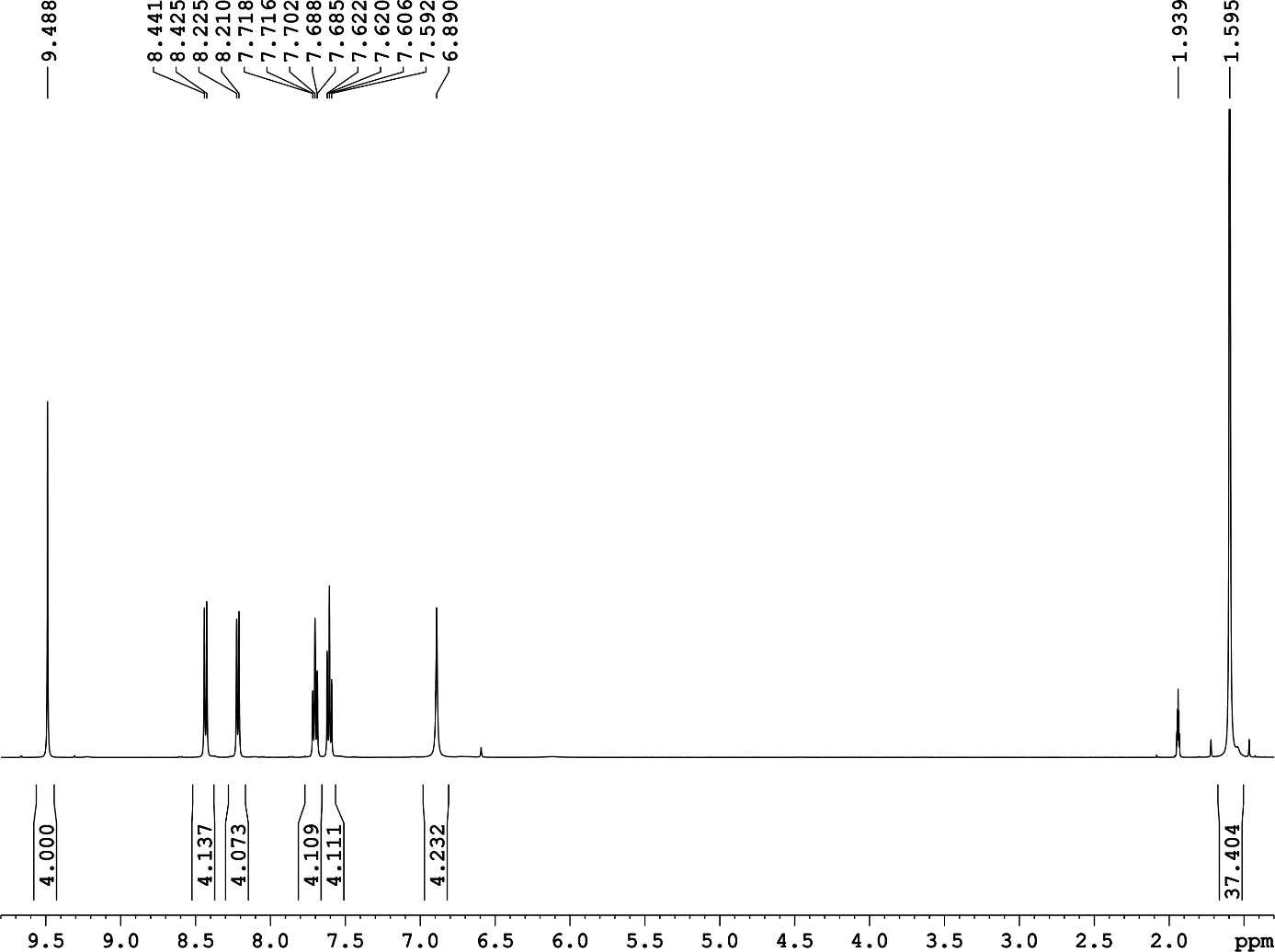

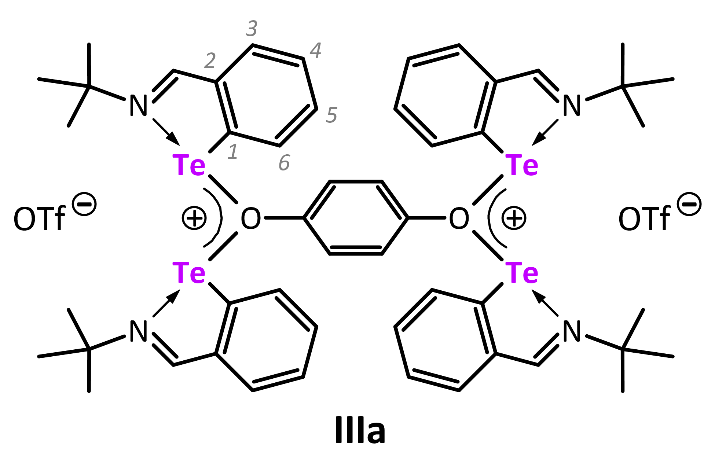


Figure S38: ^1^H NMR spectrum of isolated bis(oxonium) species IIIa in MeCN-*d*_3_ (500.20 MHz, 295 K).


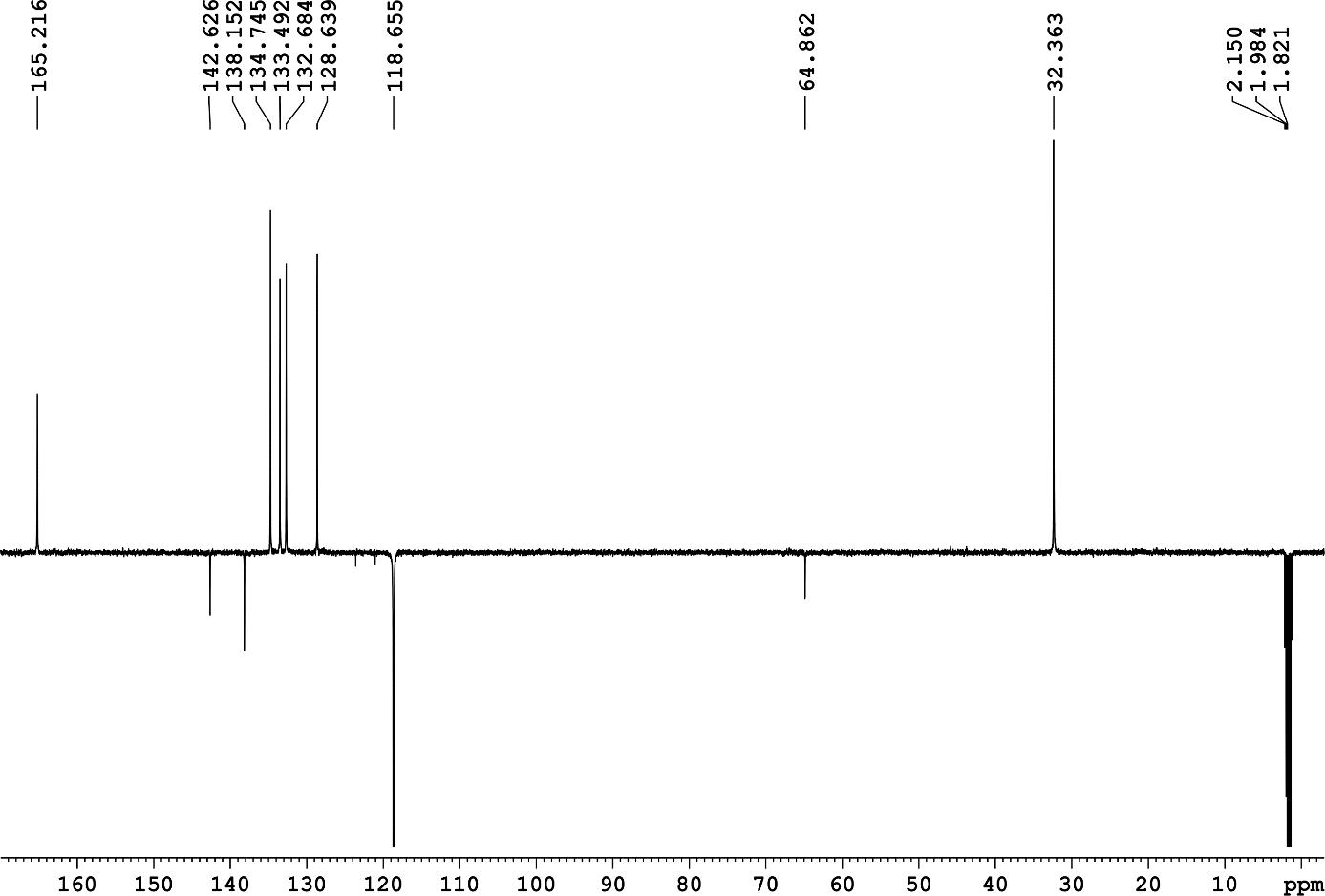

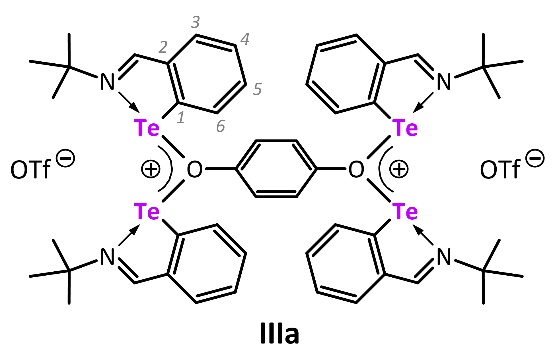


Figure S39: ^13^C{^1^H} APT NMR spectrum of isolated bis(oxonium) species IIIa in MeCN-*d*_3_ (125.78 MHz, 295 K).


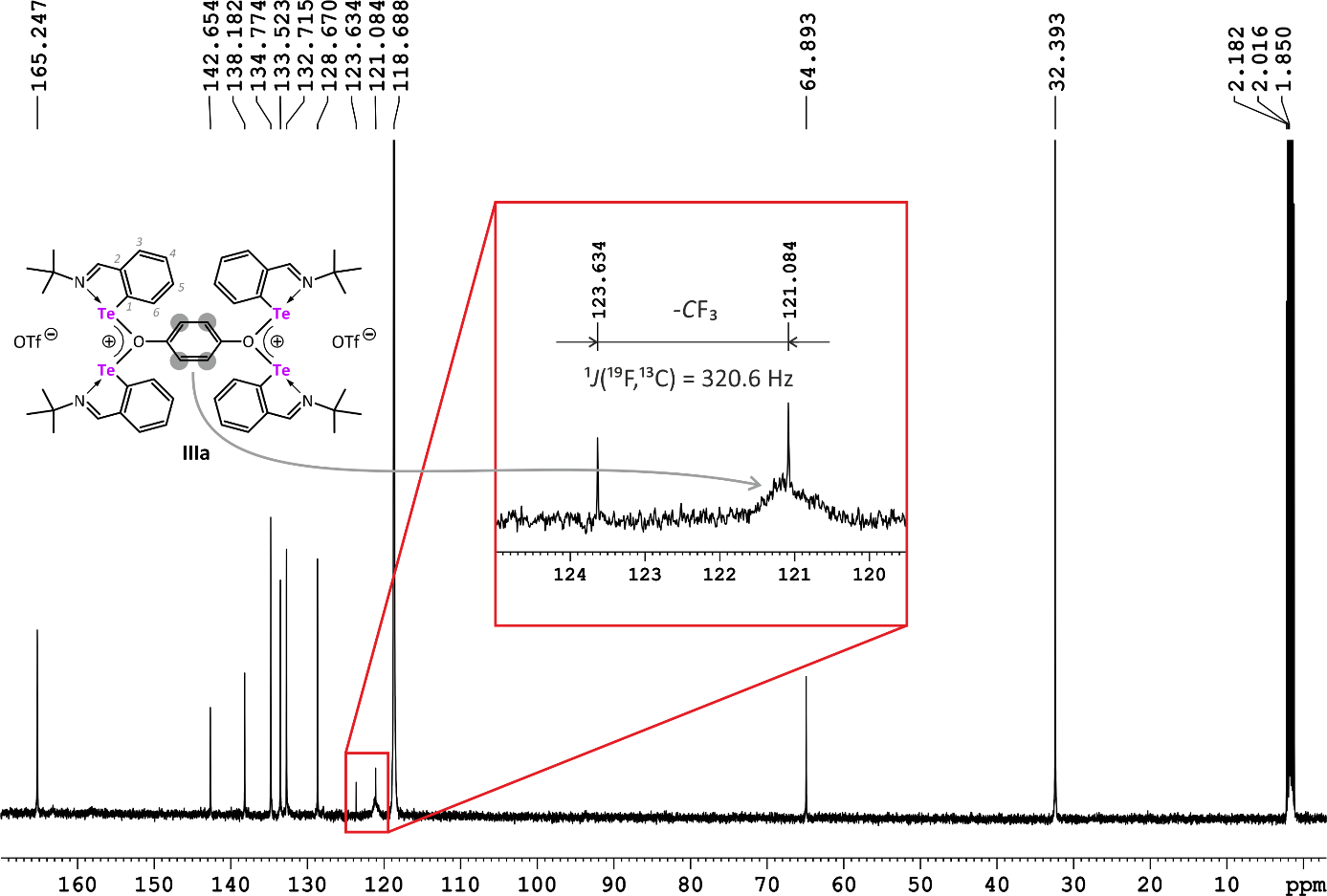


Figure S40: ^13^C{^1^H} NMR spectrum of isolated bis(oxonium) species IIIa with detail to Ar(*C*H) of O-C_6_H_4_-O moiety in MeCN-*d*_3_ (125.78 MHz, 295 K).


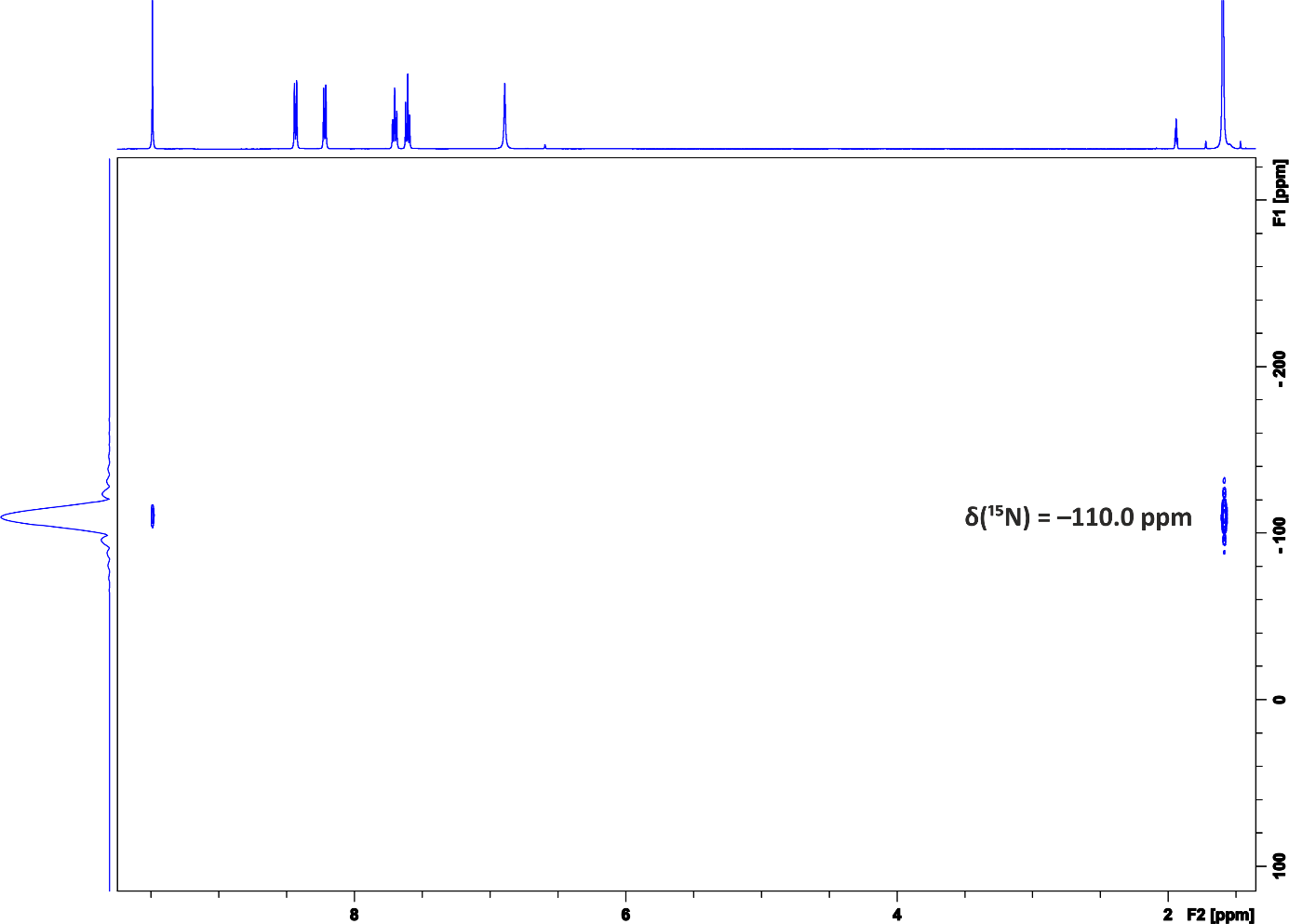

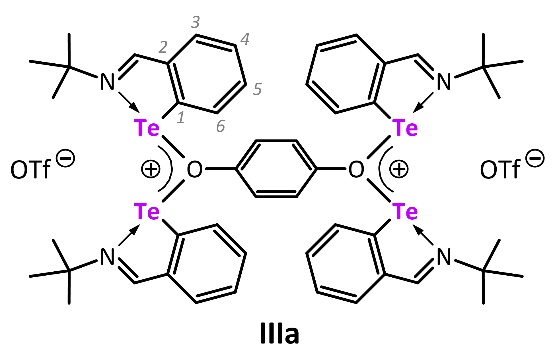


Figure S41: ^1^H-^15^N HMBC NMR spectrum of isolated bis(oxonium) species IIIa in MeCN-*d*_3_ (500.20 MHz, 295 K).


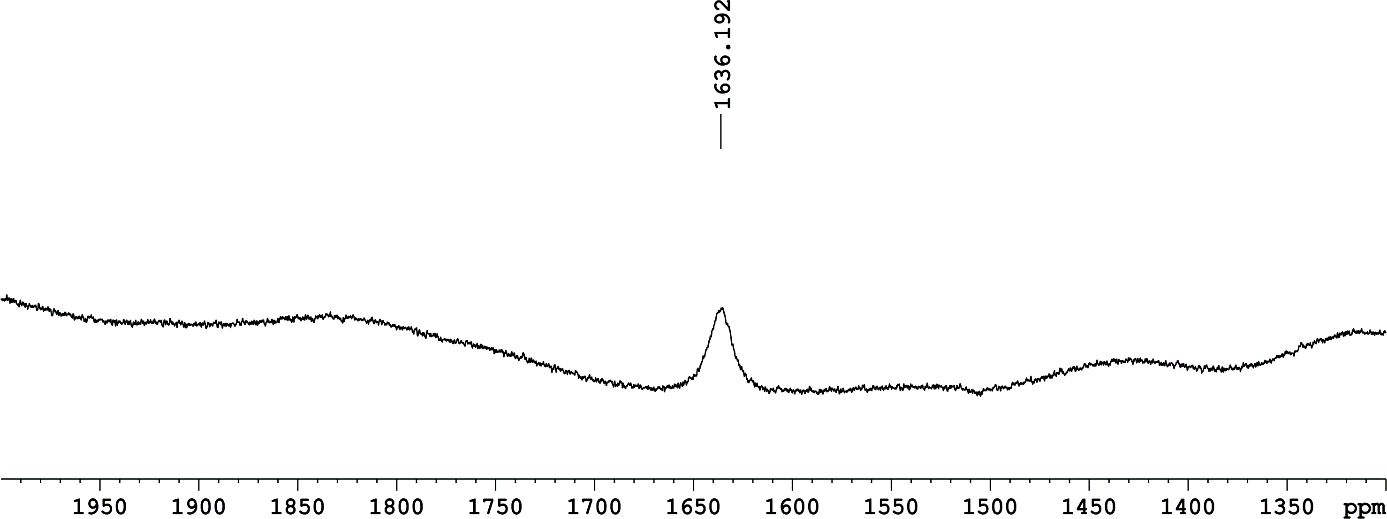


Figure S42: ^125^Te{^1^H} NMR spectrum of isolated bis(oxonium) species IIIa in MeCN-*d*_3_ (157.79 MHz, 295 K, NS = 143360).

*NMR spectra from reaction of* ***IIIa*** *with two eq. of Me_3_SiOTf*

Scheme S16

To a mixture of 0.175 mmol of compound **IIIa** and 0.175 mmol of hydroquinone, synthetized from 202 mg (0.351 mmol) of blood red **II** and 38 mg (0.351 mmol) of ***p*-q** in dry and degassed DCM-*d*_2_ (3 mL) according to a beforementioned procedure, a neat Me_3_SiOTf (64 μL, 0.351 mmol) was added under vigorous stirring (**Scheme S16**). In less than 1 minute of stirring, the color changed from yellow to an orange-yellow. A sample of the obtained reaction mixture was transferred to NMR tube, flame-sealed and analyzed. Based on this NMR analysis, two eq. of compound **I** and half eq. of compound **2^Me^** are formed next to half eq. of already presented hydroquinone (see spectra below).

Figure S43: ^1^H NMR spectrum of reaction mixture obtained after addition of 1 eq. of Me_3_SiOTf to a 1:1 mixture of IIIa and hydroquinone in DCM-*d*_2_ (500.20 MHz, 295 K).* signal of Me_3_SiOSiMe_3_ as a result of hydrolysis.

Figure S44: ^29^Si{^1^H} NMR spectrum of reaction mixture obtained after addition of 1 eq. of Me_3_SiOTf to a 1:1 mixture of IIIa and hydroquinone in DCM-*d*_2_ (99.37 MHz, 295 K). * signal of Me_3_SiOSiMe_3_ as a result of hydrolysis.

Figure S45: ^125^Te NMR spectrum of reaction obtained mixture after addition of 1 eq. of Me_3_SiOTf to a 1:1 mixture of IIIa and hydroquinone in DCM-*d*_2_ (157.79 MHz, 295 K, NS = 10240).

*NMR data for* ***2^Me^*** *in DCM-d*_2_*:*

**^1^H NMR** (500.20 MHz, DCM-*d*_2_) δ (ppm): 0.23 [18H, s, 2x (C*H*_3_)_3_Si-]; 6.69 [4H, s, Ar(C2+C3+C5+C6)*H*]. **^13^C{^1^H} NMR** (125.78 MHz, DCM-*d*_2_) δ (ppm): 0.3 [s, (*C*H_3_)_3_Si-]; 121.1 [s, 2x Ar-(*C2+C3+C5+C6*)H]; 150.0 [s, qC, Ar-(*C1+C4*)]. **^29^Si{^1^H} NMR** (99.38 MHz, DCM-*d*_2_) δ: 19.4 ppm.

NMR spectra from stoichiometric reaction of 1:1 mixture
of **II** and Et_3_SiOTf with ***p*-q(*t*Bu)_2_**

Scheme S17

Figure S46: ^1^H NMR spectrum of reaction mixture obtained after addition of 1 eq.
of *p*-q(*t*Bu)_2_ to a 1:1 mixture of II and Et_3_SiOTf in MeCN-*d*_3_ (500.20 MHz, 295 K). * traces of Et_3_SiOSiEt_3_ and of 2,6-di-tert-butylhydroquinone are presented as a result of partial hydrolysis.

Figure S47: ^29^Si{^1^H} NMR spectrum of reaction mixture obtained after addition of 1 eq.
of *p*-q(*t*Bu)_2_ to a 1:1 mixture of II and Et_3_SiOTf in MeCN-*d*_3_ (99.37 MHz, 295 K).* signal of Et_3_SiOSiEt_3_ as a result of partial hydrolysis.

NMR spectra from reaction of **II** with ***p*-q(*t*Bu)_2_** leading to **IIIb’**

Scheme S18

0.349 mmol of compound **II** was *in situ* synthetized in a Schlenk tube from 201 mg (0.349 mmol) of **IIa** and 31 μL (0.349 mmol) of HOTf in dry and degassed DCM-*d*_2_ (3 mL) according to a **Scheme S1**. Then, the obtained blood red solution of **II** was transferred *via* canula to another Schlenk tube containing 77 mg (0.349 mmol) of 2,6-di-*tert*-butyl-1,4-benzoquinone (***p*-q(*t*Bu)_2_**) under vigorous stirring (**Scheme S18**). After the addition of the blood-red solution of **II** to the yellow solution of ***p*-q(*t*Bu)_2_**, the color of the reaction mixture stayed blood-red. A sample of the obtained blood-red reaction mixture was transferred to NMR tube, flame-sealed and analyzed each next 24 hours. Based on this NMR analysis, full conversion into **IIIb’** took 5 days, which also outwardly manifested by the discoloration into a light-yellow solution. After 5 days, we attempted to grow single crystals from the remaining reaction mixture (now containing only **IIIb’**) in the Schlenk tube, however, all numerous attempts unfortunately failed. We were only capable to get small portion of yellow polycrystalline material of **IIIb’** (m.p. 159 – 161 °C).

Figure S48: Stacked plot of ^1^H NMR spectra of 1 : 1 molar mixture of mono-iminium ditelluride II and *p*-q(*t*Bu)_2_ slowly reacting into oxonium species IIIb’ (500.20 MHz, 295 K). * These signals correspond to excess of starting *p*-q(*t*Bu)_2_.

*NMR data for* ***IIIb’*** *in DCM-d*_2_*:*

**^1^H NMR** (500.20 MHz, DCM-*d*_2_) δ (ppm): 1.35 [18H, s, (C*H*_3_)_3_C-(C3’) and (C*H*_3_)_3_C-(C5’)]; 1.64 [18H, s, 2x (C*H*_3_)_3_C-N]; 4.83 [1H, s, O*H*]; 6.84 [2H, s, Ar(C2’)*H* and Ar(C6’)*H*]; 7.59 [2H, t, 2x Ar(C4)*H*]; 7.60 [2H, t, 2x Ar(C5)*H*]; 8.22 [2H, br. d, 2x Ar(C3)*H*]; 8.38 [2H, d, 2x Ar(C6)*H*]; 9.46 [2H, s, 2x C*H*=N]. **^13^C{^1^H} NMR** (125.78 MHz, DCM-*d*_2_) δ (ppm): 30.4 [s, (*C*H_3_)_3_C-C3’ and (*C*H_3_)_3_C-C5’]; 32.1 [s, (*C*H_3_)_3_C-N]; 34.9 [s, qC, (CH_3_)_3_*C*-(C3’) and (CH_3_)_3_*C*-(C5’)]; 63.6 [s, qC, (CH_3_)_3_*C*-N]; 115.5 [s, Ar-(*C4‘*)H and Ar-(*C6‘*)H]; 121.0 [q, qC, F_3_*C*-, ^1^*J*(^19^F, ^13^C) = 320.0 Hz]; 127.9 [s, Ar(*C4*)H]; 132.4 [s, Ar(*C5*)H]; 134.0 [s, Ar(*C3*)H]; 137.3 [br. s, qC, Ar(*C1* and *C2*)]; 137.8 [s, qC, (CH_3_)_3_C-(*C3’*) and (CH_3_)_3_C-(*C5’*)]; 148.2 [s, qC, *C4’-*OH]; 154.7 [vbr. s, qC, *C1’-*OTe_2_]; 162.9 [s, CH=*N*]. **^15^N NMR** (40.54 MHz, DCM-*d*_2_) δ: –107.5 ppm [CH=*N*🡪Te]. **^125^Te{^1^H} NMR** (157.79 MHz, DCM-*d*_2_) δ: 1641.3 ppm [br. s].

Figure S49: ^1^H NMR spectrum of reaction mixture of II with *p*-q(*t*Bu)_2_ forming oxonium species IIIb’ after 5 days in DCM-*d*_2_ (500.20 MHz, 295 K). * These signals correspond to excess of starting *para*-q(*t*Bu)_2_.

Figure S50: ^1^H-^1^H NOESY NMR spectrum of reaction mixture of II with *p*-q(*t*Bu)_2_ forming oxonium species IIIb’ after 5 days in DCM-*d*_2_ (500.20 MHz, 295 K, d8 = 1.6 s).

Figure S51: ^13^C{^1^H} APT NMR spectrum of reaction mixture of II with *p*-q(*t*Bu)_2_ forming oxonium species IIIb’ after 5 days in DCM-*d*_2_ (125.78 MHz, 295 K).

Figure S52: ^125^Te{^1^H} NMR spectrum of reaction mixture of II with *p*-q(*t*Bu)_2_ forming oxonium species IIIb’ after 5 days in DCM-*d*_2_ (157.79 MHz, 295 K, NS = 102400).

NMR spectra from reaction of **IIIb’** with Me_3_SiOTf

Scheme S19

To 0.525 mmol of compound **IIIb’**, synthetized from 293 mg (0.509 mmol) of **II** and 112 mg (0.509 mmol) of ***p*-q(*t*Bu)_2_** in dry and degassed DCM-*d*_2_ (3 mL) according to a beforementioned procedure, neat Me_3_SiOTf (92 μL, 0.509 mmol) was added under vigorous stirring (**Scheme S19**). In less than 1 minute of stirring, the color of the reaction mixture changed from yellow color to an orange-yellow. A sample of the obtained reaction mixture was transferred to NMR tube, flame-sealed and analyzed. Based on this NMR analysis, two equiv. of compound **I** and one equiv. of compound **3^Me^** are formed (see spectra below).

Figure S53: Stacked plot of ^1^H NMR spectra of oxonium species IIIb’ in DCM-*d*_2_ (top) and after addition of 1 eq. of Me_3_SiOTf (the middle spectrum) showing regeneration of starting compound I along with formation of Me_3_Si- substituted hydroquinone 3^Me^ (500.20 MHz, 295 K). * These signals correspond to excess of starting *p*-q(*t*Bu)_2_.

Figure S54: ^29^Si{^1^H} NMR spectrum of mixture of 2 eq. of I and 1 eq. of Me_3_Si- substituted hydroquinone 3^Me^ from reaction of IIIb’ and Me_3_SiOTf in DCM-*d*_2_ (99.37 MHz, 295 K). * The signal corresponds to excess of the added Me_3_SiOTf.

*NMR data for* ***3^Me^*** *in DCM-d*_2_*:*

**^1^H NMR** (500.20 MHz, DCM-*d*_2_) δ (ppm): 0.25 [9H, s, (C*H*_3_)_3_Si-]; 1.41 [18H, s, 2x (C*H*_3_)_3_C-]; 4.82 [1H, s, O*H*]; 6.67 [2H, s, Ar(C2+C6)*H*]. **^13^C{^1^H} NMR** (125.78 MHz, DCM-*d*_2_) δ (ppm): 0.4 [s, (*C*H_3_)_3_Si-]; 30.5 [s, 2x (*C*H_3_)_3_C-]; 34.8 [s, qC, (CH_3_)_3_*C*-]; 116.5 [s, 2x Ar-(*C2+C6*)H]; 137.8 [s, qC, Ar-(*C3+C5*)]; 148.3 [s, qC, Ar-(*C1*)]; 148.6 [s, qC, Ar-(*C4*)]. **^29^Si{^1^H} NMR** (99.38 MHz, DCM-*d*_2_) δ: −18.1 ppm.

NMR spectra from stoichiometric activation of Si-H bond in compound **7** – formation of silyl triflate **Ph_2_SiOTf(O-C_6_H_2_(tBu)_2_OH)**

Scheme S20

484 mg (1.108 mmol, 2 eq.) of compound **I** (light-orange crystalline solid) and 224 mg (0.554 mmol, 1 eq.) of compound **7** (colorless crystalline solid) were loaded into a Schlenk tube under argon atmosphere. Content of the Schlenk tube was dissolved in DCM-*d*_2_ (3 mL) under stirring while the obtained solution immediately turned to blood red indicating the formation of **II** (**Scheme S20**). After 10 minutes of stirring, a sample of the dark red solution was transferred to NMR tube, flame-sealed and analyzed. Based on this NMR analysis, full conversion into products **II** and **Ph_2_SiOTf(O-C_6_H_2_(tBu)_2_OH** took full 4 days.

*NMR data for* ***Ph_2_SiOTf(O-C_6_H_2_(tBu)_2_OH)*** *in DCM-d_2_:*

**^1^H NMR** (500.20 MHz, DCM-*d*_2_) δ (ppm): 1.34 [18H, s, 2x (C*H*_3_)_3_C-]; 4.96 [1H, s, O*H*]; 6.78 [2H, s, Ar(C2+C6)*H*]; 7.52 [4H, t, *m*-Ph_2_Si]; 7.63 [2H, tt, *p*-Ph_2_Si]; 7.80 [4H, dd, *o*-Ph_2_Si]. **^13^C{^1^H} NMR** (125.78 MHz, DCM-*d*_2_) δ (ppm): 30.2 [s, 2x (*C*H_3_)_3_C-]; 34.8 [s, qC, (CH_3_)_3_*C*-]; 116.6 [s, 2x Ar-(*C2+C6*)H]; 129.0 [s, *o*-*C*H, Ph_2_Si]; 131.6 [qC, Ph(*ipso-C*)]; 133.1 [s, *p*-*C*H, Ph_2_Si]; 135.6 [s, *o*-*C*H, Ph_2_Si]; 137.9 [s, qC, Ar-(*C3+C5*)]; 145.5 [s, qC, Ar-(*C1*)]; 150.0 [s, qC, Ar-(*C4*)]. **^29^Si{^1^H} NMR** (99.38 MHz, C_6_D_6_) δ: −30.5 ppm.

Figure S55: Stacked plot of ^1^H NMR spectra of reaction mixture of II and Ph_2_SiOTf(O-C_6_H_2_(*t*Bu)_2_OH) formed from reaction of 2 eq. of I and 7 in DCM-*d*_2_ after 4 days (top) and, for comparison, spectrum of reaction mixture of II and Et_3_SiOTf formed from analogous reaction of 2 eq. of I and Et_3_SiH in DCM-*d*_2_ after 10 minutes (500.20 MHz, 295 K). The bottom spectrum was already published by us.^[6]^

Figure S56: ^13^C{^1^H} APT NMR spectrum of reaction mixture of II and Ph_2_SiOTf(O-C_6_H_2_(*t*Bu)_2_OH) formed from reaction of 2 eq. of I and 7 in DCM-*d*_2_ (99.37 MHz, 295 K).

Figure S57: ^29^Si{^1^H} (top) and ^29^Si (bottom) NMR spectrum of reaction mixture of II and Ph_2_SiOTf(O-C_6_H_2_(*t*Bu)_2_OH) formed from reaction of 2 eq. of I and 7 in DCM-*d*_2_. It should be noted that unlike spectrum for the starting compound 7 (which contains a Si-H bond as shown in Figure S17), the non-decoupled ^29^Si spectrum does not show ^1^*J*(^29^Si,^1^H) coupling interaction, thus proves that the formed product of the reaction does not contain a Si-H bond (99.37 MHz, 295 K).

2D-FTIR correlation maps

2D-FTIR maps were evaluated applying **Noda’s rules**[^[11]^](#_ENREF_6)**:**

- If Φ(ν_1_, ν_2_) > 0 and Ψ(ν_1_, ν_2_) > 0 or Φ(ν_1_, ν_2_) < 0 and Ψ(ν_1_, ν_2_) < 0, then the change at ν_1_ precedes that of ν_2_ (ν_1_ → ν_2_).
- If Φ(ν_1_, ν_2_) > 0 and Ψ(ν_1_, ν_2_) < 0 or if Φ(ν_1_, ν_2_) < 0 and Ψ(ν_1_, ν_2_) > 0, then the change at ν_1_ follows that of ν_2_ (ν_1_ ← ν_2_).
- If Φ(ν_1_, ν_2_) > 0 and Ψ(ν_1_, ν_2_) = 0 or if Φ(ν_1_, ν_2_) < 0 and Ψ(ν_1_, ν_2_) = 0, then the movements of ν_1_ and ν_2_ are simultaneous (ν_1_ ≈ ν_2_).
- If Φ(ν_1_, ν_2_) = 0, then sequential order cannot be determined.

**Table S2:** 2D correlation data and results for the studied catalytic reactions corresponding to 2D correlation maps shown in as **Figures S58-63** and corresponding also to plots shown in **Figure 3** in the manuscript. ^a^ Symbols + and – represent positive and negative correlation coefficients, respectively; 0 means that no or not significant correlation was observed.

| Catalyzed reaction using  1 mol. % of **I** | Correlation data from 2DCoS maps^a^ | | | | |  | |  | |
| --- | --- | --- | --- | --- | --- | --- | --- | --- | --- |
| Et_3_SiH **+ *p*-q**  🡪  0.25 hydroquinone + 0.5 **1^Et^** + 0.25 **2^Et^** | **Φ/Ψ** | 1657 | 1246 | 815 | variable ν_1_ [cm^-1^] | |  | | |
|  | 1657 | +/0 | -/0 | +/+ |  | |  | | |
|  | 1246 | -/0 | +/0 | -/- |  | |  | | |
|  | 815 | +/- | -/+ | +/0 |  | |  | | |
|  | variable ν_2_ [cm^-1^] |  |  |  |  | |  | | |
|  |  | **Resulting sequence: 815 → 1657 ≈ 1246** | | | | | | |  |
| Et_3_SiH + ***p*-q(tBu)_2_**  🡪 **3^Et^** | **Φ/Ψ** | 1656 | 994 | 815 | variable ν_1_ [cm^-1^] | |  | | |
|  | 1657 | +/0 | -/0 | +/+ |  | |  | | |
|  | 994 | -/0 | +/0 | -/- |  | |  | | |
|  | 815 | +/- | -/+ | +/0 |  | |  | | |
|  | variable ν_2_ [cm^-1^] |  |  |  |  | |  | | |
|  |  | **Resulting sequence: 815 → 1656 ≈ 994** | | | | | | | |
| Ph_2_SiH_2_  + 2 ***p*-q(*t*Bu)_2_**  🡪 **6** | **Φ/Ψ** | 1656 | 995 | 846 | 826 | | variable ν_1_ [cm^-1^] | | |
|  | 1656 | +/0 | -/+ | +/+ | -/+ | |  | | |
|  | 995 | -/- | +/0 | -/- | +/+ | |  | | |
|  | 846 | +/- | -/+ | +/0 | -/+ | |  | | |
|  | 826 | -/- | +/- | -/- | +/0 | |  | | |
|  | variable ν_2_ [cm^-1^] |  |  |  |  | |  | | |
|  |  | **Resulting sequence: 846 → 1656 → 826 → 995** | | | | | | | |

**Figure S58:** Synchronous (Φ) 2D-correlation map for the reaction of Et_3_SiH with ***p*-q** catalyzed by 1 mol. % of **I** in MeCN leading to mixture of hydroquinone, **1^Et^** and **2^Et^** in 0.25:0.5:0.25 molar ratio. Red and blue areas represent positive and negative correlation intensity, respectively.

**Figure S59:** Asynchronous (Ψ) 2D-correlation map for the reaction of Et_3_SiH with ***p*-q** catalyzed by 1 mol. % of **I** in MeCN leading to mixture of hydroquinone, **1^Et^** and **2^Et^** in 0.25:0.5:0.25 molar ratio. Red and blue areas represent positive and negative correlation intensity, respectively.

**Figure S60:** Synchronous (Φ) 2D-correlation map for the reaction of Et_3_SiH with ***p*-q(*t*Bu)_2_** catalyzed by 1 mol. % of **I** in MeCN leading to **3^Et^**. Red and blue areas represent positive and negative correlation intensity, respectively.

**Figure S61:** Asynchronous (Ψ) 2D-correlation map for the reaction of Et_3_SiH with ***p*-q(*t*Bu)_2_** catalyzed by 1 mol. % of **I** in MeCN leading to **3^Et^**. Red and blue areas represent positive and negative correlation intensity, respectively.

**Figure S62:** Partial (reaction time 0–90 minutes) synchronous (Φ) 2D-correlation map for the reaction of Ph_2_SiH_2_ with ***p*‑q(tBu)_2_** in 1:2 molar ratio catalyzed in MeCN by 1 mol. % of **I** vs. a Si-H bond leading to **6** via transient formation of **7**. Red and blue areas represent positive and negative correlation intensity, respectively.

**Figure S63:** Partial (reaction time 0–90 minutes) asynchronous (Ψ) 2D-correlation map for the reaction of Ph_2_SiH_2_ with ***p*‑q(tBu)_2_** in 1:2 molar ratio catalyzed in MeCN by 1 mol. % of **I** vs. a Si-H bond leading to **6** via transient formation of **7**. Red and blue areas represent positive and negative correlation intensity, respectively.

***DFT Computations***

**Computational methodology.** Geometry Optimizations of the isolated molecule structures were carried out using density functional theory (DFT) at the B3PW91/6-311+G(2df,p)^[^[^26]^](#_ENREF_8) level of theory using the Gaussian16^[27]^ software package. For the Te atom, effective core potentials (ECP28MDF) and corresponding cc-pVTZ basis sets^[28]^ were used. Dispersion effects were modelled using Grimme’s GD3BJ parameters.^[29]^ The dependence of the geometry and thermochemistry data on potential solvent effects has been tested by including the polarizable continuum model (IEF-PCM^[30]^ and universal force field (UFF) atomic radii) with acetonitrile as solvent for the geometry optimization at the level of theory specified above. The wavefunction file of the gas-phase optimized structure was used for a topological analysis of the electron density according to the Atoms-In-Molecules partitioning scheme^[21]^ using AIMAll.^[31]^ The NCI^[22]^ grids were computed with NCIplot.^[32]^ Figures are displayed using Multiwfn^[33]^ and VMD.^[34]^

**Table S3.** Thermochemistry data [in hartree] derived from the optimized geometries using the pcm model with acetonitrile are given in italics.

|  |  | | |
| --- | --- | --- | --- |
| **E** | –1883.0123  *–1883.0681* | –3383.2877  *–3383.4320* | –382.6941  *–382.7045* |
| **ZPE** | 0.5493  *0.5492* | 0.9897  *0.9893* | 0.1086  *0.1086* |
| **E+ZPE** | –1882.4630  *–1882.5189* | –3382.2980  *–3382.4427* | –382.5855  *–382.5959* |
| **H** | –1882.4263  *–1882.4824* | –3382.2322  *–3382.3772* | –382.5776  *–382.5882* |
| **G** | –1882.5356  *–1882.5907* | –3382.4119  *–3382.5553* | –382.6161  *–382.6264* |

**Table S4.** Thermochemistry data [in hartree] derived from the optimized geometries using the pcm model with acetonitrile are given in italics.

|  |  | | |
| --- | --- | --- | --- |
| **E** | –2197.5503  *–2197.6023* | –3697.8154  *–3697.9602* | –697.2266  *–697.2349* |
| **ZPE** | 0.7737  *0.7727* | 1.2152  *1.2149* | 0.3331  *0.3324* |
| **E+ZPE** | –2196.7767  *–2196.8296* | –3696.6002  *–3696.7453* | –696.8936  *–696.9025* |
| **H** | –2196.7288  *–2196.7816* | –3696.5241  *–3696.6695* | –696.8747  *–696.8835* |
| **G** | –2196.8617  *–2196.9149* | –3696.7198  *–3696.8622* | –696.9369  *–696.9459* |

**Table S5.** AIM derived topological bond properties of selected bonds of the bis(oxonium) species **IIIa**.

| **Species** | **d**  **[Å]** | **ρ(r)**  **[eÅ^-3^]** | **∇^2^ρ(r)**  **[eÅ^-5^]** | **ε** | **G/ρ(r)**  **[a.u.]** | **H/ρ(r)**  **[a.u.]** | **δ** |
| --- | --- | --- | --- | --- | --- | --- | --- |
| **Te1-O5** | 2.307 | 0.42 | 3.7 | 0.11 | 0.80 | –0.18 | 0.46 |
| **Te1-N18** | 2.168 | 0.64 | 4.1 | 0.25 | 0.83 | –0.38 | 0.74 |
| **Te2-O5** | 2.289 | 0.44 | 3.7 | 0.12 | 0.80 | –0.20 | 0.48 |
| **Te2-N19** | 2.188 | 0.62 | 3.8 | 0.24 | 0.80 | –0.37 | 0.72 |
| **Te3-O6** | 2.299 | 0.43 | 3.7 | 0.12 | 0.79 | –0.19 | 0.47 |
| **Te3-N20** | 2.174 | 0.64 | 4.0 | 0.25 | 0.82 | –0.38 | 0.74 |
| **Te4-O6** | 2.298 | 0.42 | 3.7 | 0.10 | 0.80 | –0.19 | 0.47 |
| **Te4-N21** | 2.182 | 0.63 | 3.9 | 0.24 | 0.81 | –0.37 | 0.72 |
|  | **q [e]** |  | **q [e]** |  | **q [e]** |  |  |
| **Te1** | 0.98 | **O5** | –1.22 | **N18** | –1.21 |  |  |
| **Te2** | 0.94 | **O6** | –1.22 | **N19** | –1.22 |  |  |
| **Te3** | 0.96 |  |  | **N20** | –1.21 |  |  |
| **Te4** | 0.96 |  |  | **N21** | –1.22 |  |  |

***Crystallographic data for studied compounds***

Diffraction data for compounds **4**, **5**, **6**, **8**, **9** and **IIIa** were collected using a Bruker Venture D8 diffractometer at 150 K with graphite-monochromated Mo-Kα (0.7107 Å) radiation. The frames were integrated with the Bruker SAINT software package using a narrow frame algorithm. Data were corrected for absorption effects using the Multi-Scan method (SADABS). Obtained data were treated by XT-version 2014/5 and SHELXL-2017/1 software implemented in APEX4 v2022.10-0 (Bruker AXS) system.^[35]^ All non-hydrogen atoms were refined using anisotropic displacement parameters. There is one disordered *t*Bu group in **9** and it was treated by splitting of all involved atoms into two positions using standard SHELXL commands and procedure. Crystallographic data (excluding structure factors) for the structural analyses have been deposited with the Cambridge Crystallographic Data Centre, CCDC nos. 2420457 (**9**); 2420458 (**4**); 2420459 (**6**); 2420460 (**5**); 2420461 (**8**); and 2420462 (**IIIa**). Copies of this information may be obtained free of charge from The Director, CCDC, 12 Union Road, Cambridge CB2 1EZ, UK (Fax: +44-1223-336033; e-mail: deposit@ccdc.cam.ac.uk or [http://www.ccdc.cam.ac.uk](http://www.ccdc.cam.ac.uk/)).

**Table S6.** Crystal data and structure refinement.

|  | **4** | **5** | **6** |
| --- | --- | --- | --- |
| Formula | C_32_H_54_O_5_Si_2_ | C_60_H_96_O_12_Si_4_ | C_40_H_52_O_4_Si |
| Formula weight, g mol^–1^ | 574.93 | 1121.72 | 624.90 |
| Crystal system | Monoclinic | Triclinic | Triclinic |
| Crystal size, mm | 0.39 × 0.35 × 0.12 | 0.35 × 0.20 × 0.19 | 0.37 × 0.32 × 0.28 |
| Space group | P2_1_/n | P-1 | P-1 |
| *a*, Å | 6.9811(4) | 11.2739(3) | 9.7559(2) |
| *b*, Å | 9.5884(7) | 11.4037(3) | 10.8243(3) |
| *c*, Å | 24.7461(17) | 13.4399(4) | 19.6502(5) |
| *α*, º | 90 | 91.0030(10) | 93.5300(10) |
| *β*, º | 91.495(2) | 100.5180(10) | 103.6410(10) |
| *γ*, º | 90 | 109.0450(10) | 114.1960(10) |
| *V*, Å^3^ | 1655.88(19) | 1600.33(8) | 1809.84(8) |
| *Z* | 2 | 1 | 2 |
| *ρ*_calcd_, Mg m^–3^ | 1.153 | 1.164 | 1.147 |
| *μ* (Mo *Kα*), mm^–1^ | 0.143 | 0.149 | 0.103 |
| *F*(000) | 628 | 608 | 676 |
| *θ* range, deg | 1 to 26.5 | 1 to 28 | 1 to 28 |
| Index ranges | –8 ≤ h ≤ 8 | –15 ≤ h ≤ 15 | –13 ≤ h ≤ 13 |
|  | –12 ≤ k ≤ 12 | –15 ≤ k ≤ 15 | –14 ≤ k ≤ 14 |
|  | –31 ≤ l ≤ 31 | –17 ≤ l ≤ 17 | –26 ≤ l ≤ 26 |
| No. of reflns collected | 44857 | 84033 | 97663 |
| No. indep. Reflns | 3381 | 7878 | 8910 |
| No. obsd reflns with (*I>2σ(I)*) | 3323 | 7275 | 8076 |
| No. refined params | 190 | 361 | 418 |
| GooF (*F^2^*) | 1.258 | 1.054 | 1.032 |
| *R*_1_ (*F*) (*I > 2σ(I)*) | 0.098 | 0.031 | 0.037 |
| *wR*_2_ (*F*^2^) (all data) | 0.24 | 0.082 | 0.101 |
| Largest diff peak/hole, e Å^–3^ | 0.853 / –0.521 | 0.347 / –0.250 | 0.361 / –0.236 |
| CCDC | 2420458 | 2420460 | 2420459 |

*R*_int_ = ∑⏐*F*_o_^2^ - *F*_o,mean_^2^⏐/∑*F*_o_^2^, S = [∑(*w*(*F*_o_^2^ - *F*_c_^2^)^2^)/(*N*_diffrs_ - *N*_params_)]^½^ for all data, *R*(*F*) = ∑⏐⏐*F*_o_⏐ - ⏐*F*_c_⏐⏐/∑⏐*F*_o_⏐for observed data, *wR*(*F*^2^) = [∑(*w*(*F*_o_^2^ - *F*_c_^2^)^2^)/(∑*w*(*F*_o_^2^)^2^)]^½^ for all data**.**

**Table S6 (continuation).** Crystal data and structure refinement.

|  | **8** | **9** | **IIIa** |
| --- | --- | --- | --- |
| Formula | C_48_H_68_O_6_Si | C_65_H_84_O_8_Si | C_52_H_60_F_6_N_4_O_8_S_2_Te_4_ |
| Formula weight, g mol^–1^ | 769.11 | 913.32 | 1557.56 |
| Crystal system | Monoclinic | Monoclinic | Triclinic |
| Crystal size, mm | 0.59 × 0.20 × 0.18 | 0.22 × 0.20 × 0.10 | 0.33 × 0.18 × 0.10 |
| Space group | P2_1_/c | P2_1_/n | P-1 |
| *a*, Å | 11.4771(3) | 18.0733(16) | 10.4471(2) |
| *b*, Å | 14.6155(3) | 16.7578(12) | 11.1806(3) |
| *c*, Å | 27.1579(6) | 18.5239(18) | 28.1147(6) |
| *α*, º | 90 | 90 | 97.7790(10) |
| *β*, º | 96.6900(10) | 106.663(4) | 95.1400(10) |
| *γ*, º | 90 | 90 | 114.3750(10) |
| *V*, Å^3^ | 4524.54(18) | 5374.7(8) | 2924.94(12) |
| *Z* | 4 | 4 | 2 |
| *ρ*_calcd_, Mg m^–3^ | 1.129 | 1.129 | 1.769 |
| *μ* (Mo *Kα*), mm^–1^ | 0.097 | 0.094 | 2.119 |
| *F*(000) | 1672 | 1992 | 1516 |
| *θ* range, deg | 1 to 28 | 1 to 25 | 1 to 28 |
| Index ranges | –15 ≤ h ≤ 15 | –20 ≤ h ≤ 21 | –13 ≤ h ≤ 13 |
|  | –19 ≤ k ≤ 19 | –20 ≤ k ≤ 20 | –14 ≤ k ≤ 14 |
|  | –36 ≤ l ≤ 36 | –22 ≤ l ≤ 20 | –37 ≤ l ≤ 37 |
| No. of reflns collected | 117568 | 20069 | 168209 |
| No. indep. Reflns | 11220 | 9485 | 14437 |
| No. obsd reflns with (*I>2σ(I)*) | 9425 | 4571 | 13666 |
| No. refined params | 523 | 651 | 697 |
| GooF (*F^2^*) | 1.026 | 0.904 | 1.102 |
| *R*_1_ (*F*) (*I > 2σ(I)*) | 0.040 | 0.077 | 0.019 |
| *wR*_2_ (*F*^2^) (all data) | 0.099 | 0.138 | 0.043 |
| Largest diff peak/hole, e Å^–3^ | 0.345 / –0.280 | 0.257 / –0.255 | 0.852 / –0.532 |
| CCDC | 2420461 | 2420457 | 2420462 |

*R*_int_ = ∑⏐*F*_o_^2^ - *F*_o,mean_^2^⏐/∑*F*_o_^2^, S = [∑(*w*(*F*_o_^2^ - *F*_c_^2^)^2^)/(*N*_diffrs_ - *N*_params_)]^½^ for all data, *R*(*F*) = ∑⏐⏐*F*_o_⏐ - ⏐*F*_c_⏐⏐/∑⏐*F*_o_⏐for observed data, *wR*(*F*^2^) = [∑(*w*(*F*_o_^2^ - *F*_c_^2^)^2^)/(∑*w*(*F*_o_^2^)^2^)]^½^ for all data**.**

***Additional references***

[25] A. V. Protchenko, J. I. Bates, L. M. A. Saleh, M. P. Blake, A. D. Schwarz, E. L. Kolychev, A. L. Thompson, C. Jones, P. Mountford, S. Aldridge, *JACS* **2016,** *138* (13), 4555-4564.

[26] a) A. D. Becke, *J. Chem. Phys.* **1993**, *98*, 5648−5652; b) J. P. Perdew, J. A. Chevary, S. H. Vosko, K. A. Jackson, M. R. Pederson, D. J. Singh and C. Fiolhais, *Phys. Rev. B: Condens. Matter Mater. Phys.* **1992**, *46*, 6671−6687; c) R. Krishnan, J. S. Binkley, R. Seeger and J. A. Pople, *J. Chem. Phys.* **1980**, *72*, 650−654; d) A. D. McLean and G. S. Chandler, *J. Chem. Phys.* **1980**, *72*, 5639−5648.

[27] Gaussian16 (RevisionC.01), M. J. Frisch, G. W. Trucks, H. B. Schlegel, G. E. Scuseria, M. A. Robb, J. R. Cheeseman, G. Scalmani, V. Barone, G. A. Petersson, H. Nakatsuji, X. Li, M. Caricato, A. V. Marenich, J. Bloino, B. G. Janesko, R. Gomperts, B. Mennucci, H. P. Hratchian, J. V. Ortiz, A. F. Izmaylov, J. L. Sonnenberg, D. Williams-Young, F. Ding, F. Lipparini, F. Egidi, J. Goings, B. Peng, A. Petrone, T. Henderson, D. Ranasinghe, V. G. Zakrzewski, J. Gao, N. Rega, G. Zheng, W. Liang, M. Hada, M.Ehara, K. Toyota, R. Fukuda, J. Hasegawa, M. Ishida, T. Nakajima, Y. Honda, O. Kitao, H. Nakai, T. Vreven, K. Throssell, J. A. Montgomery, Jr., J. E.Peralta, F. Ogliaro, M. J. Bearpark, J. J. Heyd, E. N. Brothers, K. N. Kudin, V. N. Staroverov, T. A. Keith, R. Kobayashi, J. Normand, K. Raghavachari, A. P. Rendell, J. C. Burant, S. S. Iyengar, J. Tomasi, M. Cossi, J. M. Millam, M. Klene, C. Adamo, R. Cammi, J. W. Ochterski, R. L. Martin, K. Morokuma, O. Farkas, J. B. Foresman and D. J. Fox, Gaussian, Inc., Wallingford CT, 2019.

[28] a) B. Metz, H. Stoll and M. Dolg, *J. Chem. Phys.* **2000**, *113*, 2563-2569; b) K. A. Peterson, *J. Chem. Phys.* **2003**, *119*, 11099−11112. c) K. A. Peterson, D. Figgen, E. Goll, H. Stoll and M. Dolg, *J. Chem. Phys.* **2003**, *119*, 11113-11123.

[29] S. Grimme, S. Ehrlich and L. Goerigk, *J. Comp. Chem.* **2011**, *32*, 1456-1465.

[30] a) E. Cances, B. Mennucci, J. Tomasi, *J. Chem. Phys.* **1997**, *107*, 3032–3041; b) M. Cossi, G. Scalmani, N. Rega, V. Barone, *J. Chem. Phys.* **2002**, *117*, 43–54.

[31] AIMAll (Version 15.09.27), Todd A. Keith, TK Gristmill Software, Overland Park KS, USA, 2015 (aim.tkgristmill.com).

[32] J. Contreras-García, E. Johnson, S. Keinan, R. Chaudret, J.-P. Piquemal, D. Beratan and W. Yang, *J. Chem. Theory Comput.* **2011**, *7*, 625-632.

[33] T. Lu, F.Chen, *J. Comput. Chem.* **2012,** *33* (5), 580-592.

[34] W. Humphrey, A. Dalke and K. Schulten, *J. Mol. Graph.* **1996**, *14*, 33-38.

[35] G. M. Sheldrick, *Acta Cryst.* **2015**, *A71*, 3-8.
